# Overt and covert processing of self-relevance information in dissociative identity disorder: controlled fMRI study

**DOI:** 10.1192/bjo.2025.10914

**Published:** 2025-12-26

**Authors:** Aikaterini I. Strouza, Andrew J. Lawrence, Lora I. Dimitrova, Eline M. Vissia, Sima Chalavi, Dick J. Veltman, Antje A. T. S. Reinders

**Affiliations:** Department of Psychological Medicine, Institute of Psychiatry, Psychology & Neuroscience, King’s College London, London, UK; Department of Psychiatry, Amsterdam UMC, VU University Amsterdam, Amsterdam, The Netherlands; Heelzorg, Centre for Psychotrauma, Zwolle, The Netherlands; Movement Control and Neuroplasticity Research Group, Department of Movement Sciences, KU Leuven, Leuven, Belgium

**Keywords:** Dissociation, post-traumatic stress disorder, trauma, self-relevance processing, simulation

## Abstract

**Background:**

Dissociative identity disorder (DID) manifests with distinct trauma-avoidant and trauma-related identity states. Overtly conscious trauma-related knowledge processing is identity state-dependent. Previous research on covertly subconscious knowledge processing in DID lacks subject-specific trauma-related stimuli.

**Aims:**

Our controlled functional magnetic resonance imaging (fMRI) study explored neural and behavioural differences of overt and covert knowledge processing of individualised self-relevant words in DID.

**Method:**

Behavioural data were gathered while 56 participants underwent task-based fMRI: 14 with DID, 14 DID simulators and a paired control group of 14 healthy controls and 14 participants with post-traumatic stress disorder. Individuals with DID and simulators participated in a trauma-avoidant and a trauma-related identity state. Reaction times and brain activation following overtly and covertly presented individualised words were statistically analysed.

**Results:**

Behavioural analyses showed a main effect of consciousness (*P* < 0.001). Post hoc between-group pairwise comparisons revealed slower reaction times for individuals with DID compared with simulating (*P* < 0.05) and paired controls (*P* < 0.05). Neural data analyses showed increased brain activation in frontal and parietal regions within the diagnosed DID group, especially during overt processing. Between-group comparisons mostly showed less pronounced activation in frontal, occipital and temporal areas.

**Conclusions:**

The present study showed increased cognitive control during overt self-relevant knowledge processing in the trauma-avoidant identity state of DID, in line with previous research. The slower reaction times and increased frontoparietal activation shown in individuals with diagnosed DID, as compared with both control groups, support the notion of cognitive avoidance of trauma-related information in DID and further reinforce the authenticity of DID experiences.

Dissociative identity disorder (DID) is a complex psychiatric disorder characterised by the recurrent presentation of two or more distinct dissociative identity states.^1^ These are referred to as trauma-avoidant identity state and trauma-related identity state.^2–4^ The trauma-avoidant identity state of individuals with DID mentally avoids trauma-related knowledge and often reports subjective amnesia.^1,2,5,6^ In a trauma-related identity state, individuals with DID are more likely to recall autobiographical trauma-related experiences.^1,3,7–9^ Most research in DID has focused on overt stimulus presentation, while investigation of both overt and covert processing has mainly focused on emotional face-processing^10,11^ or mirror confrontation tasks.^12,13^ The majority of these studies used standardised measures rather than subject-specific trauma-related stimuli. The present study aims to expand the literature through overt and covert presentation of individualised words.

Studying overt and covert processing using words is particularly advantageous because it allows for a highly individualised approach. Words can be carefully selected and individualised to each participant, ensuring that the stimuli are personally relevant and engaging. Additionally, words as stimuli provide a precise and measurable way to assess cognitive and emotional processing, which is particularly helpful in studying complex phenomena like dissociation where personalised and context-specific triggers can reveal nuanced insights into overt and covert processing mechanisms. For these reasons, in the present study individualised words were used. During the overt task condition the participants decided whether the word was relevant to themselves (as in^2^), while during the covert condition they decided whether or not the word contained a capital letter. This covert presentation method was previously proven effective in the research of Daselaar and colleagues^14^ in healthy controls. The inclusion of a capital letter in a trauma-related word created a conflict of task, because the emotional content of the word was interfering with the decision process whether or not the word contained a capital letter, leading to a slower response to the actual capital letter task.^14^

## Aims and hypotheses

The present study aimed to investigate differences in behavioural performance and brain activation following overt and covert processing of self-relevant words, as compared with non-self-relevant words, in individuals with a diagnosis of DID and in a controlled manner. Therefore, we included three individualised word types, namely self-relevant trauma-related, non-self-relevant trauma-related (NSt) and non-self-relevant neutral (NSn) words. These were rated for self-relevance (yes/no) during the overt task condition and for the presence of a capital letter during the covert task condition (yes/no), while neural and behavioural data were collected. Behavioural performance and brain activation of participants with DID were compared with two control group:; DID-simulating actors and a carefully paired control group consisting of individuals with post-traumatic stress disorder (PTSD) and healthy participants.

With regard to the behavioural data, we hypothesised that participants with DID would demonstrate slower reaction times to covertly presented self-relevant and trauma-related stimuli when in the trauma-related identity state, as compared with the control groups. Regarding the neural data, we hypothesised that individuals with DID would exhibit increased brain activation in the frontal and parietal regions when in the trauma-avoidant identity state, and in the areas of amygdala and insula when in the trauma-related identity state. Lastly, we hypothesised that participants with DID would show increased brain activation during covert processing, as compared with overt processing, especially in frontal regions.

## Method

### Participants

The present study was part of the larger Dutch Neuroimaging Dissociative Identity Disorder project, originally conducted in The Netherlands (see, for example,^2,9,15–19^), where participants underwent task-based functional magnetic resonance imaging (fMRI) during self-relevance rating of words from individualised word lists. Ethical approval was acquired by the Amsterdam Medical Centre (reference no. MEC09/155) and the Medical Ethical Committee of the University Medical Centre Groningen (reference no. METC2008.211).

Behavioural and neural data of 56 participants were included in the current study: 14 women with a diagnosis of DID (DID-G), 14 healthy DID-simulating actors (DID-S) and a paired between-subject control (CTRL) group consisting of a total of 28 participants – 14 healthy non-psychiatric controls and 14 individuals with a PTSD diagnosis. The individuals with PTSD were included as controls for the trauma-related identity state of the DID-G participants,^9^ whereas healthy, non-simulating, study-blind participants were included as a control group for the trauma-avoidant identity state of the DID-G group. The PTSD participants had a history of interpersonal traumatising events during childhood and/or adulthood, and they scored significantly lower than diagnosed DID individuals in dissociation scales,^9,16,17,19^ thus not meeting the criteria for the dissociative subtype of PTSD. The healthy controls were, by default, non-psychiatric individuals due to our inclusion criteria that required, among others, low scores on the Dissociative Experiences Scale (cut-off <25), the Somatoform Dissociation Questionnaire (SDQ-20, cut-off <28; SDQ-5, cut-off <7), the Traumatic Experience Checklist (impact <2) and the State-Trait Anxiety Inventory-Trait scale, as well as no history of psychiatric disorders or psychiatric medication.^9,15,16,20,21^ In line with the design and terminology used in our previous publications, in this study we refer to these non-psychiatric individuals as healthy controls. All participants were carefully matched in terms of gender, age and education level, were of Western European ancestry, native speakers of Dutch and aged between 18 and 65 years. Before their participation, individuals were informed about, and provided written consent to, the procedures, their withdrawal rights and the anonymity and confidentiality of their data. Further information regarding the specific characteristics of each participant group and reported identity states, simulation instructions and inclusion and exclusion criteria,^9,16,18,19^ as well as comorbid disorders,^20,21^ has previously been detailed.

### Materials and procedure

#### Word lists

**Fig. 1:**
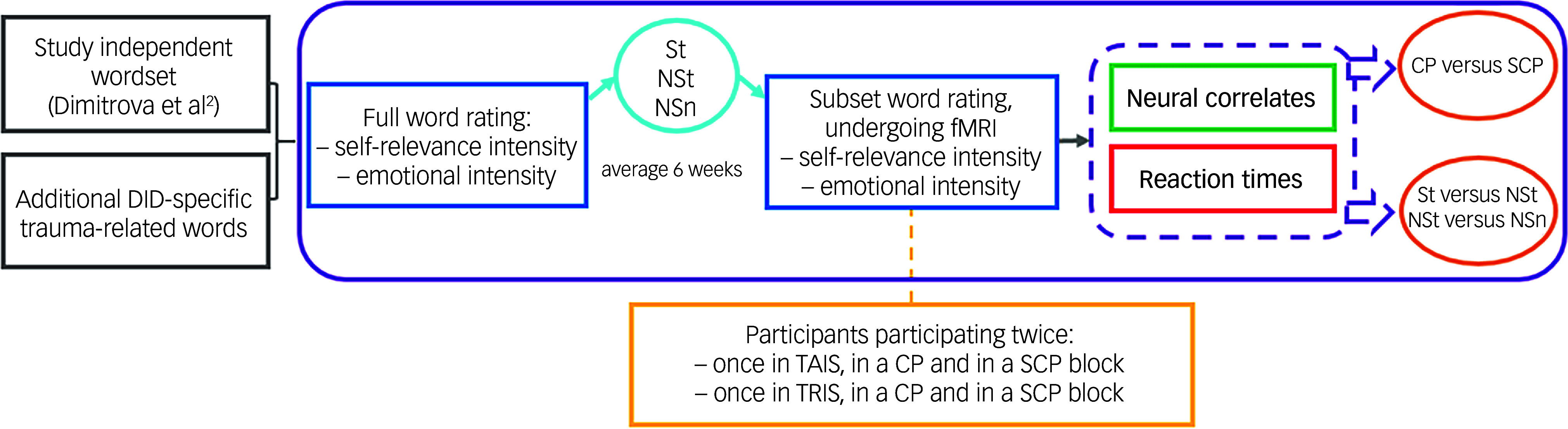
Study procedure. DID, dissociative identity disorder; St, self-relevant trauma-related; NSt, non-self-relevant trauma-related; NSn, non-self-relevant neutral; fMRI, functional magnetic resonance imaging; CP, conscious/overt processing; SCP, subconscious/covert processing; TAIS, trauma-avoidant identity state; TRIS, trauma-related identity state.

Individualised, subject-specific words were used during the behavioural data and fMRI sessions of the present study. Details on acquiring and determining these subject specific words are provided in Appendix A of the supplementary materials available at https://doi.org/10.1192/bjo.2025.10914, reprinted from Strouza and colleagues^17^ (also used by Dimitrova and colleagues^2^). The word selection procedure is visualised in Fig. 1 (adapted with permission from Dimitrova and colleagues.^2^

#### Brain imaging task description

**Fig. 2:**
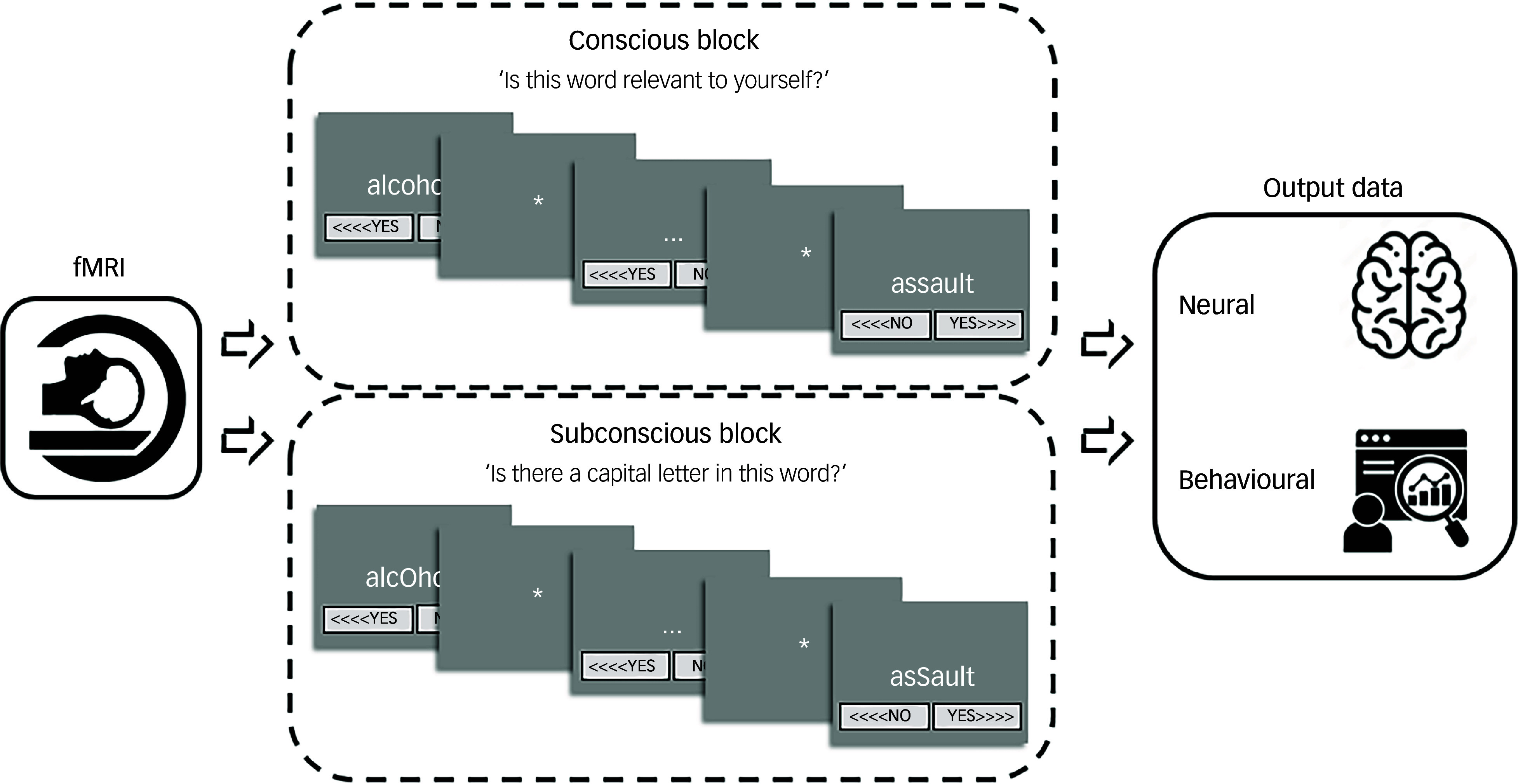
Brain-imaging session procedure of self-relevance processing. Each task contained a total of five conscious/overt processing (CP) blocks and five subconscious/covert processing (SCP) blocks. The SCP block consisted of the same words included in the CP block but contained capital letters (i.e. asSault). The participants’ task was to decide whether or not there is a capital letter, instead of rating the word in terms of self-relevance and emotional content. There were two different versions of CP/SCP block order to allow for between subject randomisation, randomly allocated one for the trauma-related identity state and one for the trauma-avoidant identity state. Every block contained the self-relevant trauma-related, non-self-relevant trauma-related and non-self-relevant neutral conditions in randomised order within block, i.e. four words from each condition randomly distributed in each block. The reply button allocation was also randomised, sometimes (<>>) and sometimes (<<>) and counterbalanced throughout the task. The word for rating would appear in the centre of the screen for up to 5 s or until the Presentation program logged a response, i.e self-paced termination of the word presentation. Although the task was designed to run at the pace of the participant, the maximum duration of each block was up to 25 min. fMRI, functional magnetic resonance imaging.

The imaging task was implemented and programmed by S.C. in the software programme Presentation (version 14 for Windows, Neurobehavioral Systems, San Francisco, California, USA; https://neurobs.com/menu_presentation/menu_features/features_overview). This programme was also used to record the participants’ reactions to each word during the brain imaging session. Choice reaction time is a basic measure of processing speed, and constitutes a calculation of the participant’s response time from stimulus presentation to logged response.^22^ Slower reaction times indicated greater time between the presentation of the stimulus and the participant’s response, denoting increased mental processing, whereas faster reaction times signified less time taken by the participant to respond, suggesting decreased mental processing and cognitive avoidance.^19^ The fMRI task procedure is visualised and detailed in Fig. 2 (adapted with permission from Dimitrova and colleagues^2^).

### Data analyses

#### Behavioural data

Presentation log files were extracted, processed and transformed by A.J.L. using R (version 3.4.1 for Windows, R Core Team, Vienna, Austria; https://cran.r-project.org/). IBM SPSS Statistics software (version 24 for Windows, IBM, Armonk, New York, USA; https://www.ibm.com/products/spss-statistics) was then used for descriptive and statistical analyses. Participants’ reaction times were analysed using repeated-measures analyses of variance. A four-way factorial design was employed to explore the main effect of consciousness, with participant group being the between-subjects factor (DID-G, DID-S, CTRL). The remaining variables were all within-subject factors: two dissociative identity states (trauma-avoidant identity state, trauma-related identity state), two trial types (conscious/overt processing, subconscious/covert processing (SCP)) and three word types (non-self-relevant neutral (NSn), non-self-relevant trauma-related (NSt), self-relevant trauma-related). Regarding the self-relevance and emotional intensity effect, the same design was employed but, rather than three word types there were only two; for the self-relevance effect we compared between self-relevant and non-self-relevant words (self-relevant trauma-related versus NSt), whereas for the emotional intensity effect we contrasted between trauma-related and neutral words (NSt versus NSn). Simple contrast analyses were conducted to determine the one-tailed direction of the tests, serving as the primary analytical results for the specified hypotheses. Post hoc pairwise comparisons tests were used to determine the directionality of significant differences. *P*-values were set at 0.05 and corrected for multiple comparisons through Bonferroni’s adjustment. The same factorial designs were also employed for within-group comparisons.

#### Neuroimaging data

An in-depth overview of the acquisition parameters and preprocessing details has previously been described in detail, in the supplementary materials of Dimitrova and colleagues.^2^ FMRI data were processed and statistically analysed using SPM12 (version 7771 for Windows, Wellcome Trust Centre for Neuroimaging, London, UK; http://www.fil.ion.ucl.ac.uk) within Matlab (version 9.2.0 for Windows, MathWorks, Natick, Massachusetts, USA; https://www.mathworks.com/products/matlab.html).

For the purposes of statistical analyses, a blocked event-related design was employed. At the first level, a boxcar regressor, aligned with the onset and offset of stimulus presentation, underwent convolution with the canonical haemodynamic response function. At this level, 9 regressors were specified for each session and each identity state plus 4 session-specific constants, totalling 40 regressors per participant. Of the nine regressors there was one low-level baseline condition of no interest, four regressors modelled subconscious/covert processing and four regressors modelled conscious/overt processing. According to processing level, three regressors modelled our conditions of interest, namely the word types NSn, NSt and self-relevant trauma-related, and one regressor, which was of no interest in regard to the current study. Subsequently, image volumes containing parameter estimates pertinent to the current investigation were computed within the self-relevance task blocks and consciousness block manipulation, and taken to the second level of analysis.

At the second level, contrasts of interest were investigated using within-group, one-sample *t*-tests (for the DID-G group) and between-group, two-sample *t*-tests to explore the effects of group (DID-G versus CTRL, DID-G versus DID-S). The main effect of consciousness tested participants’ between-identity state increased and decreased brain activation in the overt versus covert level of processing. The effect of self-relevance (self-relevant trauma-related versus NSt) and that of emotional intensity (NSt versus NSn) were investigated using the following comparisons of interest: consciousness effects within identity states (covert versus overt processing in the trauma-avoidant identity state, covert versus overt processing in the trauma-related identity state) and consciousness effects between identity states (overt processing in the trauma-related versus trauma-avoidant identity state, covert processing in the trauma-related versus trauma-avoidant identity state).

An initial threshold of *P* < 0.05, corrected with family-wise error multiple comparisons for the whole brain, was set with an extent threshold of 8 voxels. Furthermore, brain regions meeting an explorative threshold of *P* < 0.005 uncorrected were also reported, consistent with previous studies.^2,3,8,23^ Results were restricted by an extent voxel threshold of ≥8, in line with the spatial resolution of the data, to mitigate the risk of type I error following the approach outlined by Bennett and colleagues.^24^ Coordinates were extracted from SPM12 with relevant output and label. The labels of the coordinates were cross-referenced by A.I.S. and A.A.T.S.R., using the atlas Siibra Explorer (browser-based, EBRAINS, Watermael-Boitsfort, Belgium; https://atlases.ebrains.eu/viewer/) and the application Brain Tutor 3D (Windows, BrainVoyager, Maastricht, The Netherlands; https://www.brainvoyager.com/products/braintutor.html). The coordinates and labels were further examined in SPM using anatomical visualisation of clusters by means of overlaying a canonical, single-subject T1 image on the sagittal, coronal and axial sections.

## Results

### Demographics

Participants did not significantly differ in terms of age or education.

### Behavioural and brain imaging data

#### Main effect of consciousness

**Fig. 3:**
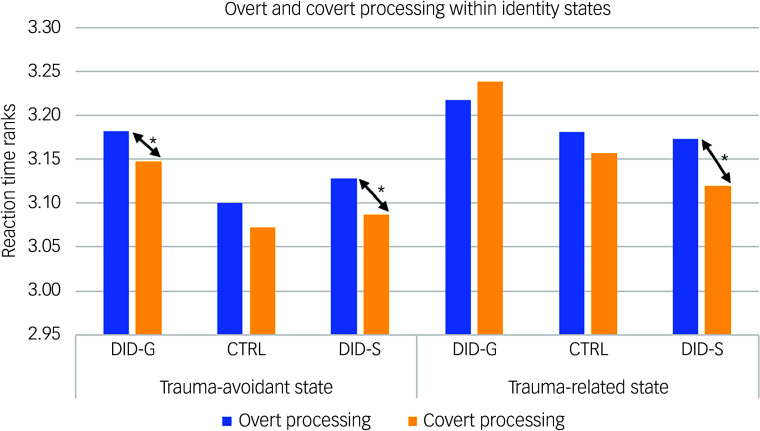
Mean participant score ranks of response times during overt and covert processing, within identity states. DID-G, genuine/diagnosed dissociative identity disorder; CTRL, paired control group; DID-S, simulated dissociative identity disorder. *0.001 < *P* < 0.05.

Behavioural data showed a significant main effect of consciousness in participants’ reaction times for self-relevant trauma-related, NSt and NSn words across groups and identity states (see Table 1). Pairwise comparisons revealed slower reaction times in conscious/overt processing compared with subconscious/covert processing (see Table 1, main consciousness effect), especially in the trauma-avoidant identity state for the DID-G group, and in both identity states for DID-S participants (see Fig. 3).

**Table 1:** Statistical comparisons of behavioural response times between groups, processing blocks and dissociative identity states

	ANOVA			Pairwise comparison		
Effects recorded	*F*(d.f.)	Sig.	*η*_*p*_^2^	Directionality	Mean difference	Sig.
**Main consciousness effect**						
CP versus SCP	23.096 (1, 39)	<0.001	0.372	CP > SCP	0.034	<0.001
**Self-relevance effect (St versus NSt)**						
*Within-group comparisons*						
DID-G	0.180 (1)	n.s.	0.014			
CP versus SCP	0.212 (1)	n.s.	0.016			
TRIS versus TAIS	3.364 (1)	n.s.	0.206			
DID-S	1.905 (1)	n.s.	0.128			
CP versus SCP	8.579 (1)	<.05	0.398	CP > SCP	0.048	<0.05
TRIS versus TAIS	3.456 (1)	n.s.	0.210			
CTRL	16.681 (1)	<0.05	0.562		0.022	<0.05
CP versus SCP	20.230 (1)	<0.001	0.609	CP > SCP	0.026	<0.001
TRIS versus TAIS	10.025 (1)	<0.05	0.435	TRIS > TAIS	0.082	<0.05
*Between-group comparisons*						
CP versus SCP	12.606 (1)	<0.05	0.244	CP > SCP	0.027	<0.05
TRIS versus TAIS	14.778 (1)	<0.001	0.275	TRIS > TAIS	0.062	<0.001
DID-G versus DID-S				DID-G > DID-S	0.069	<0.05
DID-G versus CTRL				DID-G > CTRL	0.069	<0.05
**Emotional intensity effect (NSt versus NSn)**						
*Within-group comparisons*						
DID-G	0.281 (1)	n.s.	0.021			
CP versus SCP	3.462 (1)	n.s.	0.210			
TRIS versus TAIS	4.053 (1)	n.s.	0.238			
DID-S	4.089 (1)	n.s.	0.239			
CP versus > SCP	7.929 (1)	<.05	0.379	CP > SCP	0.041	<0.05
TRIS versus TAIS	2.328 (1)	n.s.	0.152			
CTRL	1.772 (1)	n.s.	0.120			
CP versus SCP	31.562 (1)	<0.001	0.708	CP > SCP	0.038	<0.001
TRIS versus TAIS	7.718 (1)	<0.05	0.373	TRIS > TAIS	0.071	<0.05
*Between-group comparisons*						
CP versus SCP	24.790 (1)	<0.001	0.389	CP > SCP	0.035	<0.001
TRIS versus TAIS	13.095 (1)	<0.001	0.251	TRIS > TAIS	0.056	<0.001
DID-G versus DID-S				DID-G > DID-S	0.080	<0.05
DID-G versus CTRL				DID-G > CTRL	0.076	<0.05

ANOVA, analysis of variance; Sig., significance; CP, conscious/overt processing; SCP, subconscious/covert processing; St, self-relevant trauma-related words; NSt, non-self-relevant trauma-related words; DID-G, diagnosed dissociative identity disorder participants; n.s., no significance; TRIS, trauma-related identity state; TAIS, trauma-avoidant identity state; DID-S, simulated dissociative identity disorder participants; CTRL, paired control group; NSn, non-self-relevant neutral words.

**Fig. 4:**
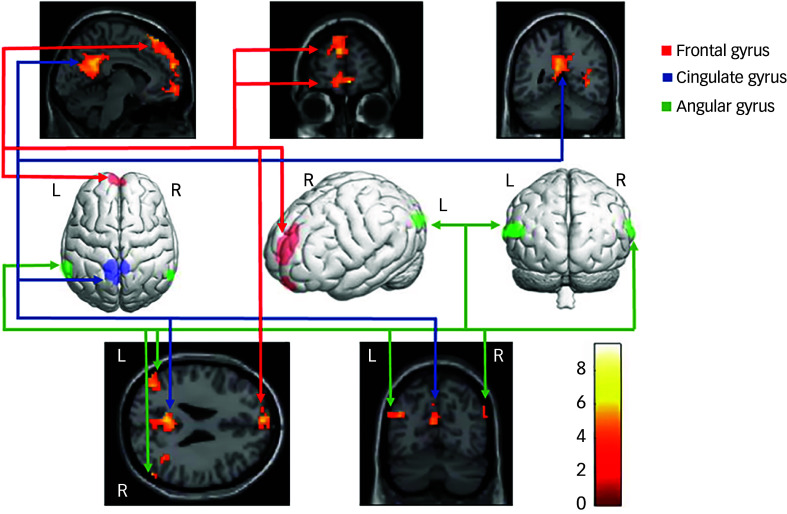
Neural correlates of the main effect of consciousness in dissociative identity disorder. L/R, Left/right side of the brain.

Neural data for the diagnosed DID-G participants showed increased brain activation in the bilateral frontal gyrus, bilateral intraparietal sulcus, bilateral angular gyrus, right lingual gyrus, right temporal gyrus and left cingulate gyrus (see Table 2, Fig. 4 and Appendix B of the supplementary materials). Group comparisons between the DID-G and paired control group (CTRL), as well as between the DID-G and DID-S groups, showed increased brain activation in the bilateral occipital gyrus and bilateral intraparietal sulcus, respectively. Decreased brain activation was found for the DID-G group in the left middle frontal gyrus, right precentral gyrus, bilateral intraparietal sulcus and bilateral superior and inferior parietal lobules, as well as in the right precuneus.

**Table 2:** Main effect of consciousness: increased and decreased brain activation

			Within-group					Between-group									
			DID-G					DID-G versus CTRL					DID-G versus DID-S				
L/R	Brain region	BA	*X*	*Y*	*Z*	T*P	*kE*^c	*X*	*Y*	*Z*	T^*P	*kE*^c	*X*	*Y*	*Z*	T^*P	*kE*^c
**Overt versus covert processing**																	
**Increased brain activation**																	
*Cortical areas*																	
L	Superior frontal gyrus	BA 8/9	−26	30	50	6.76	337^**c^1										
		BA 8	−8	54	36	6.13	337^**c^2										
		BA 8	−1	30	61	6.06	337^**c^3										
R	Medial frontal gyrus	BA 10	6	57	−9	6.84	82^**c^1										
L	Medial frontal gyrus	BA 10	−4	61	−6	5.17	82^**c^2										
		BA 10	−12	44	−6	3.72^*P*	82^**c^3										
L	Middle frontal gyrus	BA 8	−40	16	50	5.78	31^1										
		BA 6	−36	5	61	4.08^*P*	31^2										
L	Inferior frontal gyrus	BA 45/47	−46	33	−9	6.37	91^**c^1										
		BA 45/47	−40	23	8	5.52	91^**c^2										
		BA 45/47	−36	30	−2	5.06	91^**c^3										
L	Precentral gyrus	BA 6						−36	−12	40	3.93	14					
R	Precentral gyrus	BA 4/6						66	−5	12	3.69^*P*	15^1	20	−8	54	3.40^*P*	8
		BA 4/6						58	2	8	3.01^*P*	15^2					
L	Intraparietal sulcus	BA 7/40						−43	−33	50	3.84	16					
R	Intraparietal sulcus	BA 7/40											30	−43	40	3.53^*P*	9
L	Angular gyrus	BA 39	−43	−74	30	9.56^**P	54^1										
		BA 39	−43	−74	44	3.51^*P*	54^2										
R	Angular gyrus	BA 39	55	−71	30	6.42	9										
		BA 39	38	−57	26	4.65	36^2										
R	Lingual gyrus	BA 18/19	34	−53	2	6.52	36^1										
L	Middle occipital gyrus	BA 19						−40	−84	5	3.68^*P*	19					
R	Middle occipital gyrus	BA 19						41	−71	2	3.67^*P*	13					
		BA 18											34	−84	2	3.87	8
R	Occipital gyrus	BA 18						24	−91	2	4.40	141^**c^1					
		BA 18						10	−95	26	4.24	141^**c^2					
		BA 18						20	−98	5	3.98	141^**c^3					
L	Occipital gyrus	BA 18						−12	−102	12	3.88	17					
L	Occipital gyrus/calcarine sulcus	BA 17/18						−15	−102	−2	3.56^*P*	8					
L	Calcarine sulcus	BA 17						−22	−67	19	3.73^*P*	8					
R	Superior temporal gyrus	BA 22	38	−46	16	4.62	36^3	38	−46	16	3.17^*P*	9^1					
		BA 22						48	−50	8	3.17^*P*	9^2	55	−57	−2	3.46^*P*	16
R	Middle temporal gyrus	BA 21	62	−5	−16	5.02	8										
R	Precuneus	BA 7						13	−46	54	3.90	12					
L	Cingulate sulcus	BA 23/31	−4	−53	26	6.00	282^**c^2										
R	Cingulate sulcus	BA 23/31						13	−39	33	3.78^*P*	8					
L	Cingulate gyrus	BA 31	−1	−46	33	6.19	282^**c^1	−15	−36	30	3.32^*P*	10^2					
		BA 31	−4	−64	19	5.07	282^**c^3	−18	−39	40	3.41^*P*	10^1	−22	−33	40	3.88	11
*Subcortical areas*																	
R	Caudate nucleus (latero-dorsal part)							30	9	19	4.60	19					
**Decreased brain activation**																	
*Cortical areas*																	
L	Middle frontal gyrus	BA 9	−26	44	26	4.80	11^1										
		BA 9	−29	37	30	4.49^*P*	11^2										
R	Precentral gyrus	BA 6	48	2	36	4.37^*P*	12										
R	Intraparietal sulcus	BA 7/40	41	−43	44	4.35	66^**c^1										
L	Intraparietal sulcus	BA 7/40	−40	−46	36	3.94^*P*	21^1										
		BA 7/40	−43	−46	47	3.73^*P*	21^2										
L	Superior parietal lobule	BA 7	−18	−74	36	4.18^*P*	31^1										
		BA 7	−29	−64	50	3.92^*P*	31^2										
R	Superior parietal lobule	BA 7	16	−74	40	4.62	31^1										
		BA 7	24	−71	36	4.17^*P*	31^2										
L	Inferior parietal lobule	BA 40	−64	−46	33	5.18	89^**c^1										
		BA 40	−64	−26	44	5.17	89^**c^2										
		BA 40	−60	−26	30	4.89	89^**c^3										
R	Inferior parietal lobule	BA 40	58	−43	40	4.29	66^**c^2										
		BA 40	66	−26	40	4.10	66^**c^3										
L	Inferior occipital gyrus	BA 18	−43	−88	−2	4.41^*P*	8										
R	Calcarine sulcus	BA 17	30	−67	22	3.68^*P*	8^2										
L	Middle temporal gyrus	BA 21	−46	−1	−12	5.24	9^1										
		BA 21	−40	−12	−9	4.53^*P*	9^2										
R	Precuneus	BA 31	24	−60	19	4.03^*P*	8^1										
L	Cingulate gyrus	BA 32	−4	9	44	4.52^*P*	21^1										
		BA 32	−12	2	47	4.41^*P*	21^2										
L	Fusiform gyrus	BA 37	−43	−60	2	6.41	38^1										
		BA 37	−46	−50	−12	3.58^*P*	38^2										
R	Fusiform gyrus	BA 37	58	−53	−6	6.09	14										
*Subcortical areas*																	
L	Caudate nucleus (tail)												−32	−33	16	3.36^*P*	8
R	Putamen		6	−29	26	5.69	19										
R	Thalamus		13	−19	2	5.78	9										

DID-G, genuine/diagnosed dissociative identity disorder; CTRL, paired control group; DID-S, simulated dissociative identity disorder; BA, Brodmann area; *X*, *Y*, *Z*, Montreal Neurological Institute coordinates (mm); L/R, left/right side of the brain; *T*, *t*-value statistic; *kE*, cluster size in voxels (one voxel is 26 262 mm).

^1, first peak voxel; ^2, second peak voxel; ^3, third peak voxel; ^**c, 0.05 corrected for multiple comparisons at cluster level; ^c, cluster size obtained *P* = 0.005 uncorrected; ^**, *P* = 0.05 corrected for multiple comparisons at peak level; ^*P, also at 0.001 uncorrected for multiple comparisons at peak level; ^*P*, only at 0.005 uncorrected for multiple comparisons at peak level.

### Self-relevance effect: self-relevant trauma-related versus NSt words

#### Within-group comparisons

Behavioural data showed significant differences in reaction times only for the CTRL group (see Table 1). Post hoc pairwise comparisons showed slower reaction times for self-relevant trauma-related words compared with NSt words. Significant differences between overt and covert processing were found only for DID-S and CTRL participants. Regarding rating differences between the trauma-avoidant identity and -related identity states, only the CTRL group yielded significant results, with slower reaction times for the latter compared with the former (see Table 1).

Neural data obtained during the brain imaging session showed increased brain activation in the left superior frontal gyrus and right superior parietal lobule in the trauma-related identity state of diagnosed DID-G participants (see Table 3). In the covert processing block, there was increase in the left hippocampal gyrus and left superior temporal gyrus. Decreased activation was found in the right dorsolateral prefrontal cortex, right postcentral gyrus and left precentral gyrus while participants were in the trauma-avoidant identity state. Additionally, there was decreased activation in the right superior frontal gyrus, right middle frontal gyrus, left inferior frontal gyrus and right inferior parietal lobule while in the trauma-related identity state, and in the left superior frontal and left middle frontal gyrus during the covert processing block (see Table 3 and Appendix B of the supplementary materials).

**Table 3:** Self-relevance effect (St versus NSt): increased and decreased brain activation

L/R	Brain region	BA	Within-group					Between-group									
			DID-G					DID-G versus CTRL					DID-G versus DID-S				
			*X*	*Y*	*Z*	*T*^*P	*kE*^c	*X*	*Y*	*Z*	T^*P	*kE*^c	*X*	*Y*	*Z*	T^*P	*kE*^c
**Self-relevance effect (St versus NSt)**																	
**Increased brain activation between processing levels**																	
***TAIS***																	
*Cortical areas*																	
L	Inferior frontal gyrus	BA 45											−57	19	12	3.72^*P*	45^1^c
		BA 45											−46	16	2	3.54^*P*	45^2^c
		BA 44/45											−22	26	16	3.51^*P*	12
L	Precentral gyrus	BA 4						−57	−5	12	3.47^*P*	10					
R	Angular gyrus	BA 39	55	−60	40	3.99^*P*	14^1										
		BA 39	55	−60	26	3.82^*P*	14^2						58	−60	26	3.28^*P*	8
R	Cingulate gyrus	BA 24											10	−12	33	4.59	16^1
		BA 24											13	−22	36	2.90^*P*	16^2
R	Insula	BA 13						41	−12	12	3.38^*P*	9					
L	Superior parietal lobule	BA 5											−15	−33	44	4.10	8
		BA 7											−18	−64	40	3.73^*P*	39^c
L	Inferior parietal lobule/angular gyrus	BA 39						−60	−46	22	4.71	18^1					
		BA 39						−50	−43	22	3.69^*P*	18^2					
R	Lingual gyrus	BA 18											10	−71	2	4.05	14
L	Middle temporal gyrus	BA 21						−60	−64	8	4.96	8					
L	Transverse temporal gyrus	BA 41						−36	−39	8	4.62	11					
*Subcortical areas*																	
R	Caudate nucleus (dorsal part)		24	−33	22	4.00^*P*	9										
***TRIS***																	
*Cortical areas*																	
L	Superior frontal gyrus/frontal operculum	BA 6	−57	9	−2	4.03^*P*	8	41	−1	36	3.35^*P*	9					
R	Superior parietal lobule	BA 7	2	−74	58	4.46^*P*	15										
R	Angular gyrus	BA 39	48	−46	19	4.14^*P*	9										
L	Middle temporal gyrus	BA 21	−64	−46	5	4.69	9										
R	Middle cingulate gyrus	BA 24	2	−12	36	4.48^*P*	15^1	−4	−15	44	4.53	25^c					
L	Cingulate gyrus	BA 24	−12	−12	33	3.92^*P*	15^2										
		BA 30	−18	−46	2	4.35^*P*	16										
*Subcortical areas*																	
	n.s.																
**Increased brain activation between identity states**																	
***Overt processing***																	
*Cortical areas*																	
L	Inferior frontal gyrus	BA 44											−60	16	16	3.94	14
		BA 44											−43	5	12	3.75^*P*	8
R	Precentral gyrus	BA 4/6											30	−5	33	3.92	13
R	Angular gyrus	BA 39											52	−60	30	4.05	22
R	Tranverse temporal gyrus/Heschl gyrus	BA 41											38	−33	8	3.63^*P*	15^1
L	Precuneus	BA 31											−8	−53	26	3.60^*P*	10
R	Cingulate gyrus	BA 32	24	40	8	3.52^*P*	9^2						10	33	16	4.21	40^2^c
*Subcortical areas*																	
R	Caudate nucleus (dorsal part)		16	26	16	4.03^*P*	9^1						20	33	12	4.72	40^1^c
													24	23	22	4.00	40^3^c
R	Thalamus												27	−26	8	3.03^*P*	15^2
													20	−29	12	3.01^*P*	15^3
***Covert processing***																	
*Cortical areas*																	
L	Hippocampal gyrus	BA 30	−22	−39	8	4.39^*P*	10										
L	Superior temporal gyrus	BA 22	−50	5	−6	4.97	8										
L	Fusiform gyrus	BA 37/17						−32	−53	−2	3.37^*P*	8					
*Subcortical areas*																	
L	Caudate nucleus (dorsal part)							−22	9	22	3.68^*P*	8					
**Decreased brain activation between processing levels**																	
***TAIS***																	
*Cortical areas*																	
R	Superior frontal gyrus	BA 8						13	44	44	5.00	113^1^**c					
L	Superior frontal gyrus	BA 8						−8	26	44	4.45	113^2^**c					
		BA 8						−8	19	50	4.02	113^3^**c					
R	Dorsolateral prefrontal cortex	BA 9	16	44	40	4.81	12										
R	Postcentral gyrus	BA 1/3	52	−15	36	3.98^*P*	14										
L	Precentral gyrus	BA 4	−43	−12	26	4.72	10										
*Subcortical areas*																	
	n.s.																
***TRIS***																	
*Cortical areas*																	
R	Superior frontal gyrus	BA 6/8	16	19	61	4.65	8										
R	Middle frontal gyrus	BA 8	30	23	54	5.40	8										
L	Middle frontal gyrus	BA 10											−40	44	22	3.72^*P*	9
L	Inferior frontal gyrus	BA 44	−40	12	19	5.08	12						−40	12	19	3.43^*P*	26^2
		BA 44											−43	5	16	3.63^*P*	26^1
L	Inferior frontal sulcus												−32	9	22	3.29^*P*	26^3
R	Inferior parietal lobule/angular gyrus	BA 39	52	−71	33	4.50^*P*	8										
R	Parahippocampal gyrus/frontal operculum	BA 30											30	−33	5	3.38^*P*	9
L	Cingulate gyrus	BA 23											−4	−53	30	4.16	17
*Subcortical areas*																	
R	Putamen												30	−15	−2	3.61^*P*	14^1
													27	−8	2	3.47^*P*	14^2
**Decreased brain activation between identity states**																	
***Overt processing***																	
*Cortical areas*																	
R	Middle frontal gyrus	BA 9						48	26	40	3.24^*P*	9					
L	Lingual gyrus	BA 19	−15	−53	−2	4.39^*P*	12										
L	Inferior temporal gyrus	BA 37											−46	−64	−2	3.06^*P*	8^2
L	Fusiform gyrus	BA 37											−50	−53	−6	3.84	8^1
L	Calcarine sulcus	BA 17						−26	−81	2	3.79^*P*	11					
*Subcortical areas*																	
	n.s.																
***Covert processing***																	
*Cortical areas*																	
L	Superior frontal gyrus	BA 8	2	26	50	4.73	14^1										
		BA 8/6	10	19	47	4.14^*P*	14^2										
L	Middle frontal gyrus	BA 8	−50	16	40	4.93	13										
L	Inferior frontal gyrus	BA 44											−43	12	5	5.67	32^c
L	Superior parietal lobule/precuneus	BA 7											−12	−53	61	3.54	15
L	Calcarine sulcus	BA 17	−22	−71	8	5.28	8	−22	−71	8	5.08	15	−18	−74	8	3.89	9
*Subcortical areas*																	
L	Brain stem												−1	−26	−9	4.51	12

L/R, left/right side of the brain; BA, Brodmann area; DID-G, genuine/diagnosed dissociative identity disorder; CTRL, paired control group; DID-S, dissociative identity disorder-simulating controls; *kE*, cluster size in voxels (one voxel is 26 262 mm); (*X*, *Y*, *Z*), Montreal Neurological Institute coordinates (mm); *T*, *t*-value statistic; St, self-relevant trauma-related stimuli; NSt, non-self-relevant trauma-related stimuli; TAIS, trauma-aware identity state; TRIS, trauma-related identity state; n.s., no significance.

^1, first peak voxel; ^2, second peak voxel; ^3, third peak voxel. ^**c, 0.05 corrected for multiple comparisons at cluster level; ^c, cluster size obtained *P* = 0.005 uncorrected; ^**, *P* = 0.05 corrected for multiple comparisons at peak level; ^*P, also at 0.001 uncorrected for multiple comparisons at peak level; ^*P*, only at 0.005 uncorrected for multiple comparisons at peak level.

#### Between-group comparisons

Behavioural data showed significant between-group effects (*F*(2, 39) = 4.829, *P* = 0.013, *η*_*p*_^2^ = 0.199). Statistically different reaction times were shown between the two word types (*F*(1) = 9.216, *P* = 0.004, *η*_*p*_^2^ = 0.191), with pairwise comparisons revealing slower reaction times for self-relevant trauma-related words compared with NSt words (mean difference = 0.011, s.e. = 0.004, 95% CI: [0.004, 0.019], *P* = 0.004) across groups. Significant differences were observed between overt and covert processing, as well as between trauma-avoidant and -related identity states (see Table 1). Post hoc pairwise comparisons between groups showed slower reaction times in overt processing and in the trauma-related identity state. Pairwise comparisons also showed that DID-G participants exhibited slower reaction times than the CTRL and DID-S groups.

Neural data of group comparisons between the DID-G and CTRL groups showed increased brain activation in the left precentral gyrus, right insula, left inferior parietal lobule/angular gyrus, left middle temporal gyrus and left transverse temporal gyrus in the trauma-avoidant identity state, and in the left superior frontal gyrus in the trauma-related identity state. Decreased activation was observed in the bilateral superior frontal gyrus in the trauma-avoidant identity state, and in the right middle frontal gyrus during the overt processing block (see Table 3).

Comparisons between the DID-G and DID-S groups showed increased activation in the left inferior frontal gyrus, left superior and inferior parietal lobule, mainly in the trauma-avoidant identity state. Increased activation was found in the left inferior frontal gyrus, right precentral gyrus, right transverse temporal gyrus and left precuneus only in the overt processing block. Decreased activation was found in the trauma-related identity state in the left middle and inferior frontal gyrus and sulcus, as well as in the right parahippocampal gyrus/frontal operculum. Decreased activation was observed in the left inferior temporal gyrus in the overt processing block, and in the left inferior frontal gyrus, left superior parietal lobule/precuneus and left calcarine sulcus in the covert processing block (see Table 3 and Appendix B of the supplementary materials).

### Emotional intensity effect: NSt versus NSn words

#### Within-group comparisons

Behavioural analyses revealed no within-group differences between these two word types (see Table 1). Significant differences between overt and covert processing were found for the DID-S and CTRL groups. Comparisons between the trauma-avoidant and -related identity states showed differences only for the CTRL group, with slower reaction times in the latter.

Post hoc emotional intensity assessment of the imaging data showed increased activation in the bilateral superior frontal gyrus, left middle frontal gyrus, bilateral precentral and postcentral gyrus, left anterior insula, left superior parietal lobule, left supramarginal gyrus, right superior and left middle/lateral occipital gyrus, right cuneus and left calcarine sulcus, but only in the trauma-related identity state (see supplementary Table 1). Increased activation in the bilateral superior frontal gyrus, left middle and inferior frontal gyrus, bilateral precentral gyrus, right postcentral gyrus, left anterior insula, bilateral superior parietal lobule, bilateral precuneus, right lateral and left occipital gyrus and bilateral calcarine sulcus was observed in the covert processing block. Decreased activation in the trauma-avoidant identity state was shown in the left occipital gyrus and right occipital gyrus/calcarine sulcus. Decreased activation was found in the right superior frontal gyrus, left middle frontal gyrus and right cuneus/calcarine sulcus in the overt processing block (see supplementary Table 1 and Appendix B of the supplementary materials).

#### Between-group comparisons

Behavioural data revealed no between-group differences in reaction times between the two word types (*F*(1) = 0.039, *P* = 0.845, *η*_*p*_^2^ = 0.001). However, significant differences were shown between the overt and covert processing blocks, and between the trauma-avoidant and -related identity states (see Table 1). Post hoc pairwise comparisons showed slower reaction times for DID-G participants compared with the CTRL and DID-S groups. Slower reaction times were found for overt processing and trauma-related identity state.

Neural activity comparisons between the DID-G and CTRL groups showed increased activation in the left superior frontal gyrus and left inferior temporal gyrus in the trauma-avoidant identity state (see supplementary Table 1). In the trauma-related identity state there was increased activation in the bilateral precentral gyrus, bilateral postcentral gyrus, left supramarginal gyrus, right superior occipital gyrus, right cuneus and left calcarine sulcus. Increased activation was found in the right precentral gyrus in the overt processing block, and in the bilateral superior frontal gyrus, left inferior frontal gyrus, right postcentral gyrus and left lateral occipital gyrus in the covert processing block. Decreased activation was observed in the right middle temporal gyrus only in the trauma-avoidant identity state. In the overt processing block, decreased activation was found in the left middle frontal gyrus and right superior parietal lobule/supramarginal gyrus.

Neural data comparisons between the DID-G and DID-S groups showed increased activity in the bilateral superior and middle frontal gyrus, bilateral precentral and postcentral gyrus, bilateral superior parietal lobule, left supramarginal gyrus, bilateral occipital gyrus, right superior and transverse temporal gyrus and bilateral parahippocampal gyrus/frontal operculum, mainly in the trauma-related identity state. In the covert processing block, increased activation was observed in the right superior frontal gyrus, left middle and inferior frontal gyrus, bilateral precentral gyrus, right postcentral gyrus, left anterior insula, bilateral superior parietal lobule/precuneus and left supramarginal gyrus/inferior parietal lobule. Decreased activation was found in the left precentral gyrus and right postcentral gyrus only in the trauma-avoidant identity state. In the overt processing block, there was decreased activation of the right superior frontal gyrus, left middle frontal gyrus, right precentral gyrus, left precentral/postcentral gyrus, left superior parietal lobule, left parietal operculum, left lateral occipital gyrus/cuneus and right calcarine sulcus (see supplementary Table 1 and Appendix B of the supplementary materials).

## Discussion

The present study aimed to investigate the effect of overt and covert identity state-dependent processing of self-relevance knowledge in participants with diagnosed DID, DID-simulating controls and a paired group of PTSD and healthy controls. Our primary finding was that there were significant differences between overt and covert processing of self-relevant trauma-related words, with slower reaction times and increased brain activation for overt processing. Our second most important finding was that the elevated brain activity observed during overt self-relevance processing was mainly observed in the frontal gyrus in individuals with DID as compared with DID simulators and the paired control group, and was independent of dissociative identity state.

With regard to the overall effect of consciousness in the trauma-avoidant identity state of individuals with DID, the behavioural data show significantly slower reaction times in overt processing compared with covert which, paired with the increased frontal and parietal brain activation, indicates heightened cognitive involvement when consciously perceiving self-relevant words. Although the current findings support previous research proposing increased cognitive control and inter-identity avoidance in the trauma-avoidant identity state of DID,^2,3,5,8,23^ they are at odds with other studies. In particular, a study by Hermans and colleagues^25^ showed faster response times to emotional stimuli covertly presented in individuals diagnosed with DID in the trauma-avoidant identity state, and slower response times in their trauma-aware identity state, in direct contrast with the response patterns of the included control groups.^25^ Moreover, a study by Schlumpf and colleagues^10^ found longer reaction times to masked neutral faces for participants with DID when they were in a trauma-aware identity state as compared with when in a trauma-avoidant identity state. Lebois and colleagues^13^ used a mirror confrontation task and found that individuals with DID demonstrated reduced self-relevance ratings of their own face when presented covertly, as compared with control participants. The discrepancy of our findings relative to prior research could be attributed to methodological differences – that is, the subject-specific nature of the words used in our study as compared with standardised^10,13,25^ and individualised^13^ facial stimuli. Furthermore, Lebois and colleagues^13^ did not investigate the effect of distinct dissociative identity states on participants’ face perception. Nevertheless, our outcomes complement all aforementioned studies by emphasising the central role of consciousness of self-relevance and emotional perception in individuals with DID.

Results from our brain imaging data further support overt and covert processing differences, especially through the activation of frontal parts of the brain. Earlier imaging and electroencephalography studies have indicated marked neural response differences in relation to overt and covert processing between individuals with DID and healthy or simulating controls^10,12^ in the frontal, precentral, temporal and occipital gyri. Similarly, research on overt and covert processing in PTSD and its dissociative subtype reported significant differences in brain activation patterns following participants’ exposure to masked and unmasked emotional faces^26^ or general images.^27^ In accordance with our findings, overt exposure to emotional stimuli in PTSD showed increased activity in frontal regions, including the bilateral basal forebrain and bilateral supplementary motor area.^26^ Studies with PTSD participants have also demonstrated decreased activation of the medial prefrontal cortex,^28^ which encompasses regions corresponding to our findings such as the superior and middle frontal gyri. Furthermore, covert exposure to emotional images and trauma-related words showed increased activation in the visual cortex,^27^ including the occipital gyrus and calcarine sulcus, which is in agreement with our results. For further discussion of individual brain regions please refer to Appendix B of the supplementary materials.

Our outcome of frontal brain activity, paired with the increased activation in the intraparietal sulcus and parietal regions, corresponds with the frontoparietal network model, which has been implicated in cognitive control mechanisms^2,29^ including working memory processes.^19^ Notably, the frontal and parietal regions have also been associated with working memory and attention,^30^ further underlining their central role in cognitive processes. Increased frontal and parietal activity has further been observed in studies of PTSD^31^ and complex PTSD.^32^ Reinders and colleagues^4^ also proposed similar areas as a PTSD-based neurobiological model of DID with a strong involvement of parietal regions, in particular the intraparietal sulcus, which has also been confirmed by more recent research.^2^ Current outcomes further support this notion of increased cognitive control over, and mental avoidance of, trauma-related knowledge^2,32^ in the trauma-avoidant identity state^2,3,5,8,23^ leading to increased reaction time to trauma-related words.

The frontal lobe, and particularly the prefrontal cortices, have also been implicated in another PTSD-based neurobiological model previously proposed, the corticolimbic inhibition network.^5,23,33^ Specifically, this model proposes that, especially in the dissociative subtype of PTSD, emotional arousal leads to increased activation of the prefrontal cortex which, in turn, inhibits the activation of limbic structures such as the hippocampus and amygdala, thus reducing the emotional experience.^34^ Moreover, morphological abnormalities in the hippocampal area have previously been observed in individuals diagnosed with DID and associated with childhood maltreatment and dissociative symptomatology.^16^ Our results corroborate these previous findings because they show that the hippocampus was activated in the trauma-related identity state of participants with DID. Higher neural activation was also detected in the right parahippocampal gyrus in the trauma-related identity state, in line with earlier studies.^10^ Contrary to that model’s proposal of decreased activity in the insula,^3,4,33,34^ our findings show increased insula activation, supporting the majority of literature findings, as reported in the systematic review by Roydeva and Reinders.^35^

The outcomes of the current study hold significant clinical implications for the therapeutic treatment of DID, and provide valuable insights for future research in this field. As indicated by our findings, increased cognitive control during overt processing in the trauma-avoidant identity state indicates a way in which individuals with DID process trauma-related information, and highlights that interventions for DID could initially focus on addressing this cognitive avoidance.^2^ Moreover, our findings corroborate earlier outcomes on the neural biomarkers of DID^35^ and support PTSD-based neurobiological models,^4,5,23,29,34^ which emphasise the trauma-related nature of DID^2^ and, ultimately, the trauma-informed practices that clinicians should adopt in their assessments and interventions. In particular, approaches such as repetitive transcranial magnetic stimulation^2,29,35^ have previously been proposed for the treatment of disorders with increased frontoparietal activity. Real-time fMRI neurofeedback training has also been recommended as an intervention for emotion regulation, through activation modulation of the frontoparietal network.^36^ Although research is limited, this intervention’s effectiveness has been shown in studies including individuals with the dissociative subtype of PTSD.^37,38^ We suggest that future studies should investigate the efficacy of neurofeedback in altering the activation of the frontoparietal network in individuals diagnosed with DID.

The strength of our research study is the implementation of an event-related design while displaying trauma-related words rather than scripts (as in script-driven imagery studies^3–5,23^), which is more efficient in detecting transient neural responses in functional brain imaging studies. Another important strength of our study is the inclusion of multiple control groups, which allowed us to compare distinct brain activation patterns between diagnosed DID subjects and clinically healthy and simulating populations.

A limitation of our study is that our sample could be considered small, with limited statistical power. However, our sample is of similar size to others in the field and yielded significant outcomes. Furthermore, we used subject-specific self-relevant and trauma-related stimuli rather than standardised ones, which counteracts the limited sample size through the individualised investigation of our participants’ unique experiences. Lastly, the consistency of our findings with prior research in independent samples further affirms the validity of our outcomes (see also^2^). Another limitation of our study is that our specific design does not inform on intrusive phenomena in DID, but was tailored to explore behavioural and neural responses to self-relevant trauma-related knowledge. Future research should disentangle the issue of whether mental avoidance is relevant only to such material^39^ or whether it is an escape from an identity state, because identity states may be ‘phobic’ to each other.^40^ The results of such a study might offer further convergence on treatment recommendations.

In conclusion, our study reaffirms previous findings showing that overt self-relevance processing is characterised by slower reaction times and increased brain activity than covert processing, emphasising heightened cognitive control in the trauma-avoidant identity state of individuals with DID. This is mediated by increased activation of frontoparietal regions, associated with emotional memory suppression or avoidance, and the inhibition of limbic structures involved in emotional processing. These regions have also been highlighted in PTSD and therewith corroborate the notion that DID is a trauma-based disorder. Our study further emphasises that individuals with DID engage in activation of the frontoparietal network as a mechanism for mitigating the emotional impact of overtly presented subject-specific, trauma-related words, suggesting a neurobiological coping mechanism to manage severe trauma-related situations.

## Supporting information

Strouza et al. supplementary material 1 Strouza et al. supplementary material

Strouza et al. supplementary material 2 Strouza et al. supplementary material

## Data Availability

The data that support the findings of this study are available from the corresponding author, A.A.T.S.R., upon reasonable request.

## Supplementary material

The supplementary material is available online at https://doi.org/10.1192/bjo.2025.10914
