# Supplementary material for: Overt and covert processing of self-relevance information in dissociative identity disorder: controlled fMRI study
Source: BJPsych Open. 2025 Dec 26;12(1):e25. doi: 10.1192/bjo.2025.10914 (PMC12835690; doi:10.1192/bjo.2025.10914)
Supplement: Strouza et al. supplementary material 1 — Strouza et al. supplementary material [file S2056472425109149sup001.docx]

**SUPPLEMENTARY MATERIALS**

For the manuscript entitled “Overt and covert processing of self-relevance information in Dissociative Identity Disorder: A controlled fMRI study”

**Appendix A: Table of all words selected by participants during the baseline session, sorted first by participant Group, and then by highest rated word.**

Data were gathered in Amsterdam and Groningen, the Netherlands. The participants were invited to visit the research centres twice. During the first visit, they were asked to rate a list of 278 Dutch words, in terms of self-relevance intensity and emotional intensity. The list was originally obtained from a word evaluation study conducted with the general population (1) and supplemented with additional DID-specific trauma-related words (2,3). Each word could receive negative intensity values ranging from 0 (not-negative) to 4 (very negative) and self-relevance intensity scores ranging from 0 (not-self-relevant) to 2 (very self-relevant).

| **Group** | **State** | **TrialType** | **Text** | **eText** | **n** | **prop** |
| --- | --- | --- | --- | --- | --- | --- |
| DID-G^^n1^ | NIS | St | trauma | trauma | 11 | 79% |
| DID-G | NIS | NSt | roofmoord | robbery with murder | 11 | 79% |
| DID-G | NIS | NSt | wurgen | to strangle | 11 | 79% |
| DID-G | NIS | NSn | consulaat | consulate | 11 | 79% |
| DID-G | TIS | St | dwang | coercion or force | 10 | 71% |
| DID-G | NIS | NSt | atoombom | nuclear bomb | 10 | 71% |
| DID-G | NIS | NSt | martelen | to torture | 10 | 71% |
| DID-G | TIS | St | bang | afraid | 9 | 64% |
| DID-G | TIS | St | geweld | violence | 9 | 64% |
| DID-G | NIS | St | onmacht | powerlessness | 9 | 64% |
| DID-G | TIS | St | opsluiten | to lock up | 9 | 64% |
| DID-G | TIS | St | verkrachting | rape | 9 | 64% |
| DID-G | NIS | NSt | doodslag | manslaughter | 9 | 64% |
| DID-G | NIS | NSt | moord | murder | 9 | 64% |
| DID-G | TIS | NSn | ijzer | iron | 9 | 64% |
| DID-G | NIS | NSn | metselaar | bricklayer | 9 | 64% |
| DID-G | TIS | NSn | telegram | telegram | 9 | 64% |
| DID-G | TIS | NSn | zetel | seat | 9 | 64% |
| DID-G | TIS | St | angst | fear | 8 | 57% |
| DID-G | NIS | St | bang | afraid | 8 | 57% |
| DID-G | TIS | St | incest | incest | 8 | 57% |
| DID-G | NIS | St | onzeker | uncertain | 8 | 57% |
| DID-G | TIS | St | pijn | pain | 8 | 57% |
| DID-G | TIS | St | seks | sex | 8 | 57% |
| DID-G | NIS | St | verkrachting | rape | 8 | 57% |
| DID-G | TIS | NSt | atoombom | nuclear bomb | 8 | 57% |
| DID-G | NIS | NSt | bloedbad | bloodbath | 8 | 57% |
| DID-G | NIS | NSt | doden | to kill | 8 | 57% |
| DID-G | NIS | NSt | misdrijf | crime | 8 | 57% |
| DID-G | TIS | NSt | oorlog | war | 8 | 57% |
| DID-G | NIS | NSt | pedofiel | paedophile | 8 | 57% |
| DID-G | TIS | NSt | roofmoord | robbery with murder | 8 | 57% |
| DID-G | NIS | NSt | tiran | tyrant | 8 | 57% |
| DID-G | TIS | NSn | cirkel | circle | 8 | 57% |
| DID-G | TIS | NSn | magazijn | warehouse | 8 | 57% |
| DID-G | TIS | NSn | metaal | metal | 8 | 57% |
| DID-G | TIS | NSn | metselaar | bricklayer | 8 | 57% |
| DID-G | TIS | NSn | pilaar | pillar | 8 | 57% |
| DID-G | TIS | NSn | rad | wheel | 8 | 57% |
| DID-G | TIS | NSn | stellen | to set | 8 | 57% |
| DID-G | TIS | NSn | stomerij | drycleaner | 8 | 57% |
| DID-G | TIS | NSn | zegel | seal | 8 | 57% |
| DID-G | TIS | St | afsnauwen | to snap at | 7 | 50% |
| DID-G | NIS | St | afwijzing | rejection | 7 | 50% |
| DID-G | NIS | St | angst | fear | 7 | 50% |
| DID-G | TIS | St | domkop | idiot | 7 | 50% |
| DID-G | NIS | St | dwang | coercion or force | 7 | 50% |
| DID-G | NIS | St | dwingen | to coerce or to force | 7 | 50% |
| DID-G | NIS | St | geweld | violence | 7 | 50% |
| DID-G | TIS | St | haten | to hate | 7 | 50% |
| DID-G | TIS | St | kreunen | to moan | 7 | 50% |
| DID-G | TIS | St | onmacht | powerlessness | 7 | 50% |
| DID-G | NIS | St | schuldig | guilty | 7 | 50% |
| DID-G | TIS | St | schuldig | guilty | 7 | 50% |
| DID-G | TIS | St | stikken | to suffocate | 7 | 50% |
| DID-G | TIS | St | walging | disgust | 7 | 50% |
| DID-G | NIS | NSt | beroerte | stroke | 7 | 50% |
| DID-G | NIS | NSt | folteren | to torture | 7 | 50% |
| DID-G | NIS | NSt | oorlog | war | 7 | 50% |
| DID-G | NIS | NSt | orgie | orgy | 7 | 50% |
| DID-G | TIS | NSn | dakgoot | gutter | 7 | 50% |
| DID-G | NIS | NSn | deurknop | door knob | 7 | 50% |
| DID-G | NIS | NSn | firma | firm | 7 | 50% |
| DID-G | TIS | NSn | gebouw | building | 7 | 50% |
| DID-G | NIS | NSn | haak | hook | 7 | 50% |
| DID-G | NIS | NSn | kast | closet | 7 | 50% |
| DID-G | NIS | NSn | kozijn | window frame | 7 | 50% |
| DID-G | NIS | NSn | plank | shelf | 7 | 50% |
| DID-G | TIS | NSn | schroef | screw | 7 | 50% |
| DID-G | TIS | NSn | tegel | tile | 7 | 50% |
| DID-G | NIS | NSn | telegram | telegram | 7 | 50% |
| DID-G | NIS | NSn | trottoir | pavement | 7 | 50% |
| DID-G | NIS | NSn | versie | version | 7 | 50% |
| DID-G | NIS | NSn | vierkant | square | 7 | 50% |
| DID-G | NIS | St | alleen | alone | 6 | 43% |
| DID-G | TIS | St | alleen | alone | 6 | 43% |
| DID-G | NIS | St | conflict | conflict | 6 | 43% |
| DID-G | TIS | St | doorslikken | to swallow | 6 | 43% |
| DID-G | NIS | St | droefheid | sadness | 6 | 43% |
| DID-G | TIS | St | ellende | misery | 6 | 43% |
| DID-G | NIS | St | falen | to fail | 6 | 43% |
| DID-G | NIS | St | haten | to hate | 6 | 43% |
| DID-G | NIS | St | incest | incest | 6 | 43% |
| DID-G | NIS | St | ongewenst | unwanted | 6 | 43% |
| DID-G | NIS | St | ruzie | fight or quarrel | 6 | 43% |
| DID-G | NIS | St | seks | sex | 6 | 43% |
| DID-G | TIS | St | slet | slut | 6 | 43% |
| DID-G | NIS | St | stikken | to suffocate | 6 | 43% |
| DID-G | NIS | St | tegenslag | setback | 6 | 43% |
| DID-G | NIS | St | vader | father | 6 | 43% |
| DID-G | NIS | St | verdriet | sadness | 6 | 43% |
| DID-G | NIS | NSt | bordeel | brothel | 6 | 43% |
| DID-G | NIS | NSt | crimineel | criminal | 6 | 43% |
| DID-G | NIS | NSt | gezwel | tumor | 6 | 43% |
| DID-G | NIS | NSt | incest | incest | 6 | 43% |
| DID-G | NIS | NSt | misdaad | crime | 6 | 43% |
| DID-G | NIS | NSt | sadist | sadist | 6 | 43% |
| DID-G | NIS | NSt | treiteren | to harass | 6 | 43% |
| DID-G | TIS | NSn | consulaat | consulate | 6 | 43% |
| DID-G | TIS | NSn | dwerg | dwarf | 6 | 43% |
| DID-G | TIS | NSn | flacon | bottle or vial | 6 | 43% |
| DID-G | NIS | NSn | ijzer | iron | 6 | 43% |
| DID-G | NIS | NSn | ivoor | ivory | 6 | 43% |
| DID-G | NIS | NSn | kenteken | license plate | 6 | 43% |
| DID-G | NIS | NSn | kogel | bullet | 6 | 43% |
| DID-G | NIS | NSn | omroep | broadcasting | 6 | 43% |
| DID-G | TIS | NSn | omroep | broadcasting | 6 | 43% |
| DID-G | TIS | NSn | pasen | Easter | 6 | 43% |
| DID-G | NIS | NSn | regenton | rain barrel | 6 | 43% |
| DID-G | NIS | NSn | register | register | 6 | 43% |
| DID-G | NIS | NSn | roeren | to stir | 6 | 43% |
| DID-G | NIS | NSn | schroef | screw | 6 | 43% |
| DID-G | NIS | NSn | steil | steep | 6 | 43% |
| DID-G | TIS | NSn | tapijt | tapestry | 6 | 43% |
| DID-G | NIS | NSn | tegel | tile | 6 | 43% |
| DID-G | NIS | NSn | teller | counter | 6 | 43% |
| DID-G | TIS | NSn | theelepel | teaspoon | 6 | 43% |
| DID-G | TIS | NSn | trede | step | 6 | 43% |
| DID-G | TIS | NSn | trottoir | pavement | 6 | 43% |
| DID-G | TIS | NSn | vierkant | square | 6 | 43% |
| DID-G | NIS | NSn | zakje | little bag | 6 | 43% |
| DID-G | TIS | NSn | zandloper | hourglass | 6 | 43% |
| DID-G | TIS | St | afkeer | aversion | 5 | 36% |
| DID-G | TIS | St | bedreigen | to threaten | 5 | 36% |
| DID-G | TIS | St | bloedbad | bloodbath | 5 | 36% |
| DID-G | NIS | St | boos | angry | 5 | 36% |
| DID-G | TIS | St | buurman | neighbor | 5 | 36% |
| DID-G | TIS | St | doodgaan | to die | 5 | 36% |
| DID-G | NIS | St | ellende | misery | 5 | 36% |
| DID-G | TIS | St | folteren | to torture | 5 | 36% |
| DID-G | TIS | St | hijgen | to pant | 5 | 36% |
| DID-G | TIS | St | kreng | bitch | 5 | 36% |
| DID-G | TIS | St | lijden | to suffer | 5 | 36% |
| DID-G | TIS | St | man | man | 5 | 36% |
| DID-G | TIS | St | martelen | to torture | 5 | 36% |
| DID-G | NIS | St | misdrijf | crime | 5 | 36% |
| DID-G | NIS | St | moeder | mother | 5 | 36% |
| DID-G | NIS | St | pedofiel | paedophile | 5 | 36% |
| DID-G | TIS | St | slecht | bad | 5 | 36% |
| DID-G | TIS | St | stinken | to stink | 5 | 36% |
| DID-G | TIS | St | trappen | to kick | 5 | 36% |
| DID-G | TIS | St | trauma | trauma | 5 | 36% |
| DID-G | NIS | St | verlammen | to paralyze | 5 | 36% |
| DID-G | NIS | St | verraad | betrayal | 5 | 36% |
| DID-G | NIS | St | wanhoop | despair | 5 | 36% |
| DID-G | TIS | St | wreedheid | cruelty | 5 | 36% |
| DID-G | TIS | St | wurgen | to strangle | 5 | 36% |
| DID-G | TIS | St | zeer | ache | 5 | 36% |
| DID-G | TIS | NSt | armoede | poverty | 5 | 36% |
| DID-G | TIS | NSt | inbraak | burglary | 5 | 36% |
| DID-G | TIS | NSt | kanker | cancer | 5 | 36% |
| DID-G | NIS | NSt | lepra | leprosy | 5 | 36% |
| DID-G | TIS | NSt | lepra | leprosy | 5 | 36% |
| DID-G | NIS | NSt | messteek | knife stab | 5 | 36% |
| DID-G | NIS | NSt | miskraam | miscarriage | 5 | 36% |
| DID-G | NIS | NSt | mismaakt | deformed | 5 | 36% |
| DID-G | TIS | NSt | slet | slut | 5 | 36% |
| DID-G | NIS | NSt | steekwond | stab wound | 5 | 36% |
| DID-G | NIS | NSt | stikken | to suffocate | 5 | 36% |
| DID-G | NIS | NSt | verkrachting | rape | 5 | 36% |
| DID-G | NIS | NSt | vernielen | to vandalize | 5 | 36% |
| DID-G | NIS | NSt | verzuipen | to drown | 5 | 36% |
| DID-G | NIS | NSt | walging | disgust | 5 | 36% |
| DID-G | NIS | NSn | absorptie | absorption | 5 | 36% |
| DID-G | TIS | NSn | absorptie | absorption | 5 | 36% |
| DID-G | NIS | NSn | bestek | cutlery | 5 | 36% |
| DID-G | TIS | NSn | bladzijde | page | 5 | 36% |
| DID-G | TIS | NSn | citaat | quote | 5 | 36% |
| DID-G | NIS | NSn | dakgoot | gutter | 5 | 36% |
| DID-G | TIS | NSn | deurknop | door knob | 5 | 36% |
| DID-G | NIS | NSn | dozijn | dozen | 5 | 36% |
| DID-G | NIS | NSn | dwerg | dwarf | 5 | 36% |
| DID-G | TIS | NSn | element | element | 5 | 36% |
| DID-G | TIS | NSn | firma | firm | 5 | 36% |
| DID-G | NIS | NSn | flacon | bottle or vial | 5 | 36% |
| DID-G | NIS | NSn | gebouw | building | 5 | 36% |
| DID-G | NIS | NSn | grondstof | natural resource | 5 | 36% |
| DID-G | TIS | NSn | grondstof | natural resource | 5 | 36% |
| DID-G | NIS | NSn | hertogin | duchess | 5 | 36% |
| DID-G | TIS | NSn | ivoor | ivory | 5 | 36% |
| DID-G | TIS | NSn | juni | June | 5 | 36% |
| DID-G | TIS | NSn | kenteken | license plate | 5 | 36% |
| DID-G | TIS | NSn | laden | to load | 5 | 36% |
| DID-G | NIS | NSn | legpuzzel | jigsaw puzzle | 5 | 36% |
| DID-G | NIS | NSn | leuning | railing | 5 | 36% |
| DID-G | TIS | NSn | leuning | railing | 5 | 36% |
| DID-G | NIS | NSn | magazijn | warehouse | 5 | 36% |
| DID-G | NIS | NSn | metaal | metal | 5 | 36% |
| DID-G | TIS | NSn | plafond | ceiling | 5 | 36% |
| DID-G | TIS | NSn | plank | shelf | 5 | 36% |
| DID-G | NIS | NSn | regel | rule | 5 | 36% |
| DID-G | TIS | NSn | roeren | to stir | 5 | 36% |
| DID-G | NIS | NSn | stoelpoot | chair leg | 5 | 36% |
| DID-G | NIS | NSn | stomerij | drycleaner | 5 | 36% |
| DID-G | NIS | NSn | stukadoor | plasterer | 5 | 36% |
| DID-G | TIS | NSn | stukadoor | plasterer | 5 | 36% |
| DID-G | NIS | NSn | tapijt | tapestry | 5 | 36% |
| DID-G | TIS | NSn | teller | counter | 5 | 36% |
| DID-G | TIS | NSn | tijdperk | era | 5 | 36% |
| DID-G | NIS | NSn | traject | route | 5 | 36% |
| DID-G | NIS | NSn | trede | step | 5 | 36% |
| DID-G | NIS | NSn | treden | to step | 5 | 36% |
| DID-G | NIS | NSn | uitgever | publisher | 5 | 36% |
| DID-G | NIS | NSn | zegel | seal | 5 | 36% |
| DID-G | TIS | St | achterlaten | leave behind | 4 | 29% |
| DID-G | NIS | St | afkeer | aversion | 4 | 29% |
| DID-G | NIS | St | afkraken | to decry | 4 | 29% |
| DID-G | NIS | St | afscheid | goodbye | 4 | 29% |
| DID-G | TIS | St | afwijzing | rejection | 4 | 29% |
| DID-G | NIS | St | agressie | aggression | 4 | 29% |
| DID-G | TIS | St | bed | bed | 4 | 29% |
| DID-G | NIS | St | benauwen | to agitate | 4 | 29% |
| DID-G | TIS | St | boos | angry | 4 | 29% |
| DID-G | TIS | St | branden | to burn | 4 | 29% |
| DID-G | NIS | St | depressie | depression | 4 | 29% |
| DID-G | NIS | St | domkop | idiot | 4 | 29% |
| DID-G | NIS | St | doodgaan | to die | 4 | 29% |
| DID-G | TIS | St | doodslag | manslaughter | 4 | 29% |
| DID-G | TIS | St | doodsteek | deathblow | 4 | 29% |
| DID-G | TIS | St | dwingen | to coerce or to force | 4 | 29% |
| DID-G | NIS | St | kelder | basement | 4 | 29% |
| DID-G | TIS | St | kelder | basement | 4 | 29% |
| DID-G | NIS | St | lawaai | noise | 4 | 29% |
| DID-G | NIS | St | lijden | to suffer | 4 | 29% |
| DID-G | TIS | St | meester | master | 4 | 29% |
| DID-G | NIS | St | mislukt | failed | 4 | 29% |
| DID-G | NIS | St | nacht | night | 4 | 29% |
| DID-G | NIS | St | omkomen | to perish | 4 | 29% |
| DID-G | TIS | St | ongewenst | unwanted | 4 | 29% |
| DID-G | TIS | St | pedofiel | paedophile | 4 | 29% |
| DID-G | NIS | St | piekeren | to mull | 4 | 29% |
| DID-G | NIS | St | pijn | pain | 4 | 29% |
| DID-G | NIS | St | slecht | bad | 4 | 29% |
| DID-G | NIS | St | snauwen | to snarl | 4 | 29% |
| DID-G | TIS | St | vader | father | 4 | 29% |
| DID-G | TIS | St | verdriet | sadness | 4 | 29% |
| DID-G | TIS | St | verzuipen | to drown | 4 | 29% |
| DID-G | TIS | St | wanhoop | despair | 4 | 29% |
| DID-G | NIS | St | zeer | ache | 4 | 29% |
| DID-G | NIS | NSt | afblaffen | to bark at | 4 | 29% |
| DID-G | NIS | NSt | afgunst | envy | 4 | 29% |
| DID-G | TIS | NSt | afschuw | revulsion | 4 | 29% |
| DID-G | NIS | NSt | agressie | aggression | 4 | 29% |
| DID-G | NIS | NSt | bedreigen | to threaten | 4 | 29% |
| DID-G | TIS | NSt | bedrieger | deceiver | 4 | 29% |
| DID-G | TIS | NSt | bedrog | deceit | 4 | 29% |
| DID-G | TIS | NSt | bordeel | brothel | 4 | 29% |
| DID-G | NIS | NSt | chanteren | to blackmail | 4 | 29% |
| DID-G | TIS | NSt | crimineel | criminal | 4 | 29% |
| DID-G | NIS | NSt | diefstal | theft | 4 | 29% |
| DID-G | TIS | NSt | doodslag | manslaughter | 4 | 29% |
| DID-G | NIS | NSt | doodsteek | deathblow | 4 | 29% |
| DID-G | NIS | NSt | geweld | violence | 4 | 29% |
| DID-G | NIS | NSt | gijzeling | kidnapping | 4 | 29% |
| DID-G | TIS | NSt | gijzeling | kidnapping | 4 | 29% |
| DID-G | NIS | NSt | kanker | cancer | 4 | 29% |
| DID-G | NIS | NSt | kreng | bitch | 4 | 29% |
| DID-G | NIS | NSt | krenken | to hurt | 4 | 29% |
| DID-G | NIS | NSt | omkomen | to perish | 4 | 29% |
| DID-G | NIS | NSt | opsluiten | to lock up | 4 | 29% |
| DID-G | TIS | NSt | pedofiel | paedophile | 4 | 29% |
| DID-G | NIS | NSt | slet | slut | 4 | 29% |
| DID-G | TIS | NSt | slijmen | suck up to | 4 | 29% |
| DID-G | NIS | NSt | snauwen | to snarl | 4 | 29% |
| DID-G | NIS | NSt | verdrinken | to drown | 4 | 29% |
| DID-G | NIS | NSt | verraad | betrayal | 4 | 29% |
| DID-G | NIS | NSt | wreedheid | cruelty | 4 | 29% |
| DID-G | TIS | NSt | wurgen | to strangle | 4 | 29% |
| DID-G | NIS | NSn | aanmaken | to prepare | 4 | 29% |
| DID-G | TIS | NSn | aanmaken | to prepare | 4 | 29% |
| DID-G | TIS | NSn | bestek | cutlery | 4 | 29% |
| DID-G | TIS | NSn | blikje | can | 4 | 29% |
| DID-G | NIS | NSn | element | element | 4 | 29% |
| DID-G | NIS | NSn | elleboog | elbow | 4 | 29% |
| DID-G | TIS | NSn | elleboog | elbow | 4 | 29% |
| DID-G | TIS | NSn | etiket | label | 4 | 29% |
| DID-G | NIS | NSn | geit | goat | 4 | 29% |
| DID-G | TIS | NSn | hagedis | lizard | 4 | 29% |
| DID-G | NIS | NSn | juni | June | 4 | 29% |
| DID-G | TIS | NSn | kast | closet | 4 | 29% |
| DID-G | NIS | NSn | meester | master | 4 | 29% |
| DID-G | NIS | NSn | middel | middle | 4 | 29% |
| DID-G | NIS | NSn | mond | mouth | 4 | 29% |
| DID-G | TIS | NSn | octaaf | octave | 4 | 29% |
| DID-G | TIS | NSn | ontslag | resignation or dismissal | 4 | 29% |
| DID-G | NIS | NSn | paragraaf | paragraph | 4 | 29% |
| DID-G | NIS | NSn | pilaar | pillar | 4 | 29% |
| DID-G | NIS | NSn | plafond | ceiling | 4 | 29% |
| DID-G | NIS | NSn | postzegel | stamp | 4 | 29% |
| DID-G | TIS | NSn | potlood | pencil | 4 | 29% |
| DID-G | NIS | NSn | rad | wheel | 4 | 29% |
| DID-G | TIS | NSn | regenton | rain barrel | 4 | 29% |
| DID-G | NIS | NSn | schuren | to polish | 4 | 29% |
| DID-G | NIS | NSn | stellen | to set | 4 | 29% |
| DID-G | NIS | NSn | stoel | chair | 4 | 29% |
| DID-G | TIS | NSn | stoelpoot | chair leg | 4 | 29% |
| DID-G | TIS | NSn | takken | branches | 4 | 29% |
| DID-G | NIS | NSn | theelepel | teaspoon | 4 | 29% |
| DID-G | NIS | NSn | tijdperk | era | 4 | 29% |
| DID-G | TIS | NSn | traject | route | 4 | 29% |
| DID-G | TIS | NSn | treden | to step | 4 | 29% |
| DID-G | TIS | NSn | uitgever | publisher | 4 | 29% |
| DID-G | NIS | NSn | vergroten | enlarge | 4 | 29% |
| DID-G | NIS | NSn | zandloper | hourglass | 4 | 29% |
| DID-G | TIS | St | afgrijzen | horror | 3 | 21% |
| DID-G | NIS | St | afschuw | revulsion | 3 | 21% |
| DID-G | NIS | St | afsnauwen | to snap at | 3 | 21% |
| DID-G | TIS | St | agressie | aggression | 3 | 21% |
| DID-G | NIS | St | armoede | poverty | 3 | 21% |
| DID-G | NIS | St | belazerd | fooled | 3 | 21% |
| DID-G | TIS | St | belazerd | fooled | 3 | 21% |
| DID-G | NIS | St | bloedbad | bloodbath | 3 | 21% |
| DID-G | NIS | St | doodslag | manslaughter | 3 | 21% |
| DID-G | NIS | St | doodsteek | deathblow | 3 | 21% |
| DID-G | NIS | St | doorslikken | to swallow | 3 | 21% |
| DID-G | TIS | St | droefheid | sadness | 3 | 21% |
| DID-G | NIS | St | ergeren | to annoy | 3 | 21% |
| DID-G | NIS | St | ergernis | annoyance | 3 | 21% |
| DID-G | NIS | St | etiket | label | 3 | 21% |
| DID-G | TIS | St | handen | hands | 3 | 21% |
| DID-G | NIS | St | hijgen | to pant | 3 | 21% |
| DID-G | TIS | St | hysterie | hysteria | 3 | 21% |
| DID-G | NIS | St | instorten | to collapse | 3 | 21% |
| DID-G | NIS | St | kotsen | to vomit | 3 | 21% |
| DID-G | NIS | St | kou | cold | 3 | 21% |
| DID-G | TIS | St | kuthoer | pussy whore | 3 | 21% |
| DID-G | NIS | St | liegen | to lie | 3 | 21% |
| DID-G | NIS | St | man | man | 3 | 21% |
| DID-G | NIS | St | martelen | to torture | 3 | 21% |
| DID-G | NIS | St | miskraam | miscarriage | 3 | 21% |
| DID-G | TIS | St | moeder | mother | 3 | 21% |
| DID-G | NIS | St | moord | murder | 3 | 21% |
| DID-G | NIS | St | ontrouw | unfaithful | 3 | 21% |
| DID-G | TIS | St | oom | uncle | 3 | 21% |
| DID-G | NIS | St | opsluiten | to lock up | 3 | 21% |
| DID-G | NIS | St | piemel | willy | 3 | 21% |
| DID-G | TIS | St | ruzie | fight or quarrel | 3 | 21% |
| DID-G | TIS | St | sadist | sadist | 3 | 21% |
| DID-G | NIS | St | schok | shock | 3 | 21% |
| DID-G | TIS | St | schoppen | to kick | 3 | 21% |
| DID-G | TIS | St | snijden | to cut | 3 | 21% |
| DID-G | TIS | St | stank | stench | 3 | 21% |
| DID-G | TIS | St | steekwond | stab wound | 3 | 21% |
| DID-G | TIS | St | treiteren | to harass | 3 | 21% |
| DID-G | NIS | St | uitgescholden | scolded | 3 | 21% |
| DID-G | TIS | St | uitlachen | to laugh at | 3 | 21% |
| DID-G | TIS | St | vastbinden | to tie | 3 | 21% |
| DID-G | TIS | St | verdrinken | to drown | 3 | 21% |
| DID-G | TIS | St | vies | dirty | 3 | 21% |
| DID-G | NIS | St | wanhopen | to despair | 3 | 21% |
| DID-G | TIS | St | woest | enraged | 3 | 21% |
| DID-G | NIS | St | zeuren | to nag | 3 | 21% |
| DID-G | TIS | St | zondebok | scapegoat | 3 | 21% |
| DID-G | NIS | NSt | aanslag | attack | 3 | 21% |
| DID-G | TIS | NSt | aanslag | attack | 3 | 21% |
| DID-G | TIS | NSt | afwijzing | rejection | 3 | 21% |
| DID-G | NIS | NSt | angst | fear | 3 | 21% |
| DID-G | NIS | NSt | bedrieger | deceiver | 3 | 21% |
| DID-G | NIS | NSt | bedrog | deceit | 3 | 21% |
| DID-G | TIS | NSt | beroerte | stroke | 3 | 21% |
| DID-G | TIS | NSt | besmetten | to contaminate | 3 | 21% |
| DID-G | NIS | NSt | blinddoek | blindfold | 3 | 21% |
| DID-G | TIS | NSt | bloedbad | bloodbath | 3 | 21% |
| DID-G | NIS | NSt | boos | angry | 3 | 21% |
| DID-G | TIS | NSt | diefstal | theft | 3 | 21% |
| DID-G | NIS | NSt | etter | pus | 3 | 21% |
| DID-G | NIS | NSt | getreiter | harassment | 3 | 21% |
| DID-G | NIS | NSt | hoer | whore | 3 | 21% |
| DID-G | NIS | NSt | inbraak | burglary | 3 | 21% |
| DID-G | TIS | NSt | incest | incest | 3 | 21% |
| DID-G | NIS | NSt | instorten | to collapse | 3 | 21% |
| DID-G | NIS | NSt | kuthoer | pussy whore | 3 | 21% |
| DID-G | TIS | NSt | kuthoer | pussy whore | 3 | 21% |
| DID-G | NIS | NSt | kwetsen | to hurt | 3 | 21% |
| DID-G | TIS | NSt | messteek | knife stab | 3 | 21% |
| DID-G | NIS | NSt | noodkreet | cry for help | 3 | 21% |
| DID-G | NIS | NSt | onrecht | injustice | 3 | 21% |
| DID-G | NIS | NSt | ontrouw | unfaithful | 3 | 21% |
| DID-G | TIS | NSt | ontrouw | unfaithful | 3 | 21% |
| DID-G | NIS | NSt | oplichten | to scam | 3 | 21% |
| DID-G | TIS | NSt | oplichten | to scam | 3 | 21% |
| DID-G | TIS | NSt | orgie | orgy | 3 | 21% |
| DID-G | NIS | NSt | piemel | willy | 3 | 21% |
| DID-G | TIS | NSt | ruzie | fight or quarrel | 3 | 21% |
| DID-G | NIS | NSt | schande | shame | 3 | 21% |
| DID-G | NIS | NSt | slaaf | slave | 3 | 21% |
| DID-G | TIS | NSt | snijden | to cut | 3 | 21% |
| DID-G | TIS | NSt | steekwond | stab wound | 3 | 21% |
| DID-G | NIS | NSt | stinken | to stink | 3 | 21% |
| DID-G | TIS | NSt | verdord | withered | 3 | 21% |
| DID-G | NIS | NSt | wanhoop | despair | 3 | 21% |
| DID-G | TIS | NSt | wraak | revenge | 3 | 21% |
| DID-G | TIS | NSn | atoombom | nuclear bomb | 3 | 21% |
| DID-G | TIS | NSn | bad | bath | 3 | 21% |
| DID-G | NIS | NSn | begraven | to bury | 3 | 21% |
| DID-G | NIS | NSn | chauffeur | driver | 3 | 21% |
| DID-G | NIS | NSn | cirkel | circle | 3 | 21% |
| DID-G | NIS | NSn | citaat | quote | 3 | 21% |
| DID-G | NIS | NSn | document | document | 3 | 21% |
| DID-G | NIS | NSn | etiket | label | 3 | 21% |
| DID-G | NIS | NSn | hagedis | lizard | 3 | 21% |
| DID-G | TIS | NSn | hertogin | duchess | 3 | 21% |
| DID-G | TIS | NSn | kerk | church | 3 | 21% |
| DID-G | NIS | NSn | krabben | to scratch | 3 | 21% |
| DID-G | NIS | NSn | laden | to load | 3 | 21% |
| DID-G | TIS | NSn | lepra | leprosy | 3 | 21% |
| DID-G | NIS | NSn | ogen | eyes | 3 | 21% |
| DID-G | NIS | NSn | ontslag | resignation or dismissal | 3 | 21% |
| DID-G | TIS | NSn | paragraaf | paragraph | 3 | 21% |
| DID-G | NIS | NSn | pasen | Easter | 3 | 21% |
| DID-G | TIS | NSn | postzegel | stamp | 3 | 21% |
| DID-G | TIS | NSn | register | register | 3 | 21% |
| DID-G | TIS | NSn | schuren | to polish | 3 | 21% |
| DID-G | TIS | NSn | stoep | sidewalk | 3 | 21% |
| DID-G | NIS | NSn | touw | rope | 3 | 21% |
| DID-G | TIS | NSn | touw | rope | 3 | 21% |
| DID-G | NIS | NSn | trappen | to kick | 3 | 21% |
| DID-G | TIS | NSn | vergroten | enlarge | 3 | 21% |
| DID-G | NIS | NSn | vreemde | stranger | 3 | 21% |
| DID-G | TIS | NSn | zakje | little bag | 3 | 21% |
| DID-G | NIS | NSn | zetel | seat | 3 | 21% |
| DID-G | TIS | St | afblaffen | to bark at | 2 | 14% |
| DID-G | NIS | St | afgunst | envy | 2 | 14% |
| DID-G | TIS | St | afkraken | to decry | 2 | 14% |
| DID-G | TIS | St | armoede | poverty | 2 | 14% |
| DID-G | NIS | St | bed | bed | 2 | 14% |
| DID-G | NIS | St | bedreigen | to threaten | 2 | 14% |
| DID-G | NIS | St | bedrieger | deceiver | 2 | 14% |
| DID-G | NIS | St | begraven | to bury | 2 | 14% |
| DID-G | TIS | St | benauwen | to agitate | 2 | 14% |
| DID-G | TIS | St | besmetten | to contaminate | 2 | 14% |
| DID-G | NIS | St | bloot | naked | 2 | 14% |
| DID-G | TIS | St | bordeel | brothel | 2 | 14% |
| DID-G | NIS | St | branden | to burn | 2 | 14% |
| DID-G | NIS | St | buurman | neighbor | 2 | 14% |
| DID-G | NIS | St | chanteren | to blackmail | 2 | 14% |
| DID-G | TIS | St | chanteren | to blackmail | 2 | 14% |
| DID-G | TIS | St | conflict | conflict | 2 | 14% |
| DID-G | TIS | St | crimineel | criminal | 2 | 14% |
| DID-G | NIS | St | doden | to kill | 2 | 14% |
| DID-G | TIS | St | dokter | (general practice) doctor | 2 | 14% |
| DID-G | NIS | St | dreigen | to threaten | 2 | 14% |
| DID-G | TIS | St | droevig | sad | 2 | 14% |
| DID-G | NIS | St | dubbel | double | 2 | 14% |
| DID-G | NIS | St | etter | pus | 2 | 14% |
| DID-G | NIS | St | folteren | to torture | 2 | 14% |
| DID-G | TIS | St | gaatje | little hole | 2 | 14% |
| DID-G | NIS | St | handen | hands | 2 | 14% |
| DID-G | NIS | St | hoer | whore | 2 | 14% |
| DID-G | TIS | St | instorten | to collapse | 2 | 14% |
| DID-G | NIS | St | kader | framework | 2 | 14% |
| DID-G | NIS | St | kanker | cancer | 2 | 14% |
| DID-G | NIS | St | kast | closet | 2 | 14% |
| DID-G | TIS | St | kast | closet | 2 | 14% |
| DID-G | NIS | St | kerk | church | 2 | 14% |
| DID-G | TIS | St | kotsen | to vomit | 2 | 14% |
| DID-G | TIS | St | kou | cold | 2 | 14% |
| DID-G | NIS | St | kwetsen | to hurt | 2 | 14% |
| DID-G | TIS | St | kwetsen | to hurt | 2 | 14% |
| DID-G | TIS | St | lafaard | coward | 2 | 14% |
| DID-G | TIS | St | likken | to lick | 2 | 14% |
| DID-G | NIS | St | masker | mask | 2 | 14% |
| DID-G | TIS | St | masker | mask | 2 | 14% |
| DID-G | NIS | St | messteek | knife stab | 2 | 14% |
| DID-G | TIS | St | messteek | knife stab | 2 | 14% |
| DID-G | TIS | St | misdaad | crime | 2 | 14% |
| DID-G | TIS | St | misdrijf | crime | 2 | 14% |
| DID-G | NIS | St | moedeloos | despondent | 2 | 14% |
| DID-G | TIS | St | nacht | night | 2 | 14% |
| DID-G | TIS | St | ongeluk | accident | 2 | 14% |
| DID-G | NIS | St | onrecht | injustice | 2 | 14% |
| DID-G | TIS | St | onrecht | injustice | 2 | 14% |
| DID-G | TIS | St | onzeker | uncertain | 2 | 14% |
| DID-G | NIS | St | oom | uncle | 2 | 14% |
| DID-G | TIS | St | piemel | willy | 2 | 14% |
| DID-G | NIS | St | sadist | sadist | 2 | 14% |
| DID-G | NIS | St | schoft | bastard | 2 | 14% |
| DID-G | TIS | St | schuren | to polish | 2 | 14% |
| DID-G | TIS | St | slaaf | slave | 2 | 14% |
| DID-G | TIS | St | snauwen | to snarl | 2 | 14% |
| DID-G | NIS | St | snijden | to cut | 2 | 14% |
| DID-G | NIS | St | stank | stench | 2 | 14% |
| DID-G | NIS | St | steil | steep | 2 | 14% |
| DID-G | TIS | St | stiekem | secretly | 2 | 14% |
| DID-G | NIS | St | tiran | tyrant | 2 | 14% |
| DID-G | TIS | St | tiran | tyrant | 2 | 14% |
| DID-G | NIS | St | trappen | to kick | 2 | 14% |
| DID-G | NIS | St | treiteren | to harass | 2 | 14% |
| DID-G | NIS | St | vastbinden | to tie | 2 | 14% |
| DID-G | NIS | St | verbranden | to burn | 2 | 14% |
| DID-G | TIS | St | vergroten | enlarge | 2 | 14% |
| DID-G | TIS | St | verlammen | to paralyze | 2 | 14% |
| DID-G | TIS | St | verleidster | temptress | 2 | 14% |
| DID-G | TIS | St | verraad | betrayal | 2 | 14% |
| DID-G | NIS | St | verzuipen | to drown | 2 | 14% |
| DID-G | NIS | St | vies | dirty | 2 | 14% |
| DID-G | NIS | St | vuur | fire | 2 | 14% |
| DID-G | TIS | St | wanhopen | to despair | 2 | 14% |
| DID-G | NIS | St | woest | enraged | 2 | 14% |
| DID-G | NIS | St | wurgen | to strangle | 2 | 14% |
| DID-G | TIS | St | zuigen | to suck | 2 | 14% |
| DID-G | NIS | NSt | achterlaten | leave behind | 2 | 14% |
| DID-G | TIS | NSt | achterlaten | leave behind | 2 | 14% |
| DID-G | TIS | NSt | afgunst | envy | 2 | 14% |
| DID-G | TIS | NSt | afhakken | to chop off | 2 | 14% |
| DID-G | NIS | NSt | afkraken | to decry | 2 | 14% |
| DID-G | TIS | NSt | afscheid | goodbye | 2 | 14% |
| DID-G | NIS | NSt | afschuw | revulsion | 2 | 14% |
| DID-G | NIS | NSt | armoede | poverty | 2 | 14% |
| DID-G | NIS | NSt | besmetten | to contaminate | 2 | 14% |
| DID-G | TIS | NSt | conflict | conflict | 2 | 14% |
| DID-G | NIS | NSt | depressie | depression | 2 | 14% |
| DID-G | TIS | NSt | doodsteek | deathblow | 2 | 14% |
| DID-G | NIS | NSt | dreigen | to threaten | 2 | 14% |
| DID-G | NIS | NSt | dwang | coercion or force | 2 | 14% |
| DID-G | NIS | NSt | embryo | embryo | 2 | 14% |
| DID-G | TIS | NSt | etter | pus | 2 | 14% |
| DID-G | TIS | NSt | folteren | to torture | 2 | 14% |
| DID-G | TIS | NSt | gezwel | tumor | 2 | 14% |
| DID-G | NIS | NSt | haten | to hate | 2 | 14% |
| DID-G | NIS | NSt | hijgen | to pant | 2 | 14% |
| DID-G | TIS | NSt | hijgen | to pant | 2 | 14% |
| DID-G | TIS | NSt | hoer | whore | 2 | 14% |
| DID-G | NIS | NSt | hysterie | hysteria | 2 | 14% |
| DID-G | TIS | NSt | hysterie | hysteria | 2 | 14% |
| DID-G | NIS | NSt | kelder | basement | 2 | 14% |
| DID-G | NIS | NSt | knijpen | to pinch | 2 | 14% |
| DID-G | NIS | NSt | kotsen | to vomit | 2 | 14% |
| DID-G | TIS | NSt | kotsen | to vomit | 2 | 14% |
| DID-G | NIS | NSt | kreunen | to moan | 2 | 14% |
| DID-G | NIS | NSt | laden | to load | 2 | 14% |
| DID-G | TIS | NSt | lafaard | coward | 2 | 14% |
| DID-G | NIS | NSt | liegen | to lie | 2 | 14% |
| DID-G | TIS | NSt | liegen | to lie | 2 | 14% |
| DID-G | NIS | NSt | lijden | to suffer | 2 | 14% |
| DID-G | NIS | NSt | likken | to lick | 2 | 14% |
| DID-G | NIS | NSt | masker | mask | 2 | 14% |
| DID-G | NIS | NSt | meeloper | opportunist | 2 | 14% |
| DID-G | TIS | NSt | meeloper | opportunist | 2 | 14% |
| DID-G | TIS | NSt | misdaad | crime | 2 | 14% |
| DID-G | TIS | NSt | misdrijf | crime | 2 | 14% |
| DID-G | NIS | NSt | mislukt | failed | 2 | 14% |
| DID-G | NIS | NSt | ongeluk | accident | 2 | 14% |
| DID-G | TIS | NSt | ongeval | accident | 2 | 14% |
| DID-G | NIS | NSt | ontslag | resignation or dismissal | 2 | 14% |
| DID-G | TIS | NSt | oom | uncle | 2 | 14% |
| DID-G | NIS | NSt | razernij | fury | 2 | 14% |
| DID-G | TIS | NSt | schoft | bastard | 2 | 14% |
| DID-G | NIS | NSt | schuldig | guilty | 2 | 14% |
| DID-G | TIS | NSt | slaaf | slave | 2 | 14% |
| DID-G | NIS | NSt | snijden | to cut | 2 | 14% |
| DID-G | NIS | NSt | stank | stench | 2 | 14% |
| DID-G | NIS | NSt | stiekem | secretly | 2 | 14% |
| DID-G | TIS | NSt | stiekem | secretly | 2 | 14% |
| DID-G | TIS | NSt | stikken | to suffocate | 2 | 14% |
| DID-G | TIS | NSt | tegenslag | setback | 2 | 14% |
| DID-G | NIS | NSt | trappen | to kick | 2 | 14% |
| DID-G | NIS | NSt | uitjouwen | to boo | 2 | 14% |
| DID-G | TIS | NSt | uitjouwen | to boo | 2 | 14% |
| DID-G | NIS | NSt | uitkleden | to undress | 2 | 14% |
| DID-G | TIS | NSt | verbranden | to burn | 2 | 14% |
| DID-G | NIS | NSt | verlammen | to paralyze | 2 | 14% |
| DID-G | NIS | NSt | verleidster | temptress | 2 | 14% |
| DID-G | TIS | NSt | verleidster | temptress | 2 | 14% |
| DID-G | NIS | NSt | vies | dirty | 2 | 14% |
| DID-G | NIS | NSt | wraak | revenge | 2 | 14% |
| DID-G | TIS | NSn | aanslag | attack | 2 | 14% |
| DID-G | TIS | NSn | achterlaten | leave behind | 2 | 14% |
| DID-G | NIS | NSn | afhakken | to chop off | 2 | 14% |
| DID-G | NIS | NSn | bad | bath | 2 | 14% |
| DID-G | NIS | NSn | benauwen | to agitate | 2 | 14% |
| DID-G | NIS | NSn | bladzijde | page | 2 | 14% |
| DID-G | NIS | NSn | blikje | can | 2 | 14% |
| DID-G | NIS | NSn | bloot | naked | 2 | 14% |
| DID-G | TIS | NSn | broer | brother | 2 | 14% |
| DID-G | TIS | NSn | chauffeur | driver | 2 | 14% |
| DID-G | NIS | NSn | conflict | conflict | 2 | 14% |
| DID-G | TIS | NSn | depressie | depression | 2 | 14% |
| DID-G | TIS | NSn | doodsteek | deathblow | 2 | 14% |
| DID-G | TIS | NSn | dozijn | dozen | 2 | 14% |
| DID-G | TIS | NSn | dubbel | double | 2 | 14% |
| DID-G | NIS | NSn | embryo | embryo | 2 | 14% |
| DID-G | TIS | NSn | embryo | embryo | 2 | 14% |
| DID-G | NIS | NSn | ergeren | to annoy | 2 | 14% |
| DID-G | TIS | NSn | gaatje | little hole | 2 | 14% |
| DID-G | TIS | NSn | geit | goat | 2 | 14% |
| DID-G | TIS | NSn | haak | hook | 2 | 14% |
| DID-G | TIS | NSn | haken | hooks | 2 | 14% |
| DID-G | NIS | NSn | handen | hands | 2 | 14% |
| DID-G | TIS | NSn | handen | hands | 2 | 14% |
| DID-G | TIS | NSn | hysterie | hysteria | 2 | 14% |
| DID-G | TIS | NSn | instorten | to collapse | 2 | 14% |
| DID-G | NIS | NSn | kader | framework | 2 | 14% |
| DID-G | TIS | NSn | kader | framework | 2 | 14% |
| DID-G | NIS | NSn | kou | cold | 2 | 14% |
| DID-G | NIS | NSn | kreunen | to moan | 2 | 14% |
| DID-G | TIS | NSn | legpuzzel | jigsaw puzzle | 2 | 14% |
| DID-G | NIS | NSn | lepra | leprosy | 2 | 14% |
| DID-G | NIS | NSn | likken | to lick | 2 | 14% |
| DID-G | TIS | NSn | middel | middle | 2 | 14% |
| DID-G | NIS | NSn | misleiden | to deceive | 2 | 14% |
| DID-G | TIS | NSn | misvormen | to deform | 2 | 14% |
| DID-G | NIS | NSn | moedeloos | despondent | 2 | 14% |
| DID-G | TIS | NSn | moord | murder | 2 | 14% |
| DID-G | NIS | NSn | octaaf | octave | 2 | 14% |
| DID-G | TIS | NSn | onmacht | powerlessness | 2 | 14% |
| DID-G | NIS | NSn | ontrouw | unfaithful | 2 | 14% |
| DID-G | NIS | NSn | oom | uncle | 2 | 14% |
| DID-G | TIS | NSn | oplichten | to scam | 2 | 14% |
| DID-G | NIS | NSn | orgie | orgy | 2 | 14% |
| DID-G | NIS | NSn | potlood | pencil | 2 | 14% |
| DID-G | NIS | NSn | snijden | to cut | 2 | 14% |
| DID-G | TIS | NSn | stank | stench | 2 | 14% |
| DID-G | TIS | NSn | steekwond | stab wound | 2 | 14% |
| DID-G | TIS | NSn | steil | steep | 2 | 14% |
| DID-G | TIS | NSn | stikken | to suffocate | 2 | 14% |
| DID-G | NIS | NSn | stinken | to stink | 2 | 14% |
| DID-G | TIS | NSn | stoel | chair | 2 | 14% |
| DID-G | NIS | NSn | stoep | sidewalk | 2 | 14% |
| DID-G | NIS | NSn | takken | branches | 2 | 14% |
| DID-G | TIS | NSn | tegenslag | setback | 2 | 14% |
| DID-G | TIS | NSn | tiran | tyrant | 2 | 14% |
| DID-G | TIS | NSn | uitjouwen | to boo | 2 | 14% |
| DID-G | NIS | NSn | uitlachen | to laugh at | 2 | 14% |
| DID-G | NIS | NSn | verdord | withered | 2 | 14% |
| DID-G | TIS | NSn | verdord | withered | 2 | 14% |
| DID-G | TIS | NSn | woest | enraged | 2 | 14% |
| DID-G | NIS | NSn | zeer | ache | 2 | 14% |
| DID-G | NIS | NSn | zuigen | to suck | 2 | 14% |
| DID-G | NIS | St | achterlaten | leave behind | 1 | 7% |
| DID-G | NIS | St | afblaffen | to bark at | 1 | 7% |
| DID-G | NIS | St | afgrijzen | horror | 1 | 7% |
| DID-G | TIS | St | afscheid | goodbye | 1 | 7% |
| DID-G | TIS | St | afschuw | revulsion | 1 | 7% |
| DID-G | TIS | St | baby | baby | 1 | 7% |
| DID-G | NIS | St | bedrog | deceit | 1 | 7% |
| DID-G | TIS | St | bedrog | deceit | 1 | 7% |
| DID-G | NIS | St | besmetten | to contaminate | 1 | 7% |
| DID-G | NIS | St | blinddoek | blindfold | 1 | 7% |
| DID-G | TIS | St | blinddoek | blindfold | 1 | 7% |
| DID-G | TIS | St | bloot | naked | 1 | 7% |
| DID-G | NIS | St | broer | brother | 1 | 7% |
| DID-G | NIS | St | chauffeur | driver | 1 | 7% |
| DID-G | TIS | St | cirkel | circle | 1 | 7% |
| DID-G | NIS | St | crimineel | criminal | 1 | 7% |
| DID-G | TIS | St | depressie | depression | 1 | 7% |
| DID-G | TIS | St | doden | to kill | 1 | 7% |
| DID-G | TIS | St | dreigen | to threaten | 1 | 7% |
| DID-G | NIS | St | droevig | sad | 1 | 7% |
| DID-G | TIS | St | dubbel | double | 1 | 7% |
| DID-G | NIS | St | elleboog | elbow | 1 | 7% |
| DID-G | TIS | St | embryo | embryo | 1 | 7% |
| DID-G | TIS | St | ergeren | to annoy | 1 | 7% |
| DID-G | TIS | St | ergernis | annoyance | 1 | 7% |
| DID-G | NIS | St | getreiter | harassment | 1 | 7% |
| DID-G | NIS | St | gezwel | tumor | 1 | 7% |
| DID-G | TIS | St | gezwel | tumor | 1 | 7% |
| DID-G | NIS | St | gijzeling | kidnapping | 1 | 7% |
| DID-G | TIS | St | gijzeling | kidnapping | 1 | 7% |
| DID-G | TIS | St | ijzer | iron | 1 | 7% |
| DID-G | TIS | St | kanker | cancer | 1 | 7% |
| DID-G | TIS | St | kerk | church | 1 | 7% |
| DID-G | NIS | St | knijpen | to pinch | 1 | 7% |
| DID-G | TIS | St | knijpen | to pinch | 1 | 7% |
| DID-G | NIS | St | kogel | bullet | 1 | 7% |
| DID-G | NIS | St | kreng | bitch | 1 | 7% |
| DID-G | NIS | St | krenken | to hurt | 1 | 7% |
| DID-G | TIS | St | krenken | to hurt | 1 | 7% |
| DID-G | NIS | St | lafaard | coward | 1 | 7% |
| DID-G | TIS | St | lawaai | noise | 1 | 7% |
| DID-G | NIS | St | leugen | lie | 1 | 7% |
| DID-G | TIS | St | liegen | to lie | 1 | 7% |
| DID-G | NIS | St | mes | knife | 1 | 7% |
| DID-G | TIS | St | mes | knife | 1 | 7% |
| DID-G | TIS | St | middel | middle | 1 | 7% |
| DID-G | TIS | St | miskraam | miscarriage | 1 | 7% |
| DID-G | TIS | St | misleiden | to deceive | 1 | 7% |
| DID-G | TIS | St | mislukt | failed | 1 | 7% |
| DID-G | TIS | St | mismaakt | deformed | 1 | 7% |
| DID-G | TIS | St | moedeloos | despondent | 1 | 7% |
| DID-G | TIS | St | moord | murder | 1 | 7% |
| DID-G | NIS | St | noodkreet | cry for help | 1 | 7% |
| DID-G | TIS | St | noodkreet | cry for help | 1 | 7% |
| DID-G | NIS | St | ogen | eyes | 1 | 7% |
| DID-G | TIS | St | ogen | eyes | 1 | 7% |
| DID-G | TIS | St | omkomen | to perish | 1 | 7% |
| DID-G | NIS | St | ongeluk | accident | 1 | 7% |
| DID-G | NIS | St | ongeval | accident | 1 | 7% |
| DID-G | TIS | St | ontrouw | unfaithful | 1 | 7% |
| DID-G | NIS | St | ontslag | resignation or dismissal | 1 | 7% |
| DID-G | NIS | St | oorlog | war | 1 | 7% |
| DID-G | NIS | St | oplichten | to scam | 1 | 7% |
| DID-G | NIS | St | orgie | orgy | 1 | 7% |
| DID-G | TIS | St | orgie | orgy | 1 | 7% |
| DID-G | NIS | St | plafond | ceiling | 1 | 7% |
| DID-G | TIS | St | plafond | ceiling | 1 | 7% |
| DID-G | NIS | St | rad | wheel | 1 | 7% |
| DID-G | TIS | St | razernij | fury | 1 | 7% |
| DID-G | NIS | St | regel | rule | 1 | 7% |
| DID-G | TIS | St | regel | rule | 1 | 7% |
| DID-G | NIS | St | roofmoord | robbery with murder | 1 | 7% |
| DID-G | TIS | St | roofmoord | robbery with murder | 1 | 7% |
| DID-G | NIS | St | schande | shame | 1 | 7% |
| DID-G | TIS | St | schande | shame | 1 | 7% |
| DID-G | TIS | St | schoft | bastard | 1 | 7% |
| DID-G | TIS | St | schok | shock | 1 | 7% |
| DID-G | NIS | St | schoppen | to kick | 1 | 7% |
| DID-G | TIS | St | slijmen | suck up to | 1 | 7% |
| DID-G | NIS | St | spoelen | to flush or to rinse | 1 | 7% |
| DID-G | NIS | St | steekwond | stab wound | 1 | 7% |
| DID-G | NIS | St | sterven | to die | 1 | 7% |
| DID-G | NIS | St | stinken | to stink | 1 | 7% |
| DID-G | NIS | St | stoelpoot | chair leg | 1 | 7% |
| DID-G | TIS | St | tegenslag | setback | 1 | 7% |
| DID-G | TIS | St | treden | to step | 1 | 7% |
| DID-G | NIS | St | uitkleden | to undress | 1 | 7% |
| DID-G | TIS | St | uitkleden | to undress | 1 | 7% |
| DID-G | NIS | St | uitlachen | to laugh at | 1 | 7% |
| DID-G | TIS | St | verbranden | to burn | 1 | 7% |
| DID-G | TIS | St | verdord | withered | 1 | 7% |
| DID-G | NIS | St | vergroten | enlarge | 1 | 7% |
| DID-G | NIS | St | vernielen | to vandalize | 1 | 7% |
| DID-G | NIS | St | vierkant | square | 1 | 7% |
| DID-G | NIS | St | wraak | revenge | 1 | 7% |
| DID-G | TIS | St | zeuren | to nag | 1 | 7% |
| DID-G | NIS | St | zondebok | scapegoat | 1 | 7% |
| DID-G | NIS | St | zuigen | to suck | 1 | 7% |
| DID-G | TIS | NSt | absorptie | absorption | 1 | 7% |
| DID-G | NIS | NSt | afgrijzen | horror | 1 | 7% |
| DID-G | TIS | NSt | afgrijzen | horror | 1 | 7% |
| DID-G | NIS | NSt | afhakken | to chop off | 1 | 7% |
| DID-G | NIS | NSt | afkeer | aversion | 1 | 7% |
| DID-G | TIS | NSt | afkraken | to decry | 1 | 7% |
| DID-G | NIS | NSt | afwijzing | rejection | 1 | 7% |
| DID-G | TIS | NSt | agressie | aggression | 1 | 7% |
| DID-G | NIS | NSt | baby | baby | 1 | 7% |
| DID-G | NIS | NSt | bang | afraid | 1 | 7% |
| DID-G | TIS | NSt | bedreigen | to threaten | 1 | 7% |
| DID-G | NIS | NSt | begraven | to bury | 1 | 7% |
| DID-G | TIS | NSt | begraven | to bury | 1 | 7% |
| DID-G | NIS | NSt | beklemmen | to oppress | 1 | 7% |
| DID-G | TIS | NSt | beklemmen | to oppress | 1 | 7% |
| DID-G | NIS | NSt | belazerd | fooled | 1 | 7% |
| DID-G | TIS | NSt | belazerd | fooled | 1 | 7% |
| DID-G | TIS | NSt | blinddoek | blindfold | 1 | 7% |
| DID-G | NIS | NSt | bloot | naked | 1 | 7% |
| DID-G | TIS | NSt | chanteren | to blackmail | 1 | 7% |
| DID-G | NIS | NSt | chauffeur | driver | 1 | 7% |
| DID-G | NIS | NSt | conflict | conflict | 1 | 7% |
| DID-G | TIS | NSt | depressie | depression | 1 | 7% |
| DID-G | NIS | NSt | deurknop | door knob | 1 | 7% |
| DID-G | TIS | NSt | doden | to kill | 1 | 7% |
| DID-G | NIS | NSt | domkop | idiot | 1 | 7% |
| DID-G | NIS | NSt | doodgaan | to die | 1 | 7% |
| DID-G | NIS | NSt | doorslikken | to swallow | 1 | 7% |
| DID-G | TIS | NSt | droefheid | sadness | 1 | 7% |
| DID-G | TIS | NSt | droevig | sad | 1 | 7% |
| DID-G | NIS | NSt | dwingen | to coerce or to force | 1 | 7% |
| DID-G | NIS | NSt | elleboog | elbow | 1 | 7% |
| DID-G | NIS | NSt | ellende | misery | 1 | 7% |
| DID-G | NIS | NSt | ergeren | to annoy | 1 | 7% |
| DID-G | TIS | NSt | ergeren | to annoy | 1 | 7% |
| DID-G | NIS | NSt | ergernis | annoyance | 1 | 7% |
| DID-G | TIS | NSt | firma | firm | 1 | 7% |
| DID-G | NIS | NSt | gaatje | little hole | 1 | 7% |
| DID-G | TIS | NSt | getreiter | harassment | 1 | 7% |
| DID-G | TIS | NSt | geweld | violence | 1 | 7% |
| DID-G | NIS | NSt | haak | hook | 1 | 7% |
| DID-G | TIS | NSt | haak | hook | 1 | 7% |
| DID-G | TIS | NSt | hagedis | lizard | 1 | 7% |
| DID-G | NIS | NSt | haken | hooks | 1 | 7% |
| DID-G | NIS | NSt | handen | hands | 1 | 7% |
| DID-G | TIS | NSt | ivoor | ivory | 1 | 7% |
| DID-G | NIS | NSt | kerk | church | 1 | 7% |
| DID-G | NIS | NSt | kogel | bullet | 1 | 7% |
| DID-G | TIS | NSt | kogel | bullet | 1 | 7% |
| DID-G | NIS | NSt | krabben | to scratch | 1 | 7% |
| DID-G | TIS | NSt | kreng | bitch | 1 | 7% |
| DID-G | TIS | NSt | kreunen | to moan | 1 | 7% |
| DID-G | NIS | NSt | lafaard | coward | 1 | 7% |
| DID-G | TIS | NSt | lawaai | noise | 1 | 7% |
| DID-G | NIS | NSt | legpuzzel | jigsaw puzzle | 1 | 7% |
| DID-G | NIS | NSt | leugen | lie | 1 | 7% |
| DID-G | TIS | NSt | lijden | to suffer | 1 | 7% |
| DID-G | TIS | NSt | likken | to lick | 1 | 7% |
| DID-G | TIS | NSt | man | man | 1 | 7% |
| DID-G | TIS | NSt | masker | mask | 1 | 7% |
| DID-G | TIS | NSt | mes | knife | 1 | 7% |
| DID-G | TIS | NSt | miskraam | miscarriage | 1 | 7% |
| DID-G | TIS | NSt | misleiden | to deceive | 1 | 7% |
| DID-G | TIS | NSt | mismaakt | deformed | 1 | 7% |
| DID-G | NIS | NSt | misvormen | to deform | 1 | 7% |
| DID-G | TIS | NSt | misvormen | to deform | 1 | 7% |
| DID-G | TIS | NSt | moedeloos | despondent | 1 | 7% |
| DID-G | TIS | NSt | moeder | mother | 1 | 7% |
| DID-G | TIS | NSt | moord | murder | 1 | 7% |
| DID-G | TIS | NSt | ongeluk | accident | 1 | 7% |
| DID-G | NIS | NSt | ongeval | accident | 1 | 7% |
| DID-G | NIS | NSt | ongewenst | unwanted | 1 | 7% |
| DID-G | NIS | NSt | onmacht | powerlessness | 1 | 7% |
| DID-G | TIS | NSt | ontslag | resignation or dismissal | 1 | 7% |
| DID-G | NIS | NSt | plafond | ceiling | 1 | 7% |
| DID-G | NIS | NSt | plank | shelf | 1 | 7% |
| DID-G | NIS | NSt | rad | wheel | 1 | 7% |
| DID-G | TIS | NSt | razernij | fury | 1 | 7% |
| DID-G | TIS | NSt | regel | rule | 1 | 7% |
| DID-G | NIS | NSt | ruzie | fight or quarrel | 1 | 7% |
| DID-G | NIS | NSt | schaden | to damage | 1 | 7% |
| DID-G | TIS | NSt | schande | shame | 1 | 7% |
| DID-G | NIS | NSt | schoft | bastard | 1 | 7% |
| DID-G | NIS | NSt | schok | shock | 1 | 7% |
| DID-G | TIS | NSt | schok | shock | 1 | 7% |
| DID-G | NIS | NSt | schoppen | to kick | 1 | 7% |
| DID-G | TIS | NSt | schuldig | guilty | 1 | 7% |
| DID-G | NIS | NSt | slecht | bad | 1 | 7% |
| DID-G | NIS | NSt | spoelen | to flush or to rinse | 1 | 7% |
| DID-G | NIS | NSt | sterven | to die | 1 | 7% |
| DID-G | TIS | NSt | sterven | to die | 1 | 7% |
| DID-G | TIS | NSt | stinken | to stink | 1 | 7% |
| DID-G | NIS | NSt | stoep | sidewalk | 1 | 7% |
| DID-G | NIS | NSt | teller | counter | 1 | 7% |
| DID-G | TIS | NSt | trappen | to kick | 1 | 7% |
| DID-G | NIS | NSt | trauma | trauma | 1 | 7% |
| DID-G | TIS | NSt | treiteren | to harass | 1 | 7% |
| DID-G | NIS | NSt | uitgescholden | scolded | 1 | 7% |
| DID-G | TIS | NSt | uitgescholden | scolded | 1 | 7% |
| DID-G | NIS | NSt | uitlachen | to laugh at | 1 | 7% |
| DID-G | NIS | NSt | vastbinden | to tie | 1 | 7% |
| DID-G | NIS | NSt | verbranden | to burn | 1 | 7% |
| DID-G | TIS | NSt | verdrinken | to drown | 1 | 7% |
| DID-G | TIS | NSt | verkrachting | rape | 1 | 7% |
| DID-G | TIS | NSt | verlammen | to paralyze | 1 | 7% |
| DID-G | TIS | NSt | vernielen | to vandalize | 1 | 7% |
| DID-G | TIS | NSt | verzuipen | to drown | 1 | 7% |
| DID-G | TIS | NSt | vreemde | stranger | 1 | 7% |
| DID-G | TIS | NSt | walging | disgust | 1 | 7% |
| DID-G | NIS | NSt | wanhopen | to despair | 1 | 7% |
| DID-G | NIS | NSt | woest | enraged | 1 | 7% |
| DID-G | NIS | NSt | zeuren | to nag | 1 | 7% |
| DID-G | TIS | NSt | zeuren | to nag | 1 | 7% |
| DID-G | NIS | NSt | zondebok | scapegoat | 1 | 7% |
| DID-G | NIS | NSt | zuigen | to suck | 1 | 7% |
| DID-G | TIS | NSt | zuigen | to suck | 1 | 7% |
| DID-G | NIS | NSn | aanslag | attack | 1 | 7% |
| DID-G | NIS | NSn | achterlaten | leave behind | 1 | 7% |
| DID-G | NIS | NSn | afblaffen | to bark at | 1 | 7% |
| DID-G | TIS | NSn | afblaffen | to bark at | 1 | 7% |
| DID-G | NIS | NSn | afgunst | envy | 1 | 7% |
| DID-G | TIS | NSn | afkeer | aversion | 1 | 7% |
| DID-G | TIS | NSn | afkraken | to decry | 1 | 7% |
| DID-G | NIS | NSn | afscheid | goodbye | 1 | 7% |
| DID-G | TIS | NSn | afschuw | revulsion | 1 | 7% |
| DID-G | NIS | NSn | alleen | alone | 1 | 7% |
| DID-G | NIS | NSn | armoede | poverty | 1 | 7% |
| DID-G | TIS | NSn | armoede | poverty | 1 | 7% |
| DID-G | NIS | NSn | atoombom | nuclear bomb | 1 | 7% |
| DID-G | TIS | NSn | baby | baby | 1 | 7% |
| DID-G | NIS | NSn | bang | afraid | 1 | 7% |
| DID-G | NIS | NSn | bed | bed | 1 | 7% |
| DID-G | TIS | NSn | bed | bed | 1 | 7% |
| DID-G | NIS | NSn | bedrog | deceit | 1 | 7% |
| DID-G | TIS | NSn | begraven | to bury | 1 | 7% |
| DID-G | NIS | NSn | beklemmen | to oppress | 1 | 7% |
| DID-G | TIS | NSn | beklemmen | to oppress | 1 | 7% |
| DID-G | TIS | NSn | blinddoek | blindfold | 1 | 7% |
| DID-G | TIS | NSn | bloot | naked | 1 | 7% |
| DID-G | NIS | NSn | bordeel | brothel | 1 | 7% |
| DID-G | TIS | NSn | bordeel | brothel | 1 | 7% |
| DID-G | NIS | NSn | branden | to burn | 1 | 7% |
| DID-G | NIS | NSn | broer | brother | 1 | 7% |
| DID-G | NIS | NSn | buurman | neighbor | 1 | 7% |
| DID-G | TIS | NSn | buurman | neighbor | 1 | 7% |
| DID-G | NIS | NSn | chanteren | to blackmail | 1 | 7% |
| DID-G | TIS | NSn | conflict | conflict | 1 | 7% |
| DID-G | NIS | NSn | crimineel | criminal | 1 | 7% |
| DID-G | TIS | NSn | diefstal | theft | 1 | 7% |
| DID-G | TIS | NSn | dokter | (general practice) doctor | 1 | 7% |
| DID-G | NIS | NSn | domkop | idiot | 1 | 7% |
| DID-G | TIS | NSn | domkop | idiot | 1 | 7% |
| DID-G | NIS | NSn | doodgaan | to die | 1 | 7% |
| DID-G | NIS | NSn | doodsteek | deathblow | 1 | 7% |
| DID-G | NIS | NSn | doorslikken | to swallow | 1 | 7% |
| DID-G | NIS | NSn | dubbel | double | 1 | 7% |
| DID-G | NIS | NSn | dwingen | to coerce or to force | 1 | 7% |
| DID-G | TIS | NSn | ergeren | to annoy | 1 | 7% |
| DID-G | NIS | NSn | ergernis | annoyance | 1 | 7% |
| DID-G | NIS | NSn | etter | pus | 1 | 7% |
| DID-G | NIS | NSn | falen | to fail | 1 | 7% |
| DID-G | NIS | NSn | gaatje | little hole | 1 | 7% |
| DID-G | TIS | NSn | gezwel | tumor | 1 | 7% |
| DID-G | NIS | NSn | gijzeling | kidnapping | 1 | 7% |
| DID-G | NIS | NSn | haken | hooks or to hook | 1 | 7% |
| DID-G | NIS | NSn | haten | to hate | 1 | 7% |
| DID-G | TIS | NSn | huisdier | pet | 1 | 7% |
| DID-G | NIS | NSn | hysterie | hysteria | 1 | 7% |
| DID-G | NIS | NSn | inbraak | burglary | 1 | 7% |
| DID-G | TIS | NSn | inbraak | burglary | 1 | 7% |
| DID-G | NIS | NSn | incest | incest | 1 | 7% |
| DID-G | NIS | NSn | kelder | basement | 1 | 7% |
| DID-G | TIS | NSn | kelder | basement | 1 | 7% |
| DID-G | TIS | NSn | knijpen | to pinch | 1 | 7% |
| DID-G | TIS | NSn | kogel | bullet | 1 | 7% |
| DID-G | TIS | NSn | kozijn | window frame | 1 | 7% |
| DID-G | TIS | NSn | krabben | to scratch | 1 | 7% |
| DID-G | TIS | NSn | kreng | bitch | 1 | 7% |
| DID-G | NIS | NSn | krenken | to hurt | 1 | 7% |
| DID-G | TIS | NSn | krenken | to hurt | 1 | 7% |
| DID-G | NIS | NSn | kwetsen | to hurt | 1 | 7% |
| DID-G | NIS | NSn | lawaai | noise | 1 | 7% |
| DID-G | NIS | NSn | liegen | to lie | 1 | 7% |
| DID-G | NIS | NSn | man | man | 1 | 7% |
| DID-G | TIS | NSn | martelen | to torture | 1 | 7% |
| DID-G | NIS | NSn | mes | knife | 1 | 7% |
| DID-G | TIS | NSn | mes | knife | 1 | 7% |
| DID-G | TIS | NSn | misdrijf | crime | 1 | 7% |
| DID-G | NIS | NSn | miskraam | miscarriage | 1 | 7% |
| DID-G | TIS | NSn | misleiden | to deceive | 1 | 7% |
| DID-G | TIS | NSn | mislukt | failed | 1 | 7% |
| DID-G | TIS | NSn | mismaakt | deformed | 1 | 7% |
| DID-G | NIS | NSn | nacht | night | 1 | 7% |
| DID-G | NIS | NSn | omkomen | to perish | 1 | 7% |
| DID-G | TIS | NSn | omkomen | to perish | 1 | 7% |
| DID-G | NIS | NSn | ongeluk | accident | 1 | 7% |
| DID-G | TIS | NSn | ongeluk | accident | 1 | 7% |
| DID-G | NIS | NSn | ongeval | accident | 1 | 7% |
| DID-G | TIS | NSn | ongeval | accident | 1 | 7% |
| DID-G | NIS | NSn | onzeker | uncertain | 1 | 7% |
| DID-G | TIS | NSn | oorlog | war | 1 | 7% |
| DID-G | TIS | NSn | orgie | orgy | 1 | 7% |
| DID-G | TIS | NSn | pedofiel | paedophile | 1 | 7% |
| DID-G | NIS | NSn | piekeren | to mull | 1 | 7% |
| DID-G | TIS | NSn | regel | rule | 1 | 7% |
| DID-G | TIS | NSn | roofmoord | robbery with murder | 1 | 7% |
| DID-G | NIS | NSn | ruzie | fight or quarrel | 1 | 7% |
| DID-G | TIS | NSn | sadist | sadist | 1 | 7% |
| DID-G | TIS | NSn | schaden | to damage | 1 | 7% |
| DID-G | TIS | NSn | schoft | bastard | 1 | 7% |
| DID-G | NIS | NSn | schok | shock | 1 | 7% |
| DID-G | NIS | NSn | schoppen | to kick | 1 | 7% |
| DID-G | NIS | NSn | slet | slut | 1 | 7% |
| DID-G | NIS | NSn | snauwen | to snarl | 1 | 7% |
| DID-G | TIS | NSn | snijden | to cut | 1 | 7% |
| DID-G | TIS | NSn | spoelen | to flush or to rinse | 1 | 7% |
| DID-G | NIS | NSn | stank | stench | 1 | 7% |
| DID-G | NIS | NSn | steekwond | stab wound | 1 | 7% |
| DID-G | TIS | NSn | sterven | to die | 1 | 7% |
| DID-G | NIS | NSn | stiekem | secretly | 1 | 7% |
| DID-G | NIS | NSn | stikken | to suffocate | 1 | 7% |
| DID-G | TIS | NSn | trauma | trauma | 1 | 7% |
| DID-G | NIS | NSn | uitjouwen | to boo | 1 | 7% |
| DID-G | NIS | NSn | uitkleden | to undress | 1 | 7% |
| DID-G | TIS | NSn | uitlachen | to laugh at | 1 | 7% |
| DID-G | NIS | NSn | vader | father | 1 | 7% |
| DID-G | NIS | NSn | verdrinken | to drown | 1 | 7% |
| DID-G | NIS | NSn | verleidster | temptress | 1 | 7% |
| DID-G | TIS | NSn | verleidster | temptress | 1 | 7% |
| DID-G | TIS | NSn | verraad | betrayal | 1 | 7% |
| DID-G | TIS | NSn | versie | version | 1 | 7% |
| DID-G | NIS | NSn | vuur | fire | 1 | 7% |
| DID-G | TIS | NSn | vuur | fire | 1 | 7% |
| DID-G | TIS | NSn | wraak | revenge | 1 | 7% |
| DID-G | TIS | NSn | wurgen | to strangle | 1 | 7% |
| DID-G | TIS | NSn | zeuren | to nag | 1 | 7% |
| DID-G | NIS | NSn | zondebok | scapegoat | 1 | 7% |
| DID-G | TIS | NSn | zondebok | scapegoat | 1 | 7% |
| DID-S^^n2^ | NIS | NSt | verkrachting | rape | 14 | 100% |
| DID-S | TIS | NSn | tijdperk | era | 11 | 79% |
| DID-S | TIS | St | verkrachting | rape | 10 | 71% |
| DID-S | TIS | St | walging | disgust | 10 | 71% |
| DID-S | NIS | NSt | bloedbad | bloodbath | 10 | 71% |
| DID-S | NIS | NSt | crimineel | criminal | 10 | 71% |
| DID-S | NIS | NSt | incest | incest | 10 | 71% |
| DID-S | TIS | NSt | kanker | cancer | 10 | 71% |
| DID-S | NIS | NSt | verdrinken | to drown | 10 | 71% |
| DID-S | TIS | NSn | grondstof | natural resource | 10 | 71% |
| DID-S | NIS | NSn | schroef | screw | 10 | 71% |
| DID-S | TIS | NSn | telegram | telegram | 10 | 71% |
| DID-S | NIS | St | afwijzing | rejection | 9 | 64% |
| DID-S | TIS | St | geweld | violence | 9 | 64% |
| DID-S | NIS | NSt | folteren | to torture | 9 | 64% |
| DID-S | NIS | NSt | geweld | violence | 9 | 64% |
| DID-S | NIS | NSt | martelen | to torture | 9 | 64% |
| DID-S | NIS | NSt | messteek | knife stab | 9 | 64% |
| DID-S | NIS | NSt | miskraam | miscarriage | 9 | 64% |
| DID-S | TIS | NSt | oorlog | war | 9 | 64% |
| DID-S | NIS | NSt | pedofiel | paedophile | 9 | 64% |
| DID-S | NIS | NSn | grondstof | natural resource | 9 | 64% |
| DID-S | TIS | NSn | tapijt | tapestry | 9 | 64% |
| DID-S | TIS | NSn | theelepel | teaspoon | 9 | 64% |
| DID-S | NIS | NSn | tijdperk | era | 9 | 64% |
| DID-S | TIS | NSn | trottoir | pavement | 9 | 64% |
| DID-S | TIS | NSn | zandloper | hourglass | 9 | 64% |
| DID-S | NIS | St | angst | fear | 8 | 57% |
| DID-S | TIS | St | bang | afraid | 8 | 57% |
| DID-S | TIS | St | doorslikken | to swallow | 8 | 57% |
| DID-S | TIS | St | dwang | coercion or force | 8 | 57% |
| DID-S | TIS | St | dwingen | to coerce or to force | 8 | 57% |
| DID-S | NIS | St | onmacht | powerlessness | 8 | 57% |
| DID-S | NIS | St | onrecht | injustice | 8 | 57% |
| DID-S | TIS | NSt | bloedbad | bloodbath | 8 | 57% |
| DID-S | NIS | NSt | doodslag | manslaughter | 8 | 57% |
| DID-S | NIS | NSt | gezwel | tumor | 8 | 57% |
| DID-S | NIS | NSt | kogel | bullet | 8 | 57% |
| DID-S | NIS | NSt | misdrijf | crime | 8 | 57% |
| DID-S | NIS | NSt | omkomen | to perish | 8 | 57% |
| DID-S | NIS | NSt | oorlog | war | 8 | 57% |
| DID-S | NIS | NSt | slet | slut | 8 | 57% |
| DID-S | TIS | NSt | verdrinken | to drown | 8 | 57% |
| DID-S | TIS | NSt | verzuipen | to drown | 8 | 57% |
| DID-S | NIS | NSn | plank | shelf | 8 | 57% |
| DID-S | TIS | NSn | plank | shelf | 8 | 57% |
| DID-S | TIS | NSn | rad | wheel | 8 | 57% |
| DID-S | NIS | NSn | register | register | 8 | 57% |
| DID-S | NIS | NSn | stukadoor | plasterer | 8 | 57% |
| DID-S | NIS | NSn | trede | step | 8 | 57% |
| DID-S | NIS | NSn | zandloper | hourglass | 8 | 57% |
| DID-S | TIS | NSn | zegel | seal | 8 | 57% |
| DID-S | TIS | St | afkeer | aversion | 7 | 50% |
| DID-S | TIS | St | haten | to hate | 7 | 50% |
| DID-S | NIS | St | leugen | lie | 7 | 50% |
| DID-S | TIS | St | lijden | to suffer | 7 | 50% |
| DID-S | TIS | St | man | man | 7 | 50% |
| DID-S | TIS | St | ongewenst | unwanted | 7 | 50% |
| DID-S | TIS | St | onmacht | powerlessness | 7 | 50% |
| DID-S | TIS | St | onrecht | injustice | 7 | 50% |
| DID-S | NIS | St | onzeker | uncertain | 7 | 50% |
| DID-S | TIS | St | pijn | pain | 7 | 50% |
| DID-S | TIS | St | slecht | bad | 7 | 50% |
| DID-S | TIS | St | verlammen | to paralyze | 7 | 50% |
| DID-S | TIS | St | zuigen | to suck | 7 | 50% |
| DID-S | TIS | NSt | aanslag | attack | 7 | 50% |
| DID-S | NIS | NSt | afgrijzen | horror | 7 | 50% |
| DID-S | TIS | NSt | crimineel | criminal | 7 | 50% |
| DID-S | TIS | NSt | gijzeling | kidnapping | 7 | 50% |
| DID-S | NIS | NSt | haten | to hate | 7 | 50% |
| DID-S | NIS | NSt | kanker | cancer | 7 | 50% |
| DID-S | NIS | NSt | moord | murder | 7 | 50% |
| DID-S | NIS | NSt | oplichten | to scam | 7 | 50% |
| DID-S | TIS | NSt | pedofiel | paedophile | 7 | 50% |
| DID-S | TIS | NSt | roofmoord | robbery with murder | 7 | 50% |
| DID-S | NIS | NSt | sadist | sadist | 7 | 50% |
| DID-S | NIS | NSt | steekwond | stab wound | 7 | 50% |
| DID-S | TIS | NSt | verdord | withered | 7 | 50% |
| DID-S | NIS | NSt | verzuipen | to drown | 7 | 50% |
| DID-S | NIS | NSt | walging | disgust | 7 | 50% |
| DID-S | NIS | NSt | wraak | revenge | 7 | 50% |
| DID-S | NIS | NSt | wurgen | to strangle | 7 | 50% |
| DID-S | TIS | NSn | bestek | cutlery | 7 | 50% |
| DID-S | NIS | NSn | cirkel | circle | 7 | 50% |
| DID-S | NIS | NSn | consulaat | consulate | 7 | 50% |
| DID-S | NIS | NSn | dakgoot | gutter | 7 | 50% |
| DID-S | NIS | NSn | deurknop | door knob | 7 | 50% |
| DID-S | TIS | NSn | flacon | bottle or vial | 7 | 50% |
| DID-S | NIS | NSn | kenteken | license plate | 7 | 50% |
| DID-S | NIS | NSn | leuning | railing | 7 | 50% |
| DID-S | NIS | NSn | metselaar | bricklayer | 7 | 50% |
| DID-S | TIS | NSn | metselaar | bricklayer | 7 | 50% |
| DID-S | TIS | NSn | potlood | pencil | 7 | 50% |
| DID-S | TIS | NSn | roeren | to stir | 7 | 50% |
| DID-S | TIS | NSn | schroef | screw | 7 | 50% |
| DID-S | TIS | NSn | stukadoor | plasterer | 7 | 50% |
| DID-S | NIS | NSn | tegel | tile | 7 | 50% |
| DID-S | TIS | NSn | tegel | tile | 7 | 50% |
| DID-S | NIS | NSn | teller | counter | 7 | 50% |
| DID-S | TIS | NSn | trede | step | 7 | 50% |
| DID-S | NIS | NSn | trottoir | pavement | 7 | 50% |
| DID-S | TIS | NSn | uitgever | publisher | 7 | 50% |
| DID-S | TIS | NSn | vierkant | square | 7 | 50% |
| DID-S | TIS | St | afwijzing | rejection | 6 | 43% |
| DID-S | NIS | St | bang | afraid | 6 | 43% |
| DID-S | TIS | St | bedreigen | to threaten | 6 | 43% |
| DID-S | TIS | St | ellende | misery | 6 | 43% |
| DID-S | NIS | St | ergernis | annoyance | 6 | 43% |
| DID-S | NIS | St | falen | to fail | 6 | 43% |
| DID-S | TIS | St | hijgen | to pant | 6 | 43% |
| DID-S | TIS | St | incest | incest | 6 | 43% |
| DID-S | NIS | St | liegen | to lie | 6 | 43% |
| DID-S | TIS | St | likken | to lick | 6 | 43% |
| DID-S | NIS | St | mislukt | failed | 6 | 43% |
| DID-S | NIS | St | ontrouw | unfaithful | 6 | 43% |
| DID-S | TIS | St | pedofiel | paedophile | 6 | 43% |
| DID-S | TIS | St | schoppen | to kick | 6 | 43% |
| DID-S | NIS | St | schuldig | guilty | 6 | 43% |
| DID-S | TIS | St | schuldig | guilty | 6 | 43% |
| DID-S | NIS | St | snauwen | to snarl | 6 | 43% |
| DID-S | TIS | St | stiekem | secretly | 6 | 43% |
| DID-S | TIS | St | vader | father | 6 | 43% |
| DID-S | NIS | St | vies | dirty | 6 | 43% |
| DID-S | NIS | NSt | beroerte | stroke | 6 | 43% |
| DID-S | TIS | NSt | doodslag | manslaughter | 6 | 43% |
| DID-S | NIS | NSt | doodsteek | deathblow | 6 | 43% |
| DID-S | NIS | NSt | dwang | coercion or force | 6 | 43% |
| DID-S | TIS | NSt | folteren | to torture | 6 | 43% |
| DID-S | NIS | NSt | meeloper | opportunist | 6 | 43% |
| DID-S | TIS | NSt | messteek | knife stab | 6 | 43% |
| DID-S | TIS | NSt | miskraam | miscarriage | 6 | 43% |
| DID-S | TIS | NSt | moord | murder | 6 | 43% |
| DID-S | TIS | NSt | omkomen | to perish | 6 | 43% |
| DID-S | TIS | NSt | sadist | sadist | 6 | 43% |
| DID-S | TIS | NSt | slet | slut | 6 | 43% |
| DID-S | NIS | NSt | verlammen | to paralyze | 6 | 43% |
| DID-S | TIS | NSt | verleidster | temptress | 6 | 43% |
| DID-S | TIS | NSt | wanhoop | despair | 6 | 43% |
| DID-S | NIS | NSt | wreedheid | cruelty | 6 | 43% |
| DID-S | TIS | NSn | aanmaken | to prepare | 6 | 43% |
| DID-S | NIS | NSn | bestek | cutlery | 6 | 43% |
| DID-S | TIS | NSn | bladzijde | page | 6 | 43% |
| DID-S | NIS | NSn | blikje | can | 6 | 43% |
| DID-S | TIS | NSn | broer | brother | 6 | 43% |
| DID-S | TIS | NSn | citaat | quote | 6 | 43% |
| DID-S | TIS | NSn | dwerg | dwarf | 6 | 43% |
| DID-S | NIS | NSn | element | element | 6 | 43% |
| DID-S | NIS | NSn | etiket | label | 6 | 43% |
| DID-S | NIS | NSn | firma | firm | 6 | 43% |
| DID-S | TIS | NSn | firma | firm | 6 | 43% |
| DID-S | NIS | NSn | haak | hook | 6 | 43% |
| DID-S | TIS | NSn | haak | hook | 6 | 43% |
| DID-S | NIS | NSn | kelder | basement | 6 | 43% |
| DID-S | NIS | NSn | kozijn | window frame | 6 | 43% |
| DID-S | TIS | NSn | leuning | railing | 6 | 43% |
| DID-S | TIS | NSn | magazijn | warehouse | 6 | 43% |
| DID-S | NIS | NSn | paragraaf | paragraph | 6 | 43% |
| DID-S | TIS | NSn | plafond | ceiling | 6 | 43% |
| DID-S | TIS | NSn | regenton | rain barrel | 6 | 43% |
| DID-S | TIS | NSn | stomerij | drycleaner | 6 | 43% |
| DID-S | NIS | NSn | telegram | telegram | 6 | 43% |
| DID-S | TIS | NSn | teller | counter | 6 | 43% |
| DID-S | NIS | NSn | theelepel | teaspoon | 6 | 43% |
| DID-S | NIS | NSn | touw | rope | 6 | 43% |
| DID-S | NIS | NSn | treden | to step | 6 | 43% |
| DID-S | NIS | NSn | uitgever | publisher | 6 | 43% |
| DID-S | NIS | NSn | zakje | little bag | 6 | 43% |
| DID-S | TIS | St | afblaffen | to bark at | 5 | 36% |
| DID-S | NIS | St | afkraken | to decry | 5 | 36% |
| DID-S | NIS | St | afscheid | goodbye | 5 | 36% |
| DID-S | NIS | St | afsnauwen | to snap at | 5 | 36% |
| DID-S | NIS | St | alleen | alone | 5 | 36% |
| DID-S | TIS | St | angst | fear | 5 | 36% |
| DID-S | NIS | St | bedrieger | deceiver | 5 | 36% |
| DID-S | NIS | St | bedrog | deceit | 5 | 36% |
| DID-S | NIS | St | belazerd | fooled | 5 | 36% |
| DID-S | NIS | St | droefheid | sadness | 5 | 36% |
| DID-S | NIS | St | dwang | coercion or force | 5 | 36% |
| DID-S | NIS | St | dwingen | to coerce or to force | 5 | 36% |
| DID-S | TIS | St | kreunen | to moan | 5 | 36% |
| DID-S | NIS | St | kwetsen | to hurt | 5 | 36% |
| DID-S | TIS | St | kwetsen | to hurt | 5 | 36% |
| DID-S | NIS | St | lafaard | coward | 5 | 36% |
| DID-S | NIS | St | lawaai | noise | 5 | 36% |
| DID-S | TIS | St | leugen | lie | 5 | 36% |
| DID-S | TIS | St | mislukt | failed | 5 | 36% |
| DID-S | TIS | St | opsluiten | to lock up | 5 | 36% |
| DID-S | NIS | St | piekeren | to mull | 5 | 36% |
| DID-S | TIS | St | piemel | willy | 5 | 36% |
| DID-S | TIS | St | stikken | to suffocate | 5 | 36% |
| DID-S | TIS | St | tiran | tyrant | 5 | 36% |
| DID-S | TIS | St | trauma | trauma | 5 | 36% |
| DID-S | NIS | St | verdriet | sadness | 5 | 36% |
| DID-S | TIS | St | vies | dirty | 5 | 36% |
| DID-S | TIS | St | wreedheid | cruelty | 5 | 36% |
| DID-S | TIS | St | wurgen | to strangle | 5 | 36% |
| DID-S | NIS | St | zeuren | to nag | 5 | 36% |
| DID-S | NIS | NSt | aanslag | attack | 5 | 36% |
| DID-S | NIS | NSt | atoombom | nuclear bomb | 5 | 36% |
| DID-S | NIS | NSt | bedreigen | to threaten | 5 | 36% |
| DID-S | NIS | NSt | bedrieger | deceiver | 5 | 36% |
| DID-S | TIS | NSt | beroerte | stroke | 5 | 36% |
| DID-S | NIS | NSt | besmetten | to contaminate | 5 | 36% |
| DID-S | TIS | NSt | besmetten | to contaminate | 5 | 36% |
| DID-S | NIS | NSt | doden | to kill | 5 | 36% |
| DID-S | TIS | NSt | doodsteek | deathblow | 5 | 36% |
| DID-S | NIS | NSt | ellende | misery | 5 | 36% |
| DID-S | TIS | NSt | gezwel | tumor | 5 | 36% |
| DID-S | NIS | NSt | gijzeling | kidnapping | 5 | 36% |
| DID-S | TIS | NSt | hijgen | to pant | 5 | 36% |
| DID-S | TIS | NSt | incest | incest | 5 | 36% |
| DID-S | NIS | NSt | instorten | to collapse | 5 | 36% |
| DID-S | NIS | NSt | kotsen | to vomit | 5 | 36% |
| DID-S | NIS | NSt | lepra | leprosy | 5 | 36% |
| DID-S | TIS | NSt | lepra | leprosy | 5 | 36% |
| DID-S | NIS | NSt | lijden | to suffer | 5 | 36% |
| DID-S | TIS | NSt | martelen | to torture | 5 | 36% |
| DID-S | NIS | NSt | moedeloos | despondent | 5 | 36% |
| DID-S | TIS | NSt | orgie | orgy | 5 | 36% |
| DID-S | NIS | NSt | schoft | bastard | 5 | 36% |
| DID-S | NIS | NSt | stinken | to stink | 5 | 36% |
| DID-S | TIS | NSt | stinken | to stink | 5 | 36% |
| DID-S | TIS | NSt | zeuren | to nag | 5 | 36% |
| DID-S | NIS | NSn | absorptie | absorption | 5 | 36% |
| DID-S | TIS | NSn | cirkel | circle | 5 | 36% |
| DID-S | NIS | NSn | citaat | quote | 5 | 36% |
| DID-S | TIS | NSn | consulaat | consulate | 5 | 36% |
| DID-S | TIS | NSn | dakgoot | gutter | 5 | 36% |
| DID-S | TIS | NSn | deurknop | door knob | 5 | 36% |
| DID-S | NIS | NSn | dwerg | dwarf | 5 | 36% |
| DID-S | NIS | NSn | hertogin | duchess | 5 | 36% |
| DID-S | NIS | NSn | ijzer | iron | 5 | 36% |
| DID-S | TIS | NSn | ivoor | ivory | 5 | 36% |
| DID-S | TIS | NSn | kerk | church | 5 | 36% |
| DID-S | NIS | NSn | middel | middle | 5 | 36% |
| DID-S | NIS | NSn | omroep | broadcasting | 5 | 36% |
| DID-S | TIS | NSn | omroep | broadcasting | 5 | 36% |
| DID-S | TIS | NSn | ontslag | resignation or dismissal | 5 | 36% |
| DID-S | TIS | NSn | paragraaf | paragraph | 5 | 36% |
| DID-S | NIS | NSn | pilaar | pillar | 5 | 36% |
| DID-S | TIS | NSn | pilaar | pillar | 5 | 36% |
| DID-S | NIS | NSn | plafond | ceiling | 5 | 36% |
| DID-S | NIS | NSn | postzegel | stamp | 5 | 36% |
| DID-S | TIS | NSn | postzegel | stamp | 5 | 36% |
| DID-S | NIS | NSn | potlood | pencil | 5 | 36% |
| DID-S | NIS | NSn | rad | wheel | 5 | 36% |
| DID-S | NIS | NSn | schuren | to polish | 5 | 36% |
| DID-S | NIS | NSn | stoelpoot | chair leg | 5 | 36% |
| DID-S | TIS | NSn | takken | branches | 5 | 36% |
| DID-S | TIS | NSn | touw | rope | 5 | 36% |
| DID-S | TIS | NSn | traject | route | 5 | 36% |
| DID-S | TIS | NSn | treden | to step | 5 | 36% |
| DID-S | NIS | NSn | zegel | seal | 5 | 36% |
| DID-S | TIS | NSn | zetel | seat | 5 | 36% |
| DID-S | TIS | St | afgrijzen | horror | 4 | 29% |
| DID-S | NIS | St | afgunst | envy | 4 | 29% |
| DID-S | TIS | St | bedrieger | deceiver | 4 | 29% |
| DID-S | NIS | St | benauwen | to agitate | 4 | 29% |
| DID-S | TIS | St | branden | to burn | 4 | 29% |
| DID-S | NIS | St | droevig | sad | 4 | 29% |
| DID-S | NIS | St | ergeren | to annoy | 4 | 29% |
| DID-S | TIS | St | folteren | to torture | 4 | 29% |
| DID-S | TIS | St | kotsen | to vomit | 4 | 29% |
| DID-S | TIS | St | kreng | bitch | 4 | 29% |
| DID-S | TIS | St | martelen | to torture | 4 | 29% |
| DID-S | TIS | St | mismaakt | deformed | 4 | 29% |
| DID-S | TIS | St | noodkreet | cry for help | 4 | 29% |
| DID-S | NIS | St | ongewenst | unwanted | 4 | 29% |
| DID-S | NIS | St | pijn | pain | 4 | 29% |
| DID-S | NIS | St | ruzie | fight or quarrel | 4 | 29% |
| DID-S | TIS | St | schande | shame | 4 | 29% |
| DID-S | TIS | St | seks | sex | 4 | 29% |
| DID-S | TIS | St | slaaf | slave | 4 | 29% |
| DID-S | TIS | St | snauwen | to snarl | 4 | 29% |
| DID-S | TIS | St | trappen | to kick | 4 | 29% |
| DID-S | NIS | St | uitgescholden | scolded | 4 | 29% |
| DID-S | TIS | St | uitgescholden | scolded | 4 | 29% |
| DID-S | NIS | St | verraad | betrayal | 4 | 29% |
| DID-S | TIS | St | verraad | betrayal | 4 | 29% |
| DID-S | TIS | St | wanhoop | despair | 4 | 29% |
| DID-S | TIS | St | zeer | ache | 4 | 29% |
| DID-S | NIS | NSt | afblaffen | to bark at | 4 | 29% |
| DID-S | NIS | NSt | agressie | aggression | 4 | 29% |
| DID-S | NIS | NSt | armoede | poverty | 4 | 29% |
| DID-S | TIS | NSt | atoombom | nuclear bomb | 4 | 29% |
| DID-S | TIS | NSt | blinddoek | blindfold | 4 | 29% |
| DID-S | TIS | NSt | doden | to kill | 4 | 29% |
| DID-S | TIS | NSt | doodgaan | to die | 4 | 29% |
| DID-S | NIS | NSt | etter | pus | 4 | 29% |
| DID-S | NIS | NSt | hoer | whore | 4 | 29% |
| DID-S | NIS | NSt | kreng | bitch | 4 | 29% |
| DID-S | TIS | NSt | misdaad | crime | 4 | 29% |
| DID-S | TIS | NSt | mislukt | failed | 4 | 29% |
| DID-S | NIS | NSt | mismaakt | deformed | 4 | 29% |
| DID-S | NIS | NSt | noodkreet | cry for help | 4 | 29% |
| DID-S | TIS | NSt | oplichten | to scam | 4 | 29% |
| DID-S | NIS | NSt | orgie | orgy | 4 | 29% |
| DID-S | NIS | NSt | roofmoord | robbery with murder | 4 | 29% |
| DID-S | NIS | NSt | ruzie | fight or quarrel | 4 | 29% |
| DID-S | TIS | NSt | slijmen | suck up to | 4 | 29% |
| DID-S | TIS | NSt | steekwond | stab wound | 4 | 29% |
| DID-S | NIS | NSt | stikken | to suffocate | 4 | 29% |
| DID-S | NIS | NSt | tiran | tyrant | 4 | 29% |
| DID-S | NIS | NSt | treiteren | to harass | 4 | 29% |
| DID-S | NIS | NSt | uitjouwen | to boo | 4 | 29% |
| DID-S | TIS | NSt | verlammen | to paralyze | 4 | 29% |
| DID-S | NIS | NSt | vernielen | to vandalize | 4 | 29% |
| DID-S | NIS | NSt | wanhoop | despair | 4 | 29% |
| DID-S | NIS | NSn | aanmaken | to prepare | 4 | 29% |
| DID-S | NIS | NSn | blinddoek | blindfold | 4 | 29% |
| DID-S | TIS | NSn | chauffeur | driver | 4 | 29% |
| DID-S | NIS | NSn | document | document | 4 | 29% |
| DID-S | TIS | NSn | dozijn | dozen | 4 | 29% |
| DID-S | NIS | NSn | elleboog | elbow | 4 | 29% |
| DID-S | TIS | NSn | etiket | label | 4 | 29% |
| DID-S | NIS | NSn | flacon | bottle or vial | 4 | 29% |
| DID-S | NIS | NSn | gaatje | little hole | 4 | 29% |
| DID-S | TIS | NSn | gaatje | little hole | 4 | 29% |
| DID-S | NIS | NSn | gebouw | building | 4 | 29% |
| DID-S | TIS | NSn | gebouw | building | 4 | 29% |
| DID-S | NIS | NSn | hagedis | lizard | 4 | 29% |
| DID-S | NIS | NSn | haken | hooks | 4 | 29% |
| DID-S | NIS | NSn | handen | hands | 4 | 29% |
| DID-S | TIS | NSn | kader | framework | 4 | 29% |
| DID-S | TIS | NSn | kast | closet | 4 | 29% |
| DID-S | TIS | NSn | kenteken | license plate | 4 | 29% |
| DID-S | NIS | NSn | kerk | church | 4 | 29% |
| DID-S | TIS | NSn | metaal | metal | 4 | 29% |
| DID-S | TIS | NSn | ogen | eyes | 4 | 29% |
| DID-S | NIS | NSn | regenton | rain barrel | 4 | 29% |
| DID-S | NIS | NSn | roeren | to stir | 4 | 29% |
| DID-S | TIS | NSn | spoelen | to flush or to rinse | 4 | 29% |
| DID-S | TIS | NSn | steil | steep | 4 | 29% |
| DID-S | NIS | NSn | stoep | sidewalk | 4 | 29% |
| DID-S | TIS | NSn | stoep | sidewalk | 4 | 29% |
| DID-S | NIS | NSn | stomerij | drycleaner | 4 | 29% |
| DID-S | NIS | NSn | takken | branches | 4 | 29% |
| DID-S | NIS | NSn | tapijt | tapestry | 4 | 29% |
| DID-S | NIS | NSn | traject | route | 4 | 29% |
| DID-S | NIS | NSn | uitkleden | to undress | 4 | 29% |
| DID-S | NIS | NSn | verdord | withered | 4 | 29% |
| DID-S | NIS | NSn | versie | version | 4 | 29% |
| DID-S | NIS | NSn | vierkant | square | 4 | 29% |
| DID-S | NIS | NSn | vreemde | stranger | 4 | 29% |
| DID-S | TIS | NSn | zakje | little bag | 4 | 29% |
| DID-S | NIS | NSn | zetel | seat | 4 | 29% |
| DID-S | NIS | St | afgrijzen | horror | 3 | 21% |
| DID-S | NIS | St | afkeer | aversion | 3 | 21% |
| DID-S | TIS | St | afschuw | revulsion | 3 | 21% |
| DID-S | TIS | St | afsnauwen | to snap at | 3 | 21% |
| DID-S | TIS | St | alleen | alone | 3 | 21% |
| DID-S | TIS | St | bed | bed | 3 | 21% |
| DID-S | TIS | St | benauwen | to agitate | 3 | 21% |
| DID-S | TIS | St | besmetten | to contaminate | 3 | 21% |
| DID-S | TIS | St | bloot | naked | 3 | 21% |
| DID-S | TIS | St | chanteren | to blackmail | 3 | 21% |
| DID-S | NIS | St | conflict | conflict | 3 | 21% |
| DID-S | TIS | St | conflict | conflict | 3 | 21% |
| DID-S | NIS | St | domkop | idiot | 3 | 21% |
| DID-S | TIS | St | domkop | idiot | 3 | 21% |
| DID-S | NIS | St | dreigen | to threaten | 3 | 21% |
| DID-S | TIS | St | dreigen | to threaten | 3 | 21% |
| DID-S | TIS | St | droefheid | sadness | 3 | 21% |
| DID-S | TIS | St | falen | to fail | 3 | 21% |
| DID-S | NIS | St | gezwel | tumor | 3 | 21% |
| DID-S | TIS | St | instorten | to collapse | 3 | 21% |
| DID-S | TIS | St | knijpen | to pinch | 3 | 21% |
| DID-S | NIS | St | kotsen | to vomit | 3 | 21% |
| DID-S | NIS | St | kou | cold | 3 | 21% |
| DID-S | TIS | St | kuthoer | pussy whore | 3 | 21% |
| DID-S | TIS | St | lafaard | coward | 3 | 21% |
| DID-S | TIS | St | meester | master | 3 | 21% |
| DID-S | TIS | St | metaal | metal | 3 | 21% |
| DID-S | NIS | St | misleiden | to deceive | 3 | 21% |
| DID-S | NIS | St | moedeloos | despondent | 3 | 21% |
| DID-S | TIS | St | mond | mouth | 3 | 21% |
| DID-S | NIS | St | omkomen | to perish | 3 | 21% |
| DID-S | NIS | St | ongeluk | accident | 3 | 21% |
| DID-S | TIS | St | onzeker | uncertain | 3 | 21% |
| DID-S | NIS | St | schande | shame | 3 | 21% |
| DID-S | TIS | St | slet | slut | 3 | 21% |
| DID-S | TIS | St | snijden | to cut | 3 | 21% |
| DID-S | NIS | St | stank | stench | 3 | 21% |
| DID-S | TIS | St | stank | stench | 3 | 21% |
| DID-S | NIS | St | stikken | to suffocate | 3 | 21% |
| DID-S | NIS | St | tegenslag | setback | 3 | 21% |
| DID-S | NIS | St | tiran | tyrant | 3 | 21% |
| DID-S | TIS | St | treiteren | to harass | 3 | 21% |
| DID-S | TIS | St | uitkleden | to undress | 3 | 21% |
| DID-S | TIS | St | uitlachen | to laugh at | 3 | 21% |
| DID-S | NIS | St | verbranden | to burn | 3 | 21% |
| DID-S | TIS | St | verdriet | sadness | 3 | 21% |
| DID-S | NIS | St | vreemde | stranger | 3 | 21% |
| DID-S | NIS | St | walging | disgust | 3 | 21% |
| DID-S | NIS | St | wanhoop | despair | 3 | 21% |
| DID-S | TIS | St | wanhopen | to despair | 3 | 21% |
| DID-S | TIS | NSt | afgrijzen | horror | 3 | 21% |
| DID-S | TIS | NSt | afhakken | to chop off | 3 | 21% |
| DID-S | NIS | NSt | afkraken | to decry | 3 | 21% |
| DID-S | TIS | NSt | afsnauwen | to snap at | 3 | 21% |
| DID-S | TIS | NSt | angst | fear | 3 | 21% |
| DID-S | TIS | NSt | armoede | poverty | 3 | 21% |
| DID-S | NIS | NSt | belazerd | fooled | 3 | 21% |
| DID-S | NIS | NSt | bordeel | brothel | 3 | 21% |
| DID-S | TIS | NSt | bordeel | brothel | 3 | 21% |
| DID-S | NIS | NSt | chanteren | to blackmail | 3 | 21% |
| DID-S | TIS | NSt | depressie | depression | 3 | 21% |
| DID-S | TIS | NSt | diefstal | theft | 3 | 21% |
| DID-S | NIS | NSt | doodgaan | to die | 3 | 21% |
| DID-S | NIS | NSt | dwingen | to coerce or to force | 3 | 21% |
| DID-S | TIS | NSt | ellende | misery | 3 | 21% |
| DID-S | TIS | NSt | ergernis | annoyance | 3 | 21% |
| DID-S | TIS | NSt | etter | pus | 3 | 21% |
| DID-S | TIS | NSt | falen | to fail | 3 | 21% |
| DID-S | NIS | NSt | hysterie | hysteria | 3 | 21% |
| DID-S | TIS | NSt | hysterie | hysteria | 3 | 21% |
| DID-S | NIS | NSt | inbraak | burglary | 3 | 21% |
| DID-S | TIS | NSt | inbraak | burglary | 3 | 21% |
| DID-S | TIS | NSt | instorten | to collapse | 3 | 21% |
| DID-S | TIS | NSt | kelder | basement | 3 | 21% |
| DID-S | TIS | NSt | kogel | bullet | 3 | 21% |
| DID-S | NIS | NSt | krenken | to hurt | 3 | 21% |
| DID-S | NIS | NSt | kreunen | to moan | 3 | 21% |
| DID-S | NIS | NSt | kuthoer | pussy whore | 3 | 21% |
| DID-S | TIS | NSt | lafaard | coward | 3 | 21% |
| DID-S | TIS | NSt | man | man | 3 | 21% |
| DID-S | TIS | NSt | meeloper | opportunist | 3 | 21% |
| DID-S | NIS | NSt | misdaad | crime | 3 | 21% |
| DID-S | TIS | NSt | misdrijf | crime | 3 | 21% |
| DID-S | NIS | NSt | misleiden | to deceive | 3 | 21% |
| DID-S | TIS | NSt | moedeloos | despondent | 3 | 21% |
| DID-S | TIS | NSt | ontrouw | unfaithful | 3 | 21% |
| DID-S | NIS | NSt | opsluiten | to lock up | 3 | 21% |
| DID-S | NIS | NSt | slaaf | slave | 3 | 21% |
| DID-S | NIS | NSt | slijmen | suck up to | 3 | 21% |
| DID-S | NIS | NSt | trauma | trauma | 3 | 21% |
| DID-S | TIS | NSt | trauma | trauma | 3 | 21% |
| DID-S | TIS | NSt | verbranden | to burn | 3 | 21% |
| DID-S | NIS | NSt | verleidster | temptress | 3 | 21% |
| DID-S | NIS | NSt | verraad | betrayal | 3 | 21% |
| DID-S | TIS | NSt | wanhopen | to despair | 3 | 21% |
| DID-S | TIS | NSt | wreedheid | cruelty | 3 | 21% |
| DID-S | TIS | NSt | wurgen | to strangle | 3 | 21% |
| DID-S | NIS | NSt | zeuren | to nag | 3 | 21% |
| DID-S | NIS | NSt | zondebok | scapegoat | 3 | 21% |
| DID-S | TIS | NSn | absorptie | absorption | 3 | 21% |
| DID-S | TIS | NSn | armoede | poverty | 3 | 21% |
| DID-S | TIS | NSn | atoombom | nuclear bomb | 3 | 21% |
| DID-S | TIS | NSn | begraven | to bury | 3 | 21% |
| DID-S | TIS | NSn | beroerte | stroke | 3 | 21% |
| DID-S | NIS | NSn | bloot | naked | 3 | 21% |
| DID-S | NIS | NSn | dokter | (general practice) doctor | 3 | 21% |
| DID-S | NIS | NSn | dozijn | dozen | 3 | 21% |
| DID-S | TIS | NSn | element | element | 3 | 21% |
| DID-S | NIS | NSn | geit | goat | 3 | 21% |
| DID-S | TIS | NSn | huisdier | pet | 3 | 21% |
| DID-S | NIS | NSn | kader | framework | 3 | 21% |
| DID-S | NIS | NSn | kast | closet | 3 | 21% |
| DID-S | NIS | NSn | kogel | bullet | 3 | 21% |
| DID-S | TIS | NSn | kozijn | window frame | 3 | 21% |
| DID-S | NIS | NSn | krabben | to scratch | 3 | 21% |
| DID-S | TIS | NSn | krabben | to scratch | 3 | 21% |
| DID-S | NIS | NSn | mes | knife | 3 | 21% |
| DID-S | NIS | NSn | octaaf | octave | 3 | 21% |
| DID-S | TIS | NSn | ongeval | accident | 3 | 21% |
| DID-S | TIS | NSn | pasen | Easter | 3 | 21% |
| DID-S | NIS | NSn | razernij | fury | 3 | 21% |
| DID-S | TIS | NSn | register | register | 3 | 21% |
| DID-S | TIS | NSn | schuren | to polish | 3 | 21% |
| DID-S | NIS | NSn | snijden | to cut | 3 | 21% |
| DID-S | NIS | NSn | steil | steep | 3 | 21% |
| DID-S | TIS | NSn | stoelpoot | chair leg | 3 | 21% |
| DID-S | TIS | NSn | versie | version | 3 | 21% |
| DID-S | TIS | NSn | vreemde | stranger | 3 | 21% |
| DID-S | NIS | NSn | vuur | fire | 3 | 21% |
| DID-S | NIS | St | achterlaten | leave behind | 2 | 14% |
| DID-S | TIS | St | afgunst | envy | 2 | 14% |
| DID-S | NIS | St | afschuw | revulsion | 2 | 14% |
| DID-S | NIS | St | agressie | aggression | 2 | 14% |
| DID-S | TIS | St | agressie | aggression | 2 | 14% |
| DID-S | NIS | St | atoombom | nuclear bomb | 2 | 14% |
| DID-S | NIS | St | bedreigen | to threaten | 2 | 14% |
| DID-S | NIS | St | begraven | to bury | 2 | 14% |
| DID-S | NIS | St | beklemmen | to oppress | 2 | 14% |
| DID-S | TIS | St | belazerd | fooled | 2 | 14% |
| DID-S | NIS | St | beroerte | stroke | 2 | 14% |
| DID-S | NIS | St | besmetten | to contaminate | 2 | 14% |
| DID-S | TIS | St | blinddoek | blindfold | 2 | 14% |
| DID-S | TIS | St | bloedbad | bloodbath | 2 | 14% |
| DID-S | NIS | St | boos | angry | 2 | 14% |
| DID-S | TIS | St | boos | angry | 2 | 14% |
| DID-S | NIS | St | branden | to burn | 2 | 14% |
| DID-S | TIS | St | buurman | neighbor | 2 | 14% |
| DID-S | NIS | St | chanteren | to blackmail | 2 | 14% |
| DID-S | NIS | St | doodgaan | to die | 2 | 14% |
| DID-S | NIS | St | dubbel | double | 2 | 14% |
| DID-S | TIS | St | ergeren | to annoy | 2 | 14% |
| DID-S | NIS | St | getreiter | harassment | 2 | 14% |
| DID-S | TIS | St | getreiter | harassment | 2 | 14% |
| DID-S | NIS | St | geweld | violence | 2 | 14% |
| DID-S | NIS | St | hoer | whore | 2 | 14% |
| DID-S | TIS | St | hoer | whore | 2 | 14% |
| DID-S | NIS | St | kanker | cancer | 2 | 14% |
| DID-S | NIS | St | kelder | basement | 2 | 14% |
| DID-S | NIS | St | kreng | bitch | 2 | 14% |
| DID-S | NIS | St | krenken | to hurt | 2 | 14% |
| DID-S | TIS | St | liegen | to lie | 2 | 14% |
| DID-S | NIS | St | masker | mask | 2 | 14% |
| DID-S | TIS | St | misdrijf | crime | 2 | 14% |
| DID-S | TIS | St | moeder | mother | 2 | 14% |
| DID-S | NIS | St | ontslag | resignation or dismissal | 2 | 14% |
| DID-S | TIS | St | orgie | orgy | 2 | 14% |
| DID-S | NIS | St | pedofiel | paedophile | 2 | 14% |
| DID-S | TIS | St | piekeren | to mull | 2 | 14% |
| DID-S | TIS | St | plafond | ceiling | 2 | 14% |
| DID-S | NIS | St | regel | rule | 2 | 14% |
| DID-S | TIS | St | regel | rule | 2 | 14% |
| DID-S | TIS | St | ruzie | fight or quarrel | 2 | 14% |
| DID-S | TIS | St | sadist | sadist | 2 | 14% |
| DID-S | NIS | St | schaden | to damage | 2 | 14% |
| DID-S | NIS | St | schoft | bastard | 2 | 14% |
| DID-S | TIS | St | schoft | bastard | 2 | 14% |
| DID-S | NIS | St | schoppen | to kick | 2 | 14% |
| DID-S | NIS | St | slaaf | slave | 2 | 14% |
| DID-S | NIS | St | slecht | bad | 2 | 14% |
| DID-S | NIS | St | slet | slut | 2 | 14% |
| DID-S | TIS | St | steekwond | stab wound | 2 | 14% |
| DID-S | NIS | St | stinken | to stink | 2 | 14% |
| DID-S | TIS | St | stinken | to stink | 2 | 14% |
| DID-S | NIS | St | treiteren | to harass | 2 | 14% |
| DID-S | NIS | St | uitjouwen | to boo | 2 | 14% |
| DID-S | TIS | St | uitjouwen | to boo | 2 | 14% |
| DID-S | NIS | St | uitlachen | to laugh at | 2 | 14% |
| DID-S | NIS | St | vastbinden | to tie | 2 | 14% |
| DID-S | NIS | St | verlammen | to paralyze | 2 | 14% |
| DID-S | NIS | St | vernielen | to vandalize | 2 | 14% |
| DID-S | NIS | St | wanhopen | to despair | 2 | 14% |
| DID-S | TIS | St | wraak | revenge | 2 | 14% |
| DID-S | NIS | St | wreedheid | cruelty | 2 | 14% |
| DID-S | NIS | St | wurgen | to strangle | 2 | 14% |
| DID-S | NIS | St | zeer | ache | 2 | 14% |
| DID-S | TIS | St | zeuren | to nag | 2 | 14% |
| DID-S | NIS | NSt | achterlaten | leave behind | 2 | 14% |
| DID-S | TIS | NSt | afkraken | to decry | 2 | 14% |
| DID-S | NIS | NSt | afsnauwen | to snap at | 2 | 14% |
| DID-S | TIS | NSt | agressie | aggression | 2 | 14% |
| DID-S | NIS | NSt | angst | fear | 2 | 14% |
| DID-S | TIS | NSt | baby | baby | 2 | 14% |
| DID-S | TIS | NSt | bang | afraid | 2 | 14% |
| DID-S | NIS | NSt | beklemmen | to oppress | 2 | 14% |
| DID-S | TIS | NSt | belazerd | fooled | 2 | 14% |
| DID-S | TIS | NSt | boos | angry | 2 | 14% |
| DID-S | TIS | NSt | buurman | neighbor | 2 | 14% |
| DID-S | NIS | NSt | conflict | conflict | 2 | 14% |
| DID-S | TIS | NSt | consulaat | consulate | 2 | 14% |
| DID-S | NIS | NSt | diefstal | theft | 2 | 14% |
| DID-S | TIS | NSt | dokter | (general practice) doctor | 2 | 14% |
| DID-S | NIS | NSt | domkop | idiot | 2 | 14% |
| DID-S | TIS | NSt | droefheid | sadness | 2 | 14% |
| DID-S | TIS | NSt | droevig | sad | 2 | 14% |
| DID-S | TIS | NSt | dwerg | dwarf | 2 | 14% |
| DID-S | TIS | NSt | ergeren | to annoy | 2 | 14% |
| DID-S | NIS | NSt | ergernis | annoyance | 2 | 14% |
| DID-S | NIS | NSt | falen | to fail | 2 | 14% |
| DID-S | NIS | NSt | getreiter | harassment | 2 | 14% |
| DID-S | TIS | NSt | haak | hook | 2 | 14% |
| DID-S | TIS | NSt | hertogin | duchess | 2 | 14% |
| DID-S | TIS | NSt | hoer | whore | 2 | 14% |
| DID-S | TIS | NSt | ijzer | iron | 2 | 14% |
| DID-S | TIS | NSt | knijpen | to pinch | 2 | 14% |
| DID-S | NIS | NSt | krabben | to scratch | 2 | 14% |
| DID-S | TIS | NSt | kreunen | to moan | 2 | 14% |
| DID-S | TIS | NSt | kuthoer | pussy whore | 2 | 14% |
| DID-S | TIS | NSt | kwetsen | to hurt | 2 | 14% |
| DID-S | TIS | NSt | legpuzzel | jigsaw puzzle | 2 | 14% |
| DID-S | TIS | NSt | liegen | to lie | 2 | 14% |
| DID-S | TIS | NSt | masker | mask | 2 | 14% |
| DID-S | NIS | NSt | misvormen | to deform | 2 | 14% |
| DID-S | TIS | NSt | omroep | broadcasting | 2 | 14% |
| DID-S | NIS | NSt | ongeluk | accident | 2 | 14% |
| DID-S | TIS | NSt | ongeluk | accident | 2 | 14% |
| DID-S | NIS | NSt | ongewenst | unwanted | 2 | 14% |
| DID-S | NIS | NSt | onmacht | powerlessness | 2 | 14% |
| DID-S | TIS | NSt | onmacht | powerlessness | 2 | 14% |
| DID-S | NIS | NSt | ontrouw | unfaithful | 2 | 14% |
| DID-S | TIS | NSt | onzeker | uncertain | 2 | 14% |
| DID-S | TIS | NSt | opsluiten | to lock up | 2 | 14% |
| DID-S | TIS | NSt | pasen | Easter | 2 | 14% |
| DID-S | NIS | NSt | razernij | fury | 2 | 14% |
| DID-S | TIS | NSt | schoft | bastard | 2 | 14% |
| DID-S | TIS | NSt | schuren | to polish | 2 | 14% |
| DID-S | NIS | NSt | seks | sex | 2 | 14% |
| DID-S | TIS | NSt | seks | sex | 2 | 14% |
| DID-S | TIS | NSt | slaaf | slave | 2 | 14% |
| DID-S | NIS | NSt | snauwen | to snarl | 2 | 14% |
| DID-S | NIS | NSt | snijden | to cut | 2 | 14% |
| DID-S | TIS | NSt | stank | stench | 2 | 14% |
| DID-S | TIS | NSt | stellen | to set | 2 | 14% |
| DID-S | TIS | NSt | sterven | to die | 2 | 14% |
| DID-S | TIS | NSt | tegenslag | setback | 2 | 14% |
| DID-S | TIS | NSt | tiran | tyrant | 2 | 14% |
| DID-S | TIS | NSt | touw | rope | 2 | 14% |
| DID-S | TIS | NSt | treiteren | to harass | 2 | 14% |
| DID-S | TIS | NSt | uitjouwen | to boo | 2 | 14% |
| DID-S | TIS | NSt | uitkleden | to undress | 2 | 14% |
| DID-S | NIS | NSt | uitlachen | to laugh at | 2 | 14% |
| DID-S | NIS | NSt | verdord | withered | 2 | 14% |
| DID-S | TIS | NSt | verdriet | sadness | 2 | 14% |
| DID-S | TIS | NSt | verkrachting | rape | 2 | 14% |
| DID-S | NIS | NSt | wanhopen | to despair | 2 | 14% |
| DID-S | TIS | NSt | zuigen | to suck | 2 | 14% |
| DID-S | NIS | NSn | aanslag | attack | 2 | 14% |
| DID-S | NIS | NSn | beklemmen | to oppress | 2 | 14% |
| DID-S | NIS | NSn | bladzijde | page | 2 | 14% |
| DID-S | TIS | NSn | blikje | can | 2 | 14% |
| DID-S | TIS | NSn | bloot | naked | 2 | 14% |
| DID-S | NIS | NSn | broer | brother | 2 | 14% |
| DID-S | NIS | NSn | chanteren | to blackmail | 2 | 14% |
| DID-S | NIS | NSn | chauffeur | driver | 2 | 14% |
| DID-S | TIS | NSn | document | document | 2 | 14% |
| DID-S | TIS | NSn | dokter | (general practice) doctor | 2 | 14% |
| DID-S | NIS | NSn | doorslikken | to swallow | 2 | 14% |
| DID-S | NIS | NSn | embryo | embryo | 2 | 14% |
| DID-S | TIS | NSn | embryo | embryo | 2 | 14% |
| DID-S | TIS | NSn | geit | goat | 2 | 14% |
| DID-S | TIS | NSn | gijzeling | kidnapping | 2 | 14% |
| DID-S | TIS | NSn | hertogin | duchess | 2 | 14% |
| DID-S | NIS | NSn | hijgen | to pant | 2 | 14% |
| DID-S | NIS | NSn | huisdier | pet | 2 | 14% |
| DID-S | TIS | NSn | ijzer | iron | 2 | 14% |
| DID-S | NIS | NSn | ivoor | ivory | 2 | 14% |
| DID-S | TIS | NSn | juni | June | 2 | 14% |
| DID-S | NIS | NSn | knijpen | to pinch | 2 | 14% |
| DID-S | TIS | NSn | kogel | bullet | 2 | 14% |
| DID-S | TIS | NSn | kou | cold | 2 | 14% |
| DID-S | NIS | NSn | laden | to load | 2 | 14% |
| DID-S | TIS | NSn | laden | to load | 2 | 14% |
| DID-S | TIS | NSn | lawaai | noise | 2 | 14% |
| DID-S | NIS | NSn | legpuzzel | jigsaw puzzle | 2 | 14% |
| DID-S | NIS | NSn | likken | to lick | 2 | 14% |
| DID-S | TIS | NSn | likken | to lick | 2 | 14% |
| DID-S | NIS | NSn | magazijn | warehouse | 2 | 14% |
| DID-S | TIS | NSn | meester | master | 2 | 14% |
| DID-S | NIS | NSn | metaal | metal | 2 | 14% |
| DID-S | NIS | NSn | misvormen | to deform | 2 | 14% |
| DID-S | TIS | NSn | mond | mouth | 2 | 14% |
| DID-S | TIS | NSn | moord | murder | 2 | 14% |
| DID-S | NIS | NSn | ogen | eyes | 2 | 14% |
| DID-S | TIS | NSn | ontrouw | unfaithful | 2 | 14% |
| DID-S | TIS | NSn | oom | uncle | 2 | 14% |
| DID-S | NIS | NSn | pasen | Easter | 2 | 14% |
| DID-S | NIS | NSn | piemel | willy | 2 | 14% |
| DID-S | NIS | NSn | schaden | to damage | 2 | 14% |
| DID-S | TIS | NSn | slijmen | suck up to | 2 | 14% |
| DID-S | NIS | NSn | spoelen | to flush or to rinse | 2 | 14% |
| DID-S | TIS | NSn | stank | stench | 2 | 14% |
| DID-S | NIS | NSn | stellen | to set | 2 | 14% |
| DID-S | TIS | NSn | stellen | to set | 2 | 14% |
| DID-S | TIS | NSn | sterven | to die | 2 | 14% |
| DID-S | NIS | NSn | stoel | chair | 2 | 14% |
| DID-S | TIS | NSn | stoel | chair | 2 | 14% |
| DID-S | TIS | NSn | uitlachen | to laugh at | 2 | 14% |
| DID-S | NIS | NSn | verbranden | to burn | 2 | 14% |
| DID-S | TIS | NSn | verdord | withered | 2 | 14% |
| DID-S | NIS | NSn | vergroten | enlarge | 2 | 14% |
| DID-S | NIS | NSn | zuigen | to suck | 2 | 14% |
| DID-S | NIS | St | aanslag | attack | 1 | 7% |
| DID-S | TIS | St | aanslag | attack | 1 | 7% |
| DID-S | NIS | St | afblaffen | to bark at | 1 | 7% |
| DID-S | TIS | St | afkraken | to decry | 1 | 7% |
| DID-S | TIS | St | armoede | poverty | 1 | 7% |
| DID-S | TIS | St | baby | baby | 1 | 7% |
| DID-S | TIS | St | bad | bath | 1 | 7% |
| DID-S | TIS | St | bedrog | deceit | 1 | 7% |
| DID-S | NIS | St | bladzijde | page | 1 | 7% |
| DID-S | NIS | St | bloedbad | bloodbath | 1 | 7% |
| DID-S | NIS | St | bloot | naked | 1 | 7% |
| DID-S | NIS | St | bordeel | brothel | 1 | 7% |
| DID-S | NIS | St | buurman | neighbor | 1 | 7% |
| DID-S | TIS | St | cirkel | circle | 1 | 7% |
| DID-S | NIS | St | crimineel | criminal | 1 | 7% |
| DID-S | TIS | St | dakgoot | gutter | 1 | 7% |
| DID-S | NIS | St | depressie | depression | 1 | 7% |
| DID-S | NIS | St | diefstal | theft | 1 | 7% |
| DID-S | NIS | St | doden | to kill | 1 | 7% |
| DID-S | TIS | St | dokter | (general practice) doctor | 1 | 7% |
| DID-S | TIS | St | doodslag | manslaughter | 1 | 7% |
| DID-S | NIS | St | doodsteek | deathblow | 1 | 7% |
| DID-S | NIS | St | doorslikken | to swallow | 1 | 7% |
| DID-S | TIS | St | droevig | sad | 1 | 7% |
| DID-S | TIS | St | dubbel | double | 1 | 7% |
| DID-S | NIS | St | ellende | misery | 1 | 7% |
| DID-S | TIS | St | embryo | embryo | 1 | 7% |
| DID-S | NIS | St | etiket | label | 1 | 7% |
| DID-S | NIS | St | etter | pus | 1 | 7% |
| DID-S | TIS | St | gaatje | little hole | 1 | 7% |
| DID-S | TIS | St | gezwel | tumor | 1 | 7% |
| DID-S | TIS | St | gijzeling | kidnapping | 1 | 7% |
| DID-S | TIS | St | handen | hands | 1 | 7% |
| DID-S | NIS | St | haten | to hate | 1 | 7% |
| DID-S | TIS | St | hysterie | hysteria | 1 | 7% |
| DID-S | NIS | St | instorten | to collapse | 1 | 7% |
| DID-S | TIS | St | kader | framework | 1 | 7% |
| DID-S | TIS | St | kelder | basement | 1 | 7% |
| DID-S | NIS | St | knijpen | to pinch | 1 | 7% |
| DID-S | TIS | St | kou | cold | 1 | 7% |
| DID-S | NIS | St | krabben | to scratch | 1 | 7% |
| DID-S | NIS | St | kreunen | to moan | 1 | 7% |
| DID-S | NIS | St | lijden | to suffer | 1 | 7% |
| DID-S | NIS | St | man | man | 1 | 7% |
| DID-S | NIS | St | martelen | to torture | 1 | 7% |
| DID-S | NIS | St | meeloper | opportunist | 1 | 7% |
| DID-S | TIS | St | mes | knife | 1 | 7% |
| DID-S | NIS | St | messteek | knife stab | 1 | 7% |
| DID-S | TIS | St | middel | middle | 1 | 7% |
| DID-S | NIS | St | misdaad | crime | 1 | 7% |
| DID-S | TIS | St | misdaad | crime | 1 | 7% |
| DID-S | NIS | St | miskraam | miscarriage | 1 | 7% |
| DID-S | TIS | St | miskraam | miscarriage | 1 | 7% |
| DID-S | TIS | St | misleiden | to deceive | 1 | 7% |
| DID-S | TIS | St | misvormen | to deform | 1 | 7% |
| DID-S | NIS | St | moeder | mother | 1 | 7% |
| DID-S | NIS | St | mond | mouth | 1 | 7% |
| DID-S | NIS | St | moord | murder | 1 | 7% |
| DID-S | NIS | St | noodkreet | cry for help | 1 | 7% |
| DID-S | NIS | St | ongeval | accident | 1 | 7% |
| DID-S | TIS | St | ontrouw | unfaithful | 1 | 7% |
| DID-S | NIS | St | oom | uncle | 1 | 7% |
| DID-S | TIS | St | oom | uncle | 1 | 7% |
| DID-S | NIS | St | oorlog | war | 1 | 7% |
| DID-S | NIS | St | oplichten | to scam | 1 | 7% |
| DID-S | NIS | St | opsluiten | to lock up | 1 | 7% |
| DID-S | NIS | St | piemel | willy | 1 | 7% |
| DID-S | NIS | St | plank | shelf | 1 | 7% |
| DID-S | TIS | St | razernij | fury | 1 | 7% |
| DID-S | NIS | St | sadist | sadist | 1 | 7% |
| DID-S | TIS | St | schaden | to damage | 1 | 7% |
| DID-S | NIS | St | slijmen | suck up to | 1 | 7% |
| DID-S | NIS | St | spoelen | to flush or to rinse | 1 | 7% |
| DID-S | NIS | St | steil | steep | 1 | 7% |
| DID-S | NIS | St | sterven | to die | 1 | 7% |
| DID-S | NIS | St | stiekem | secretly | 1 | 7% |
| DID-S | NIS | St | stoep | sidewalk | 1 | 7% |
| DID-S | TIS | St | tegenslag | setback | 1 | 7% |
| DID-S | TIS | St | touw | rope | 1 | 7% |
| DID-S | TIS | St | treden | to step | 1 | 7% |
| DID-S | NIS | St | trottoir | pavement | 1 | 7% |
| DID-S | NIS | St | uitkleden | to undress | 1 | 7% |
| DID-S | TIS | St | vastbinden | to tie | 1 | 7% |
| DID-S | NIS | St | verdrinken | to drown | 1 | 7% |
| DID-S | TIS | St | verdrinken | to drown | 1 | 7% |
| DID-S | NIS | St | verleidster | temptress | 1 | 7% |
| DID-S | TIS | St | vernielen | to vandalize | 1 | 7% |
| DID-S | NIS | St | versie | version | 1 | 7% |
| DID-S | TIS | St | versie | version | 1 | 7% |
| DID-S | NIS | St | verzuipen | to drown | 1 | 7% |
| DID-S | TIS | St | verzuipen | to drown | 1 | 7% |
| DID-S | TIS | St | vreemde | stranger | 1 | 7% |
| DID-S | NIS | St | vuur | fire | 1 | 7% |
| DID-S | TIS | St | vuur | fire | 1 | 7% |
| DID-S | NIS | St | woest | enraged | 1 | 7% |
| DID-S | TIS | St | woest | enraged | 1 | 7% |
| DID-S | TIS | St | zakje | little bag | 1 | 7% |
| DID-S | NIS | St | zondebok | scapegoat | 1 | 7% |
| DID-S | TIS | St | zondebok | scapegoat | 1 | 7% |
| DID-S | TIS | NSt | achterlaten | leave behind | 1 | 7% |
| DID-S | TIS | NSt | afblaffen | to bark at | 1 | 7% |
| DID-S | NIS | NSt | afgunst | envy | 1 | 7% |
| DID-S | TIS | NSt | afgunst | envy | 1 | 7% |
| DID-S | TIS | NSt | afkeer | aversion | 1 | 7% |
| DID-S | NIS | NSt | afschuw | revulsion | 1 | 7% |
| DID-S | NIS | NSt | afwijzing | rejection | 1 | 7% |
| DID-S | TIS | NSt | afwijzing | rejection | 1 | 7% |
| DID-S | NIS | NSt | baby | baby | 1 | 7% |
| DID-S | TIS | NSt | bad | bath | 1 | 7% |
| DID-S | NIS | NSt | bang | afraid | 1 | 7% |
| DID-S | TIS | NSt | bedreigen | to threaten | 1 | 7% |
| DID-S | TIS | NSt | bedrieger | deceiver | 1 | 7% |
| DID-S | TIS | NSt | begraven | to bury | 1 | 7% |
| DID-S | TIS | NSt | benauwen | to agitate | 1 | 7% |
| DID-S | NIS | NSt | blinddoek | blindfold | 1 | 7% |
| DID-S | TIS | NSt | branden | to burn | 1 | 7% |
| DID-S | TIS | NSt | chauffeur | driver | 1 | 7% |
| DID-S | TIS | NSt | cirkel | circle | 1 | 7% |
| DID-S | TIS | NSt | conflict | conflict | 1 | 7% |
| DID-S | NIS | NSt | consulaat | consulate | 1 | 7% |
| DID-S | NIS | NSt | depressie | depression | 1 | 7% |
| DID-S | TIS | NSt | domkop | idiot | 1 | 7% |
| DID-S | NIS | NSt | dreigen | to threaten | 1 | 7% |
| DID-S | TIS | NSt | dreigen | to threaten | 1 | 7% |
| DID-S | NIS | NSt | droefheid | sadness | 1 | 7% |
| DID-S | TIS | NSt | dwingen | to coerce or to force | 1 | 7% |
| DID-S | TIS | NSt | elleboog | elbow | 1 | 7% |
| DID-S | TIS | NSt | embryo | embryo | 1 | 7% |
| DID-S | NIS | NSt | ergeren | to annoy | 1 | 7% |
| DID-S | TIS | NSt | haken | hooks | 1 | 7% |
| DID-S | TIS | NSt | haten | to hate | 1 | 7% |
| DID-S | NIS | NSt | ijzer | iron | 1 | 7% |
| DID-S | NIS | NSt | ivoor | ivory | 1 | 7% |
| DID-S | TIS | NSt | kader | framework | 1 | 7% |
| DID-S | NIS | NSt | kerk | church | 1 | 7% |
| DID-S | TIS | NSt | kerk | church | 1 | 7% |
| DID-S | NIS | NSt | kou | cold | 1 | 7% |
| DID-S | TIS | NSt | kou | cold | 1 | 7% |
| DID-S | NIS | NSt | kwetsen | to hurt | 1 | 7% |
| DID-S | NIS | NSt | laden | to load | 1 | 7% |
| DID-S | NIS | NSt | liegen | to lie | 1 | 7% |
| DID-S | TIS | NSt | lijden | to suffer | 1 | 7% |
| DID-S | TIS | NSt | likken | to lick | 1 | 7% |
| DID-S | NIS | NSt | meester | master | 1 | 7% |
| DID-S | TIS | NSt | meester | master | 1 | 7% |
| DID-S | TIS | NSt | mes | knife | 1 | 7% |
| DID-S | TIS | NSt | metaal | metal | 1 | 7% |
| DID-S | TIS | NSt | misleiden | to deceive | 1 | 7% |
| DID-S | TIS | NSt | mismaakt | deformed | 1 | 7% |
| DID-S | TIS | NSt | misvormen | to deform | 1 | 7% |
| DID-S | TIS | NSt | noodkreet | cry for help | 1 | 7% |
| DID-S | TIS | NSt | octaaf | octave | 1 | 7% |
| DID-S | TIS | NSt | ogen | eyes | 1 | 7% |
| DID-S | TIS | NSt | ongeval | accident | 1 | 7% |
| DID-S | TIS | NSt | ongewenst | unwanted | 1 | 7% |
| DID-S | TIS | NSt | ontslag | resignation or dismissal | 1 | 7% |
| DID-S | NIS | NSt | onzeker | uncertain | 1 | 7% |
| DID-S | TIS | NSt | oom | uncle | 1 | 7% |
| DID-S | TIS | NSt | piekeren | to mull | 1 | 7% |
| DID-S | TIS | NSt | piemel | willy | 1 | 7% |
| DID-S | NIS | NSt | pijn | pain | 1 | 7% |
| DID-S | TIS | NSt | pijn | pain | 1 | 7% |
| DID-S | TIS | NSt | pilaar | pillar | 1 | 7% |
| DID-S | TIS | NSt | razernij | fury | 1 | 7% |
| DID-S | TIS | NSt | register | register | 1 | 7% |
| DID-S | TIS | NSt | ruzie | fight or quarrel | 1 | 7% |
| DID-S | NIS | NSt | schaden | to damage | 1 | 7% |
| DID-S | TIS | NSt | schaden | to damage | 1 | 7% |
| DID-S | NIS | NSt | schande | shame | 1 | 7% |
| DID-S | TIS | NSt | schande | shame | 1 | 7% |
| DID-S | TIS | NSt | schok | shock | 1 | 7% |
| DID-S | NIS | NSt | schoppen | to kick | 1 | 7% |
| DID-S | TIS | NSt | schroef | screw | 1 | 7% |
| DID-S | TIS | NSt | snauwen | to snarl | 1 | 7% |
| DID-S | TIS | NSt | snijden | to cut | 1 | 7% |
| DID-S | TIS | NSt | spoelen | to flush or to rinse | 1 | 7% |
| DID-S | TIS | NSt | steil | steep | 1 | 7% |
| DID-S | NIS | NSt | stellen | to set | 1 | 7% |
| DID-S | TIS | NSt | stiekem | secretly | 1 | 7% |
| DID-S | TIS | NSt | stikken | to suffocate | 1 | 7% |
| DID-S | TIS | NSt | stomerij | drycleaner | 1 | 7% |
| DID-S | TIS | NSt | tegel | tile | 1 | 7% |
| DID-S | TIS | NSt | traject | route | 1 | 7% |
| DID-S | NIS | NSt | trappen | to kick | 1 | 7% |
| DID-S | NIS | NSt | uitgescholden | scolded | 1 | 7% |
| DID-S | TIS | NSt | uitlachen | to laugh at | 1 | 7% |
| DID-S | TIS | NSt | vader | father | 1 | 7% |
| DID-S | TIS | NSt | vastbinden | to tie | 1 | 7% |
| DID-S | NIS | NSt | verdriet | sadness | 1 | 7% |
| DID-S | TIS | NSt | vergroten | enlarge | 1 | 7% |
| DID-S | TIS | NSt | verraad | betrayal | 1 | 7% |
| DID-S | TIS | NSt | vies | dirty | 1 | 7% |
| DID-S | TIS | NSt | vreemde | stranger | 1 | 7% |
| DID-S | TIS | NSt | walging | disgust | 1 | 7% |
| DID-S | NIS | NSt | woest | enraged | 1 | 7% |
| DID-S | TIS | NSt | woest | enraged | 1 | 7% |
| DID-S | TIS | NSt | wraak | revenge | 1 | 7% |
| DID-S | TIS | NSt | zakje | little bag | 1 | 7% |
| DID-S | NIS | NSt | zuigen | to suck | 1 | 7% |
| DID-S | TIS | NSn | aanslag | attack | 1 | 7% |
| DID-S | NIS | NSn | achterlaten | leave behind | 1 | 7% |
| DID-S | TIS | NSn | afblaffen | to bark at | 1 | 7% |
| DID-S | TIS | NSn | afgrijzen | horror | 1 | 7% |
| DID-S | TIS | NSn | afhakken | to chop off | 1 | 7% |
| DID-S | TIS | NSn | afkeer | aversion | 1 | 7% |
| DID-S | NIS | NSn | afscheid | goodbye | 1 | 7% |
| DID-S | TIS | NSn | afscheid | goodbye | 1 | 7% |
| DID-S | NIS | NSn | afschuw | revulsion | 1 | 7% |
| DID-S | TIS | NSn | afschuw | revulsion | 1 | 7% |
| DID-S | NIS | NSn | alleen | alone | 1 | 7% |
| DID-S | NIS | NSn | armoede | poverty | 1 | 7% |
| DID-S | TIS | NSn | baby | baby | 1 | 7% |
| DID-S | TIS | NSn | bad | bath | 1 | 7% |
| DID-S | TIS | NSn | bed | bed | 1 | 7% |
| DID-S | NIS | NSn | bedrog | deceit | 1 | 7% |
| DID-S | NIS | NSn | begraven | to bury | 1 | 7% |
| DID-S | TIS | NSn | beklemmen | to oppress | 1 | 7% |
| DID-S | NIS | NSn | belazerd | fooled | 1 | 7% |
| DID-S | TIS | NSn | belazerd | fooled | 1 | 7% |
| DID-S | NIS | NSn | benauwen | to agitate | 1 | 7% |
| DID-S | NIS | NSn | beroerte | stroke | 1 | 7% |
| DID-S | TIS | NSn | bordeel | brothel | 1 | 7% |
| DID-S | NIS | NSn | branden | to burn | 1 | 7% |
| DID-S | NIS | NSn | buurman | neighbor | 1 | 7% |
| DID-S | TIS | NSn | chanteren | to blackmail | 1 | 7% |
| DID-S | TIS | NSn | conflict | conflict | 1 | 7% |
| DID-S | TIS | NSn | crimineel | criminal | 1 | 7% |
| DID-S | TIS | NSn | diefstal | theft | 1 | 7% |
| DID-S | TIS | NSn | domkop | idiot | 1 | 7% |
| DID-S | NIS | NSn | doodgaan | to die | 1 | 7% |
| DID-S | TIS | NSn | doodgaan | to die | 1 | 7% |
| DID-S | TIS | NSn | doodslag | manslaughter | 1 | 7% |
| DID-S | NIS | NSn | droevig | sad | 1 | 7% |
| DID-S | NIS | NSn | dubbel | double | 1 | 7% |
| DID-S | TIS | NSn | dubbel | double | 1 | 7% |
| DID-S | TIS | NSn | elleboog | elbow | 1 | 7% |
| DID-S | NIS | NSn | etter | pus | 1 | 7% |
| DID-S | TIS | NSn | falen | to fail | 1 | 7% |
| DID-S | TIS | NSn | gezwel | tumor | 1 | 7% |
| DID-S | TIS | NSn | hagedis | lizard | 1 | 7% |
| DID-S | TIS | NSn | haken | hooks | 1 | 7% |
| DID-S | TIS | NSn | handen | hands | 1 | 7% |
| DID-S | TIS | NSn | hijgen | to pant | 1 | 7% |
| DID-S | NIS | NSn | hoer | whore | 1 | 7% |
| DID-S | TIS | NSn | hoer | whore | 1 | 7% |
| DID-S | NIS | NSn | hysterie | hysteria | 1 | 7% |
| DID-S | TIS | NSn | inbraak | burglary | 1 | 7% |
| DID-S | TIS | NSn | incest | incest | 1 | 7% |
| DID-S | NIS | NSn | instorten | to collapse | 1 | 7% |
| DID-S | TIS | NSn | kanker | cancer | 1 | 7% |
| DID-S | TIS | NSn | kelder | basement | 1 | 7% |
| DID-S | NIS | NSn | krenken | to hurt | 1 | 7% |
| DID-S | TIS | NSn | krenken | to hurt | 1 | 7% |
| DID-S | TIS | NSn | kwetsen | to hurt | 1 | 7% |
| DID-S | TIS | NSn | legpuzzel | jigsaw puzzle | 1 | 7% |
| DID-S | NIS | NSn | lepra | leprosy | 1 | 7% |
| DID-S | TIS | NSn | liegen | to lie | 1 | 7% |
| DID-S | NIS | NSn | man | man | 1 | 7% |
| DID-S | TIS | NSn | martelen | to torture | 1 | 7% |
| DID-S | NIS | NSn | masker | mask | 1 | 7% |
| DID-S | TIS | NSn | masker | mask | 1 | 7% |
| DID-S | TIS | NSn | meeloper | opportunist | 1 | 7% |
| DID-S | NIS | NSn | meester | master | 1 | 7% |
| DID-S | TIS | NSn | mes | knife | 1 | 7% |
| DID-S | TIS | NSn | middel | middle | 1 | 7% |
| DID-S | TIS | NSn | misdrijf | crime | 1 | 7% |
| DID-S | TIS | NSn | miskraam | miscarriage | 1 | 7% |
| DID-S | NIS | NSn | misleiden | to deceive | 1 | 7% |
| DID-S | TIS | NSn | misleiden | to deceive | 1 | 7% |
| DID-S | TIS | NSn | mislukt | failed | 1 | 7% |
| DID-S | TIS | NSn | mismaakt | deformed | 1 | 7% |
| DID-S | TIS | NSn | misvormen | to deform | 1 | 7% |
| DID-S | TIS | NSn | moedeloos | despondent | 1 | 7% |
| DID-S | NIS | NSn | moeder | mother | 1 | 7% |
| DID-S | NIS | NSn | mond | mouth | 1 | 7% |
| DID-S | NIS | NSn | nacht | night | 1 | 7% |
| DID-S | TIS | NSn | nacht | night | 1 | 7% |
| DID-S | TIS | NSn | octaaf | octave | 1 | 7% |
| DID-S | TIS | NSn | omkomen | to perish | 1 | 7% |
| DID-S | NIS | NSn | ongeval | accident | 1 | 7% |
| DID-S | TIS | NSn | onmacht | powerlessness | 1 | 7% |
| DID-S | NIS | NSn | ontslag | resignation or dismissal | 1 | 7% |
| DID-S | TIS | NSn | onzeker | uncertain | 1 | 7% |
| DID-S | NIS | NSn | oom | uncle | 1 | 7% |
| DID-S | TIS | NSn | oorlog | war | 1 | 7% |
| DID-S | NIS | NSn | oplichten | to scam | 1 | 7% |
| DID-S | TIS | NSn | oplichten | to scam | 1 | 7% |
| DID-S | TIS | NSn | orgie | orgy | 1 | 7% |
| DID-S | TIS | NSn | pedofiel | paedophile | 1 | 7% |
| DID-S | TIS | NSn | piekeren | to mull | 1 | 7% |
| DID-S | TIS | NSn | piemel | willy | 1 | 7% |
| DID-S | TIS | NSn | razernij | fury | 1 | 7% |
| DID-S | NIS | NSn | regel | rule | 1 | 7% |
| DID-S | TIS | NSn | roofmoord | robbery with murder | 1 | 7% |
| DID-S | TIS | NSn | schaden | to damage | 1 | 7% |
| DID-S | NIS | NSn | schoft | bastard | 1 | 7% |
| DID-S | NIS | NSn | schok | shock | 1 | 7% |
| DID-S | NIS | NSn | seks | sex | 1 | 7% |
| DID-S | NIS | NSn | slijmen | suck up to | 1 | 7% |
| DID-S | TIS | NSn | snijden | to cut | 1 | 7% |
| DID-S | NIS | NSn | tegenslag | setback | 1 | 7% |
| DID-S | NIS | NSn | trappen | to kick | 1 | 7% |
| DID-S | TIS | NSn | trappen | to kick | 1 | 7% |
| DID-S | TIS | NSn | trauma | trauma | 1 | 7% |
| DID-S | TIS | NSn | treiteren | to harass | 1 | 7% |
| DID-S | TIS | NSn | uitgescholden | scolded | 1 | 7% |
| DID-S | TIS | NSn | uitkleden | to undress | 1 | 7% |
| DID-S | NIS | NSn | uitlachen | to laugh at | 1 | 7% |
| DID-S | NIS | NSn | vastbinden | to tie | 1 | 7% |
| DID-S | TIS | NSn | verbranden | to burn | 1 | 7% |
| DID-S | TIS | NSn | verdrinken | to drown | 1 | 7% |
| DID-S | NIS | NSn | vies | dirty | 1 | 7% |
| DID-S | NIS | NSn | wanhopen | to despair | 1 | 7% |
| DID-S | TIS | NSn | woest | enraged | 1 | 7% |
| DID-S | NIS | NSn | zeer | ache | 1 | 7% |
| DID-S | TIS | NSn | zondebok | scapegoat | 1 | 7% |
| DID-S | TIS | NSn | zuigen | to suck | 1 | 7% |
| CTRL^^n3^ | NIS | NSt | incest | incest | 14 | 93% |
| CTRL | NIS | NSt | moord | murder | 14 | 93% |
| CTRL | NIS | NSt | wurgen | to strangle | 14 | 93% |
| CTRL | TIS | St | bang | afraid | 13 | 87% |
| CTRL | NIS | NSt | bloedbad | bloodbath | 13 | 87% |
| CTRL | NIS | NSt | doodslag | manslaughter | 13 | 87% |
| CTRL | NIS | NSt | folteren | to torture | 13 | 87% |
| CTRL | NIS | NSt | martelen | to torture | 13 | 87% |
| CTRL | NIS | NSt | misdrijf | crime | 13 | 87% |
| CTRL | NIS | NSt | oorlog | war | 13 | 87% |
| CTRL | NIS | NSt | stikken | to suffocate | 13 | 87% |
| CTRL | TIS | St | onrecht | injustice | 12 | 80% |
| CTRL | TIS | NSt | bloedbad | bloodbath | 12 | 80% |
| CTRL | NIS | NSt | crimineel | criminal | 12 | 80% |
| CTRL | NIS | NSt | doden | to kill | 12 | 80% |
| CTRL | NIS | NSt | verdrinken | to drown | 12 | 80% |
| CTRL | NIS | NSt | verkrachting | rape | 12 | 80% |
| CTRL | NIS | NSt | wreedheid | cruelty | 12 | 80% |
| CTRL | TIS | St | lijden | to suffer | 11 | 73% |
| CTRL | NIS | St | onzeker | uncertain | 11 | 73% |
| CTRL | NIS | NSt | doodsteek | deathblow | 11 | 73% |
| CTRL | TIS | NSt | oorlog | war | 11 | 73% |
| CTRL | NIS | NSt | roofmoord | robbery with murder | 11 | 73% |
| CTRL | TIS | NSt | roofmoord | robbery with murder | 11 | 73% |
| CTRL | TIS | NSn | regenton | rain barrel | 11 | 73% |
| CTRL | TIS | NSn | stukadoor | plasterer | 11 | 73% |
| CTRL | TIS | St | angst | fear | 10 | 67% |
| CTRL | TIS | NSt | doodslag | manslaughter | 10 | 67% |
| CTRL | NIS | NSt | geweld | violence | 10 | 67% |
| CTRL | NIS | NSt | gijzeling | kidnapping | 10 | 67% |
| CTRL | NIS | NSt | pedofiel | paedophile | 10 | 67% |
| CTRL | NIS | NSt | sadist | sadist | 10 | 67% |
| CTRL | TIS | NSt | steekwond | stab wound | 10 | 67% |
| CTRL | TIS | NSn | consulaat | consulate | 10 | 67% |
| CTRL | NIS | NSn | telegram | telegram | 10 | 67% |
| CTRL | NIS | St | ergeren | to annoy | 9 | 60% |
| CTRL | TIS | St | kwetsen | to hurt | 9 | 60% |
| CTRL | TIS | St | ongewenst | unwanted | 9 | 60% |
| CTRL | TIS | St | onmacht | powerlessness | 9 | 60% |
| CTRL | NIS | St | piekeren | to mull | 9 | 60% |
| CTRL | TIS | St | pijn | pain | 9 | 60% |
| CTRL | NIS | St | verdriet | sadness | 9 | 60% |
| CTRL | TIS | St | verdriet | sadness | 9 | 60% |
| CTRL | NIS | NSt | beroerte | stroke | 9 | 60% |
| CTRL | TIS | NSt | martelen | to torture | 9 | 60% |
| CTRL | NIS | NSt | messteek | knife stab | 9 | 60% |
| CTRL | TIS | NSt | moord | murder | 9 | 60% |
| CTRL | NIS | NSt | omkomen | to perish | 9 | 60% |
| CTRL | TIS | NSt | oplichten | to scam | 9 | 60% |
| CTRL | NIS | NSn | dakgoot | gutter | 9 | 60% |
| CTRL | NIS | NSn | haak | hook | 9 | 60% |
| CTRL | TIS | NSn | kenteken | license plate | 9 | 60% |
| CTRL | NIS | NSn | leuning | railing | 9 | 60% |
| CTRL | TIS | NSn | potlood | pencil | 9 | 60% |
| CTRL | TIS | NSn | stomerij | drycleaner | 9 | 60% |
| CTRL | NIS | NSn | stukadoor | plasterer | 9 | 60% |
| CTRL | TIS | NSn | tegel | tile | 9 | 60% |
| CTRL | NIS | NSn | tijdperk | era | 9 | 60% |
| CTRL | NIS | NSn | uitgever | publisher | 9 | 60% |
| CTRL | TIS | St | agressie | aggression | 8 | 53% |
| CTRL | TIS | St | benauwen | to agitate | 8 | 53% |
| CTRL | TIS | St | dwingen | to coerce or to force | 8 | 53% |
| CTRL | TIS | St | ellende | misery | 8 | 53% |
| CTRL | NIS | St | falen | to fail | 8 | 53% |
| CTRL | TIS | St | geweld | violence | 8 | 53% |
| CTRL | TIS | St | instorten | to collapse | 8 | 53% |
| CTRL | NIS | St | onmacht | powerlessness | 8 | 53% |
| CTRL | TIS | St | onzeker | uncertain | 8 | 53% |
| CTRL | TIS | St | ruzie | fight or quarrel | 8 | 53% |
| CTRL | NIS | St | tegenslag | setback | 8 | 53% |
| CTRL | TIS | St | trauma | trauma | 8 | 53% |
| CTRL | TIS | St | verkrachting | rape | 8 | 53% |
| CTRL | NIS | NSt | aanslag | attack | 8 | 53% |
| CTRL | NIS | NSt | atoombom | nuclear bomb | 8 | 53% |
| CTRL | TIS | NSt | atoombom | nuclear bomb | 8 | 53% |
| CTRL | TIS | NSt | beroerte | stroke | 8 | 53% |
| CTRL | TIS | NSt | doodsteek | deathblow | 8 | 53% |
| CTRL | TIS | NSt | haten | to hate | 8 | 53% |
| CTRL | NIS | NSt | misdaad | crime | 8 | 53% |
| CTRL | NIS | NSt | ontrouw | unfaithful | 8 | 53% |
| CTRL | NIS | NSt | slet | slut | 8 | 53% |
| CTRL | NIS | NSt | treiteren | to harass | 8 | 53% |
| CTRL | TIS | NSt | wurgen | to strangle | 8 | 53% |
| CTRL | NIS | NSn | bestek | cutlery | 8 | 53% |
| CTRL | NIS | NSn | flacon | bottle or vial | 8 | 53% |
| CTRL | TIS | NSn | grondstof | natural resource | 8 | 53% |
| CTRL | TIS | NSn | haak | hook | 8 | 53% |
| CTRL | NIS | NSn | ijzer | iron | 8 | 53% |
| CTRL | TIS | NSn | ijzer | iron | 8 | 53% |
| CTRL | TIS | NSn | leuning | railing | 8 | 53% |
| CTRL | NIS | NSn | metselaar | bricklayer | 8 | 53% |
| CTRL | TIS | NSn | metselaar | bricklayer | 8 | 53% |
| CTRL | NIS | NSn | plank | shelf | 8 | 53% |
| CTRL | NIS | NSn | register | register | 8 | 53% |
| CTRL | NIS | NSn | schuren | to polish | 8 | 53% |
| CTRL | TIS | NSn | stellen | to set | 8 | 53% |
| CTRL | TIS | NSn | telegram | telegram | 8 | 53% |
| CTRL | NIS | NSn | teller | counter | 8 | 53% |
| CTRL | NIS | NSn | uitkleden | to undress | 8 | 53% |
| CTRL | NIS | NSn | versie | version | 8 | 53% |
| CTRL | NIS | NSn | vierkant | square | 8 | 53% |
| CTRL | NIS | NSn | zakje | little bag | 8 | 53% |
| CTRL | TIS | NSn | zakje | little bag | 8 | 53% |
| CTRL | NIS | NSn | zandloper | hourglass | 8 | 53% |
| CTRL | TIS | NSn | zegel | seal | 8 | 53% |
| CTRL | TIS | St | afkraken | to decry | 7 | 47% |
| CTRL | TIS | St | bedreigen | to threaten | 7 | 47% |
| CTRL | TIS | St | beklemmen | to oppress | 7 | 47% |
| CTRL | TIS | St | dreigen | to threaten | 7 | 47% |
| CTRL | TIS | St | droefheid | sadness | 7 | 47% |
| CTRL | TIS | St | dwang | coercion or force | 7 | 47% |
| CTRL | NIS | St | ergernis | annoyance | 7 | 47% |
| CTRL | NIS | St | gezwel | tumor | 7 | 47% |
| CTRL | NIS | St | kou | cold | 7 | 47% |
| CTRL | TIS | St | misdrijf | crime | 7 | 47% |
| CTRL | TIS | St | schaden | to damage | 7 | 47% |
| CTRL | TIS | St | verraad | betrayal | 7 | 47% |
| CTRL | TIS | St | zeer | ache | 7 | 47% |
| CTRL | NIS | St | zeuren | to nag | 7 | 47% |
| CTRL | TIS | NSt | aanslag | attack | 7 | 47% |
| CTRL | NIS | NSt | afgrijzen | horror | 7 | 47% |
| CTRL | NIS | NSt | bedrieger | deceiver | 7 | 47% |
| CTRL | TIS | NSt | crimineel | criminal | 7 | 47% |
| CTRL | TIS | NSt | diefstal | theft | 7 | 47% |
| CTRL | TIS | NSt | doden | to kill | 7 | 47% |
| CTRL | NIS | NSt | ellende | misery | 7 | 47% |
| CTRL | TIS | NSt | folteren | to torture | 7 | 47% |
| CTRL | TIS | NSt | gijzeling | kidnapping | 7 | 47% |
| CTRL | NIS | NSt | haten | to hate | 7 | 47% |
| CTRL | TIS | NSt | kanker | cancer | 7 | 47% |
| CTRL | NIS | NSt | lepra | leprosy | 7 | 47% |
| CTRL | TIS | NSt | lepra | leprosy | 7 | 47% |
| CTRL | TIS | NSt | messteek | knife stab | 7 | 47% |
| CTRL | TIS | NSt | slet | slut | 7 | 47% |
| CTRL | NIS | NSt | tiran | tyrant | 7 | 47% |
| CTRL | NIS | NSt | verraad | betrayal | 7 | 47% |
| CTRL | TIS | NSt | verzuipen | to drown | 7 | 47% |
| CTRL | TIS | NSt | wraak | revenge | 7 | 47% |
| CTRL | TIS | NSn | bestek | cutlery | 7 | 47% |
| CTRL | NIS | NSn | consulaat | consulate | 7 | 47% |
| CTRL | NIS | NSn | element | element | 7 | 47% |
| CTRL | NIS | NSn | grondstof | natural resource | 7 | 47% |
| CTRL | TIS | NSn | ivoor | ivory | 7 | 47% |
| CTRL | NIS | NSn | omroep | broadcasting | 7 | 47% |
| CTRL | TIS | NSn | omroep | broadcasting | 7 | 47% |
| CTRL | TIS | NSn | postzegel | stamp | 7 | 47% |
| CTRL | TIS | NSn | roeren | to stir | 7 | 47% |
| CTRL | NIS | NSn | schroef | screw | 7 | 47% |
| CTRL | NIS | NSn | stomerij | drycleaner | 7 | 47% |
| CTRL | NIS | NSn | tegel | tile | 7 | 47% |
| CTRL | NIS | NSn | touw | rope | 7 | 47% |
| CTRL | NIS | NSn | traject | route | 7 | 47% |
| CTRL | TIS | NSn | versie | version | 7 | 47% |
| CTRL | TIS | St | afscheid | goodbye | 6 | 40% |
| CTRL | NIS | St | afwijzing | rejection | 6 | 40% |
| CTRL | TIS | St | belazerd | fooled | 6 | 40% |
| CTRL | NIS | St | kanker | cancer | 6 | 40% |
| CTRL | TIS | St | omkomen | to perish | 6 | 40% |
| CTRL | TIS | St | piekeren | to mull | 6 | 40% |
| CTRL | TIS | St | schuldig | guilty | 6 | 40% |
| CTRL | TIS | St | wanhopen | to despair | 6 | 40% |
| CTRL | NIS | NSt | chanteren | to blackmail | 6 | 40% |
| CTRL | TIS | NSt | chanteren | to blackmail | 6 | 40% |
| CTRL | TIS | NSt | hysterie | hysteria | 6 | 40% |
| CTRL | TIS | NSt | incest | incest | 6 | 40% |
| CTRL | TIS | NSt | kogel | bullet | 6 | 40% |
| CTRL | TIS | NSt | kreng | bitch | 6 | 40% |
| CTRL | NIS | NSt | kuthoer | pussy whore | 6 | 40% |
| CTRL | TIS | NSt | ontrouw | unfaithful | 6 | 40% |
| CTRL | TIS | NSt | pedofiel | paedophile | 6 | 40% |
| CTRL | TIS | NSt | sadist | sadist | 6 | 40% |
| CTRL | TIS | NSt | verdrinken | to drown | 6 | 40% |
| CTRL | NIS | NSt | verzuipen | to drown | 6 | 40% |
| CTRL | NIS | NSn | cirkel | circle | 6 | 40% |
| CTRL | TIS | NSn | elleboog | elbow | 6 | 40% |
| CTRL | NIS | NSn | etiket | label | 6 | 40% |
| CTRL | NIS | NSn | firma | firm | 6 | 40% |
| CTRL | TIS | NSn | firma | firm | 6 | 40% |
| CTRL | TIS | NSn | flacon | bottle or vial | 6 | 40% |
| CTRL | NIS | NSn | gaatje | little hole | 6 | 40% |
| CTRL | TIS | NSn | geit | goat | 6 | 40% |
| CTRL | TIS | NSn | hertogin | duchess | 6 | 40% |
| CTRL | TIS | NSn | kast | closet | 6 | 40% |
| CTRL | NIS | NSn | kenteken | license plate | 6 | 40% |
| CTRL | TIS | NSn | paragraaf | paragraph | 6 | 40% |
| CTRL | NIS | NSn | plafond | ceiling | 6 | 40% |
| CTRL | NIS | NSn | rad | wheel | 6 | 40% |
| CTRL | TIS | NSn | rad | wheel | 6 | 40% |
| CTRL | TIS | NSn | schroef | screw | 6 | 40% |
| CTRL | NIS | NSn | stellen | to set | 6 | 40% |
| CTRL | TIS | NSn | teller | counter | 6 | 40% |
| CTRL | NIS | NSn | theelepel | teaspoon | 6 | 40% |
| CTRL | TIS | NSn | tijdperk | era | 6 | 40% |
| CTRL | TIS | NSn | vierkant | square | 6 | 40% |
| CTRL | TIS | NSn | zandloper | hourglass | 6 | 40% |
| CTRL | NIS | NSn | zegel | seal | 6 | 40% |
| CTRL | NIS | NSn | zetel | seat | 6 | 40% |
| CTRL | TIS | St | achterlaten | leave behind | 5 | 33% |
| CTRL | NIS | St | afscheid | goodbye | 5 | 33% |
| CTRL | TIS | St | afwijzing | rejection | 5 | 33% |
| CTRL | TIS | St | alleen | alone | 5 | 33% |
| CTRL | NIS | St | angst | fear | 5 | 33% |
| CTRL | TIS | St | bedrieger | deceiver | 5 | 33% |
| CTRL | TIS | St | boos | angry | 5 | 33% |
| CTRL | NIS | St | conflict | conflict | 5 | 33% |
| CTRL | TIS | St | depressie | depression | 5 | 33% |
| CTRL | NIS | St | doodgaan | to die | 5 | 33% |
| CTRL | NIS | St | droefheid | sadness | 5 | 33% |
| CTRL | NIS | St | droevig | sad | 5 | 33% |
| CTRL | TIS | St | kanker | cancer | 5 | 33% |
| CTRL | NIS | St | lawaai | noise | 5 | 33% |
| CTRL | TIS | St | lawaai | noise | 5 | 33% |
| CTRL | TIS | St | moedeloos | despondent | 5 | 33% |
| CTRL | TIS | St | pedofiel | paedophile | 5 | 33% |
| CTRL | TIS | St | schok | shock | 5 | 33% |
| CTRL | TIS | St | schoppen | to kick | 5 | 33% |
| CTRL | TIS | St | tegenslag | setback | 5 | 33% |
| CTRL | TIS | St | wanhoop | despair | 5 | 33% |
| CTRL | TIS | NSt | afblaffen | to bark at | 5 | 33% |
| CTRL | TIS | NSt | afgunst | envy | 5 | 33% |
| CTRL | TIS | NSt | bedrieger | deceiver | 5 | 33% |
| CTRL | TIS | NSt | inbraak | burglary | 5 | 33% |
| CTRL | TIS | NSt | lafaard | coward | 5 | 33% |
| CTRL | TIS | NSt | liegen | to lie | 5 | 33% |
| CTRL | NIS | NSt | miskraam | miscarriage | 5 | 33% |
| CTRL | TIS | NSt | slaaf | slave | 5 | 33% |
| CTRL | NIS | NSt | steekwond | stab wound | 5 | 33% |
| CTRL | TIS | NSt | tiran | tyrant | 5 | 33% |
| CTRL | NIS | NSt | trauma | trauma | 5 | 33% |
| CTRL | NIS | NSt | verlammen | to paralyze | 5 | 33% |
| CTRL | TIS | NSn | bordeel | brothel | 5 | 33% |
| CTRL | TIS | NSn | chauffeur | driver | 5 | 33% |
| CTRL | NIS | NSn | citaat | quote | 5 | 33% |
| CTRL | TIS | NSn | dakgoot | gutter | 5 | 33% |
| CTRL | NIS | NSn | dwerg | dwarf | 5 | 33% |
| CTRL | NIS | NSn | embryo | embryo | 5 | 33% |
| CTRL | NIS | NSn | gebouw | building | 5 | 33% |
| CTRL | NIS | NSn | geit | goat | 5 | 33% |
| CTRL | TIS | NSn | handen | hands | 5 | 33% |
| CTRL | NIS | NSn | kast | closet | 5 | 33% |
| CTRL | NIS | NSn | kelder | basement | 5 | 33% |
| CTRL | NIS | NSn | kozijn | window frame | 5 | 33% |
| CTRL | NIS | NSn | laden | to load | 5 | 33% |
| CTRL | TIS | NSn | meester | master | 5 | 33% |
| CTRL | NIS | NSn | metaal | metal | 5 | 33% |
| CTRL | NIS | NSn | middel | middle | 5 | 33% |
| CTRL | TIS | NSn | plafond | ceiling | 5 | 33% |
| CTRL | TIS | NSn | plank | shelf | 5 | 33% |
| CTRL | NIS | NSn | potlood | pencil | 5 | 33% |
| CTRL | TIS | NSn | register | register | 5 | 33% |
| CTRL | NIS | NSn | roeren | to stir | 5 | 33% |
| CTRL | TIS | NSn | schuren | to polish | 5 | 33% |
| CTRL | NIS | NSn | spoelen | to flush or to rinse | 5 | 33% |
| CTRL | NIS | NSn | tapijt | tapestry | 5 | 33% |
| CTRL | TIS | NSn | tapijt | tapestry | 5 | 33% |
| CTRL | TIS | NSn | theelepel | teaspoon | 5 | 33% |
| CTRL | TIS | NSn | touw | rope | 5 | 33% |
| CTRL | TIS | NSn | traject | route | 5 | 33% |
| CTRL | NIS | NSn | trede | step | 5 | 33% |
| CTRL | TIS | NSn | trede | step | 5 | 33% |
| CTRL | NIS | NSn | treden | to step | 5 | 33% |
| CTRL | NIS | NSn | trottoir | pavement | 5 | 33% |
| CTRL | TIS | NSn | uitgever | publisher | 5 | 33% |
| CTRL | TIS | NSn | verleidster | temptress | 5 | 33% |
| CTRL | TIS | NSn | zetel | seat | 5 | 33% |
| CTRL | TIS | St | afsnauwen | to snap at | 4 | 27% |
| CTRL | NIS | St | bang | afraid | 4 | 27% |
| CTRL | TIS | St | begraven | to bury | 4 | 27% |
| CTRL | NIS | St | boos | angry | 4 | 27% |
| CTRL | TIS | St | broer | brother | 4 | 27% |
| CTRL | TIS | St | conflict | conflict | 4 | 27% |
| CTRL | NIS | St | depressie | depression | 4 | 27% |
| CTRL | TIS | St | doden | to kill | 4 | 27% |
| CTRL | TIS | St | droevig | sad | 4 | 27% |
| CTRL | TIS | St | falen | to fail | 4 | 27% |
| CTRL | TIS | St | gezwel | tumor | 4 | 27% |
| CTRL | NIS | St | krabben | to scratch | 4 | 27% |
| CTRL | TIS | St | krenken | to hurt | 4 | 27% |
| CTRL | NIS | St | kwetsen | to hurt | 4 | 27% |
| CTRL | TIS | St | leugen | lie | 4 | 27% |
| CTRL | TIS | St | miskraam | miscarriage | 4 | 27% |
| CTRL | TIS | St | misleiden | to deceive | 4 | 27% |
| CTRL | TIS | St | noodkreet | cry for help | 4 | 27% |
| CTRL | TIS | St | ongeluk | accident | 4 | 27% |
| CTRL | TIS | St | ontrouw | unfaithful | 4 | 27% |
| CTRL | NIS | St | pijn | pain | 4 | 27% |
| CTRL | TIS | St | sadist | sadist | 4 | 27% |
| CTRL | TIS | St | snauwen | to snarl | 4 | 27% |
| CTRL | TIS | St | stiekem | secretly | 4 | 27% |
| CTRL | TIS | St | stikken | to suffocate | 4 | 27% |
| CTRL | TIS | St | tiran | tyrant | 4 | 27% |
| CTRL | NIS | St | trauma | trauma | 4 | 27% |
| CTRL | TIS | St | verlammen | to paralyze | 4 | 27% |
| CTRL | TIS | St | vernielen | to vandalize | 4 | 27% |
| CTRL | TIS | St | verzuipen | to drown | 4 | 27% |
| CTRL | NIS | St | wanhopen | to despair | 4 | 27% |
| CTRL | TIS | St | wreedheid | cruelty | 4 | 27% |
| CTRL | NIS | NSt | agressie | aggression | 4 | 27% |
| CTRL | NIS | NSt | armoede | poverty | 4 | 27% |
| CTRL | TIS | NSt | armoede | poverty | 4 | 27% |
| CTRL | NIS | NSt | bedreigen | to threaten | 4 | 27% |
| CTRL | NIS | NSt | doodgaan | to die | 4 | 27% |
| CTRL | NIS | NSt | dwang | coercion or force | 4 | 27% |
| CTRL | TIS | NSt | getreiter | harassment | 4 | 27% |
| CTRL | NIS | NSt | gezwel | tumor | 4 | 27% |
| CTRL | TIS | NSt | gezwel | tumor | 4 | 27% |
| CTRL | TIS | NSt | hoer | whore | 4 | 27% |
| CTRL | NIS | NSt | kanker | cancer | 4 | 27% |
| CTRL | NIS | NSt | kogel | bullet | 4 | 27% |
| CTRL | TIS | NSt | kotsen | to vomit | 4 | 27% |
| CTRL | NIS | NSt | liegen | to lie | 4 | 27% |
| CTRL | NIS | NSt | lijden | to suffer | 4 | 27% |
| CTRL | TIS | NSt | miskraam | miscarriage | 4 | 27% |
| CTRL | TIS | NSt | mismaakt | deformed | 4 | 27% |
| CTRL | TIS | NSt | omkomen | to perish | 4 | 27% |
| CTRL | TIS | NSt | ontslag | resignation or dismissal | 4 | 27% |
| CTRL | NIS | NSt | oplichten | to scam | 4 | 27% |
| CTRL | NIS | NSt | opsluiten | to lock up | 4 | 27% |
| CTRL | TIS | NSt | orgie | orgy | 4 | 27% |
| CTRL | TIS | NSt | razernij | fury | 4 | 27% |
| CTRL | NIS | NSt | snauwen | to snarl | 4 | 27% |
| CTRL | TIS | NSt | stinken | to stink | 4 | 27% |
| CTRL | TIS | NSt | treiteren | to harass | 4 | 27% |
| CTRL | TIS | NSt | uitlachen | to laugh at | 4 | 27% |
| CTRL | TIS | NSt | vastbinden | to tie | 4 | 27% |
| CTRL | NIS | NSt | wanhoop | despair | 4 | 27% |
| CTRL | NIS | NSn | absorptie | absorption | 4 | 27% |
| CTRL | TIS | NSn | absorptie | absorption | 4 | 27% |
| CTRL | NIS | NSn | blikje | can | 4 | 27% |
| CTRL | TIS | NSn | citaat | quote | 4 | 27% |
| CTRL | NIS | NSn | deurknop | door knob | 4 | 27% |
| CTRL | NIS | NSn | doorslikken | to swallow | 4 | 27% |
| CTRL | NIS | NSn | dubbel | double | 4 | 27% |
| CTRL | NIS | NSn | elleboog | elbow | 4 | 27% |
| CTRL | NIS | NSn | haken | hooks | 4 | 27% |
| CTRL | TIS | NSn | haken | hooks | 4 | 27% |
| CTRL | NIS | NSn | hertogin | duchess | 4 | 27% |
| CTRL | NIS | NSn | ivoor | ivory | 4 | 27% |
| CTRL | NIS | NSn | kader | framework | 4 | 27% |
| CTRL | TIS | NSn | laden | to load | 4 | 27% |
| CTRL | TIS | NSn | likken | to lick | 4 | 27% |
| CTRL | NIS | NSn | meester | master | 4 | 27% |
| CTRL | TIS | NSn | middel | middle | 4 | 27% |
| CTRL | NIS | NSn | octaaf | octave | 4 | 27% |
| CTRL | TIS | NSn | octaaf | octave | 4 | 27% |
| CTRL | TIS | NSn | orgie | orgy | 4 | 27% |
| CTRL | NIS | NSn | paragraaf | paragraph | 4 | 27% |
| CTRL | TIS | NSn | pasen | Easter | 4 | 27% |
| CTRL | TIS | NSn | pilaar | pillar | 4 | 27% |
| CTRL | NIS | NSn | regenton | rain barrel | 4 | 27% |
| CTRL | NIS | NSn | takken | branches | 4 | 27% |
| CTRL | TIS | NSn | takken | branches | 4 | 27% |
| CTRL | TIS | NSn | trottoir | pavement | 4 | 27% |
| CTRL | TIS | NSn | uitkleden | to undress | 4 | 27% |
| CTRL | TIS | NSn | verbranden | to burn | 4 | 27% |
| CTRL | NIS | NSn | vreemde | stranger | 4 | 27% |
| CTRL | TIS | St | afblaffen | to bark at | 3 | 20% |
| CTRL | NIS | St | afkraken | to decry | 3 | 20% |
| CTRL | NIS | St | alleen | alone | 3 | 20% |
| CTRL | TIS | St | bedrog | deceit | 3 | 20% |
| CTRL | NIS | St | benauwen | to agitate | 3 | 20% |
| CTRL | NIS | St | beroerte | stroke | 3 | 20% |
| CTRL | NIS | St | besmetten | to contaminate | 3 | 20% |
| CTRL | TIS | St | doodgaan | to die | 3 | 20% |
| CTRL | TIS | St | dubbel | double | 3 | 20% |
| CTRL | NIS | St | dwingen | to coerce or to force | 3 | 20% |
| CTRL | TIS | St | gijzeling | kidnapping | 3 | 20% |
| CTRL | NIS | St | inbraak | burglary | 3 | 20% |
| CTRL | TIS | St | knijpen | to pinch | 3 | 20% |
| CTRL | NIS | St | kotsen | to vomit | 3 | 20% |
| CTRL | NIS | St | krenken | to hurt | 3 | 20% |
| CTRL | TIS | St | liegen | to lie | 3 | 20% |
| CTRL | NIS | St | lijden | to suffer | 3 | 20% |
| CTRL | TIS | St | mes | knife | 3 | 20% |
| CTRL | TIS | St | nacht | night | 3 | 20% |
| CTRL | TIS | St | ongeval | accident | 3 | 20% |
| CTRL | NIS | St | onrecht | injustice | 3 | 20% |
| CTRL | TIS | St | razernij | fury | 3 | 20% |
| CTRL | NIS | St | ruzie | fight or quarrel | 3 | 20% |
| CTRL | TIS | St | schoft | bastard | 3 | 20% |
| CTRL | NIS | St | schok | shock | 3 | 20% |
| CTRL | NIS | St | schuldig | guilty | 3 | 20% |
| CTRL | NIS | St | snauwen | to snarl | 3 | 20% |
| CTRL | NIS | St | sterven | to die | 3 | 20% |
| CTRL | TIS | St | uitgescholden | scolded | 3 | 20% |
| CTRL | TIS | St | uitlachen | to laugh at | 3 | 20% |
| CTRL | TIS | St | vader | father | 3 | 20% |
| CTRL | NIS | NSt | afkraken | to decry | 3 | 20% |
| CTRL | NIS | NSt | afsnauwen | to snap at | 3 | 20% |
| CTRL | TIS | NSt | agressie | aggression | 3 | 20% |
| CTRL | NIS | NSt | belazerd | fooled | 3 | 20% |
| CTRL | TIS | NSt | bordeel | brothel | 3 | 20% |
| CTRL | TIS | NSt | domkop | idiot | 3 | 20% |
| CTRL | NIS | NSt | dwingen | to coerce or to force | 3 | 20% |
| CTRL | TIS | NSt | dwingen | to coerce or to force | 3 | 20% |
| CTRL | NIS | NSt | hoer | whore | 3 | 20% |
| CTRL | NIS | NSt | hysterie | hysteria | 3 | 20% |
| CTRL | NIS | NSt | kotsen | to vomit | 3 | 20% |
| CTRL | NIS | NSt | krenken | to hurt | 3 | 20% |
| CTRL | TIS | NSt | kuthoer | pussy whore | 3 | 20% |
| CTRL | TIS | NSt | misdaad | crime | 3 | 20% |
| CTRL | TIS | NSt | misdrijf | crime | 3 | 20% |
| CTRL | TIS | NSt | misleiden | to deceive | 3 | 20% |
| CTRL | NIS | NSt | mismaakt | deformed | 3 | 20% |
| CTRL | NIS | NSt | ongeluk | accident | 3 | 20% |
| CTRL | TIS | NSt | ongeval | accident | 3 | 20% |
| CTRL | TIS | NSt | opsluiten | to lock up | 3 | 20% |
| CTRL | NIS | NSt | pijn | pain | 3 | 20% |
| CTRL | NIS | NSt | ruzie | fight or quarrel | 3 | 20% |
| CTRL | NIS | NSt | schoft | bastard | 3 | 20% |
| CTRL | NIS | NSt | schoppen | to kick | 3 | 20% |
| CTRL | NIS | NSt | slaaf | slave | 3 | 20% |
| CTRL | TIS | NSt | slecht | bad | 3 | 20% |
| CTRL | TIS | NSt | slijmen | suck up to | 3 | 20% |
| CTRL | TIS | NSt | snijden | to cut | 3 | 20% |
| CTRL | TIS | NSt | stank | stench | 3 | 20% |
| CTRL | NIS | NSt | sterven | to die | 3 | 20% |
| CTRL | TIS | NSt | stikken | to suffocate | 3 | 20% |
| CTRL | TIS | NSt | verdord | withered | 3 | 20% |
| CTRL | TIS | NSt | verkrachting | rape | 3 | 20% |
| CTRL | TIS | NSt | verlammen | to paralyze | 3 | 20% |
| CTRL | TIS | NSt | vies | dirty | 3 | 20% |
| CTRL | NIS | NSt | walging | disgust | 3 | 20% |
| CTRL | TIS | NSt | walging | disgust | 3 | 20% |
| CTRL | TIS | NSt | wreedheid | cruelty | 3 | 20% |
| CTRL | TIS | NSt | zondebok | scapegoat | 3 | 20% |
| CTRL | NIS | NSn | aanmaken | to prepare | 3 | 20% |
| CTRL | TIS | NSn | aanmaken | to prepare | 3 | 20% |
| CTRL | TIS | NSn | afhakken | to chop off | 3 | 20% |
| CTRL | TIS | NSn | atoombom | nuclear bomb | 3 | 20% |
| CTRL | TIS | NSn | besmetten | to contaminate | 3 | 20% |
| CTRL | NIS | NSn | bladzijde | page | 3 | 20% |
| CTRL | TIS | NSn | bladzijde | page | 3 | 20% |
| CTRL | TIS | NSn | blikje | can | 3 | 20% |
| CTRL | NIS | NSn | blinddoek | blindfold | 3 | 20% |
| CTRL | TIS | NSn | blinddoek | blindfold | 3 | 20% |
| CTRL | NIS | NSn | bloot | naked | 3 | 20% |
| CTRL | NIS | NSn | branden | to burn | 3 | 20% |
| CTRL | TIS | NSn | cirkel | circle | 3 | 20% |
| CTRL | TIS | NSn | deurknop | door knob | 3 | 20% |
| CTRL | NIS | NSn | dozijn | dozen | 3 | 20% |
| CTRL | TIS | NSn | dozijn | dozen | 3 | 20% |
| CTRL | TIS | NSn | dwerg | dwarf | 3 | 20% |
| CTRL | TIS | NSn | element | element | 3 | 20% |
| CTRL | TIS | NSn | etter | pus | 3 | 20% |
| CTRL | TIS | NSn | gaatje | little hole | 3 | 20% |
| CTRL | TIS | NSn | kelder | basement | 3 | 20% |
| CTRL | TIS | NSn | knijpen | to pinch | 3 | 20% |
| CTRL | NIS | NSn | kreunen | to moan | 3 | 20% |
| CTRL | NIS | NSn | legpuzzel | jigsaw puzzle | 3 | 20% |
| CTRL | NIS | NSn | likken | to lick | 3 | 20% |
| CTRL | TIS | NSn | magazijn | warehouse | 3 | 20% |
| CTRL | NIS | NSn | mes | knife | 3 | 20% |
| CTRL | TIS | NSn | metaal | metal | 3 | 20% |
| CTRL | NIS | NSn | nacht | night | 3 | 20% |
| CTRL | NIS | NSn | piemel | willy | 3 | 20% |
| CTRL | NIS | NSn | pilaar | pillar | 3 | 20% |
| CTRL | NIS | NSn | postzegel | stamp | 3 | 20% |
| CTRL | NIS | NSn | snijden | to cut | 3 | 20% |
| CTRL | TIS | NSn | spoelen | to flush or to rinse | 3 | 20% |
| CTRL | TIS | NSn | steil | steep | 3 | 20% |
| CTRL | NIS | NSn | stoel | chair | 3 | 20% |
| CTRL | NIS | NSn | stoelpoot | chair leg | 3 | 20% |
| CTRL | NIS | NSn | stoep | sidewalk | 3 | 20% |
| CTRL | TIS | NSn | stoep | sidewalk | 3 | 20% |
| CTRL | TIS | NSn | verdord | withered | 3 | 20% |
| CTRL | NIS | NSn | verleidster | temptress | 3 | 20% |
| CTRL | TIS | NSn | vreemde | stranger | 3 | 20% |
| CTRL | TIS | NSn | vuur | fire | 3 | 20% |
| CTRL | NIS | NSn | zuigen | to suck | 3 | 20% |
| CTRL | TIS | NSn | zuigen | to suck | 3 | 20% |
| CTRL | NIS | St | afblaffen | to bark at | 2 | 13% |
| CTRL | TIS | St | afkeer | aversion | 2 | 13% |
| CTRL | NIS | St | afsnauwen | to snap at | 2 | 13% |
| CTRL | TIS | St | armoede | poverty | 2 | 13% |
| CTRL | NIS | St | bedrog | deceit | 2 | 13% |
| CTRL | NIS | St | begraven | to bury | 2 | 13% |
| CTRL | NIS | St | buurman | neighbor | 2 | 13% |
| CTRL | TIS | St | chanteren | to blackmail | 2 | 13% |
| CTRL | NIS | St | diefstal | theft | 2 | 13% |
| CTRL | NIS | St | domkop | idiot | 2 | 13% |
| CTRL | TIS | St | doodsteek | deathblow | 2 | 13% |
| CTRL | NIS | St | dwang | coercion or force | 2 | 13% |
| CTRL | TIS | St | etiket | label | 2 | 13% |
| CTRL | TIS | St | getreiter | harassment | 2 | 13% |
| CTRL | TIS | St | haten | to hate | 2 | 13% |
| CTRL | TIS | St | hijgen | to pant | 2 | 13% |
| CTRL | TIS | St | hoer | whore | 2 | 13% |
| CTRL | TIS | St | hysterie | hysteria | 2 | 13% |
| CTRL | TIS | St | inbraak | burglary | 2 | 13% |
| CTRL | TIS | St | incest | incest | 2 | 13% |
| CTRL | NIS | St | kreng | bitch | 2 | 13% |
| CTRL | TIS | St | kreunen | to moan | 2 | 13% |
| CTRL | NIS | St | leugen | lie | 2 | 13% |
| CTRL | NIS | St | liegen | to lie | 2 | 13% |
| CTRL | TIS | St | martelen | to torture | 2 | 13% |
| CTRL | TIS | St | masker | mask | 2 | 13% |
| CTRL | TIS | St | messteek | knife stab | 2 | 13% |
| CTRL | TIS | St | misdaad | crime | 2 | 13% |
| CTRL | NIS | St | miskraam | miscarriage | 2 | 13% |
| CTRL | TIS | St | mislukt | failed | 2 | 13% |
| CTRL | TIS | St | moord | murder | 2 | 13% |
| CTRL | NIS | St | omkomen | to perish | 2 | 13% |
| CTRL | NIS | St | ongewenst | unwanted | 2 | 13% |
| CTRL | NIS | St | ontslag | resignation or dismissal | 2 | 13% |
| CTRL | TIS | St | ontslag | resignation or dismissal | 2 | 13% |
| CTRL | TIS | St | oom | uncle | 2 | 13% |
| CTRL | TIS | St | opsluiten | to lock up | 2 | 13% |
| CTRL | TIS | St | piemel | willy | 2 | 13% |
| CTRL | TIS | St | schande | shame | 2 | 13% |
| CTRL | TIS | St | slet | slut | 2 | 13% |
| CTRL | TIS | St | sterven | to die | 2 | 13% |
| CTRL | TIS | St | trappen | to kick | 2 | 13% |
| CTRL | TIS | St | treiteren | to harass | 2 | 13% |
| CTRL | NIS | St | uitlachen | to laugh at | 2 | 13% |
| CTRL | TIS | St | vastbinden | to tie | 2 | 13% |
| CTRL | TIS | St | verdrinken | to drown | 2 | 13% |
| CTRL | NIS | St | verzuipen | to drown | 2 | 13% |
| CTRL | TIS | St | vies | dirty | 2 | 13% |
| CTRL | TIS | St | walging | disgust | 2 | 13% |
| CTRL | NIS | St | wanhoop | despair | 2 | 13% |
| CTRL | TIS | St | wraak | revenge | 2 | 13% |
| CTRL | TIS | St | wurgen | to strangle | 2 | 13% |
| CTRL | NIS | St | zeer | ache | 2 | 13% |
| CTRL | TIS | St | zondebok | scapegoat | 2 | 13% |
| CTRL | NIS | NSt | afhakken | to chop off | 2 | 13% |
| CTRL | TIS | NSt | afhakken | to chop off | 2 | 13% |
| CTRL | NIS | NSt | afkeer | aversion | 2 | 13% |
| CTRL | TIS | NSt | afschuw | revulsion | 2 | 13% |
| CTRL | TIS | NSt | afsnauwen | to snap at | 2 | 13% |
| CTRL | NIS | NSt | afwijzing | rejection | 2 | 13% |
| CTRL | NIS | NSt | bang | afraid | 2 | 13% |
| CTRL | NIS | NSt | bedrog | deceit | 2 | 13% |
| CTRL | NIS | NSt | begraven | to bury | 2 | 13% |
| CTRL | NIS | NSt | besmetten | to contaminate | 2 | 13% |
| CTRL | TIS | NSt | blinddoek | blindfold | 2 | 13% |
| CTRL | TIS | NSt | boos | angry | 2 | 13% |
| CTRL | TIS | NSt | depressie | depression | 2 | 13% |
| CTRL | TIS | NSt | doodgaan | to die | 2 | 13% |
| CTRL | NIS | NSt | etter | pus | 2 | 13% |
| CTRL | TIS | NSt | etter | pus | 2 | 13% |
| CTRL | NIS | NSt | getreiter | harassment | 2 | 13% |
| CTRL | NIS | NSt | inbraak | burglary | 2 | 13% |
| CTRL | NIS | NSt | instorten | to collapse | 2 | 13% |
| CTRL | NIS | NSt | kreng | bitch | 2 | 13% |
| CTRL | NIS | NSt | kwetsen | to hurt | 2 | 13% |
| CTRL | TIS | NSt | leugen | lie | 2 | 13% |
| CTRL | TIS | NSt | meeloper | opportunist | 2 | 13% |
| CTRL | NIS | NSt | mislukt | failed | 2 | 13% |
| CTRL | TIS | NSt | misvormen | to deform | 2 | 13% |
| CTRL | NIS | NSt | onrecht | injustice | 2 | 13% |
| CTRL | TIS | NSt | onrecht | injustice | 2 | 13% |
| CTRL | NIS | NSt | schaden | to damage | 2 | 13% |
| CTRL | TIS | NSt | schande | shame | 2 | 13% |
| CTRL | TIS | NSt | schoft | bastard | 2 | 13% |
| CTRL | TIS | NSt | schoppen | to kick | 2 | 13% |
| CTRL | NIS | NSt | slecht | bad | 2 | 13% |
| CTRL | TIS | NSt | sterven | to die | 2 | 13% |
| CTRL | NIS | NSt | stinken | to stink | 2 | 13% |
| CTRL | TIS | NSt | uitgescholden | scolded | 2 | 13% |
| CTRL | NIS | NSt | uitjouwen | to boo | 2 | 13% |
| CTRL | TIS | NSt | uitjouwen | to boo | 2 | 13% |
| CTRL | TIS | NSt | verleidster | temptress | 2 | 13% |
| CTRL | TIS | NSt | vernielen | to vandalize | 2 | 13% |
| CTRL | TIS | NSt | wanhoop | despair | 2 | 13% |
| CTRL | NIS | NSt | woest | enraged | 2 | 13% |
| CTRL | TIS | NSt | woest | enraged | 2 | 13% |
| CTRL | TIS | NSn | achterlaten | leave behind | 2 | 13% |
| CTRL | NIS | NSn | atoombom | nuclear bomb | 2 | 13% |
| CTRL | TIS | NSn | bad | bath | 2 | 13% |
| CTRL | NIS | NSn | begraven | to bury | 2 | 13% |
| CTRL | TIS | NSn | bloot | naked | 2 | 13% |
| CTRL | NIS | NSn | broer | brother | 2 | 13% |
| CTRL | TIS | NSn | buurman | neighbor | 2 | 13% |
| CTRL | NIS | NSn | chauffeur | driver | 2 | 13% |
| CTRL | NIS | NSn | domkop | idiot | 2 | 13% |
| CTRL | TIS | NSn | domkop | idiot | 2 | 13% |
| CTRL | TIS | NSn | embryo | embryo | 2 | 13% |
| CTRL | TIS | NSn | folteren | to torture | 2 | 13% |
| CTRL | NIS | NSn | hagedis | lizard | 2 | 13% |
| CTRL | TIS | NSn | hagedis | lizard | 2 | 13% |
| CTRL | NIS | NSn | handen | hands | 2 | 13% |
| CTRL | NIS | NSn | hijgen | to pant | 2 | 13% |
| CTRL | TIS | NSn | hijgen | to pant | 2 | 13% |
| CTRL | TIS | NSn | juni | June | 2 | 13% |
| CTRL | TIS | NSn | kader | framework | 2 | 13% |
| CTRL | NIS | NSn | kogel | bullet | 2 | 13% |
| CTRL | TIS | NSn | kozijn | window frame | 2 | 13% |
| CTRL | TIS | NSn | krabben | to scratch | 2 | 13% |
| CTRL | TIS | NSn | martelen | to torture | 2 | 13% |
| CTRL | TIS | NSn | mes | knife | 2 | 13% |
| CTRL | NIS | NSn | misvormen | to deform | 2 | 13% |
| CTRL | TIS | NSn | misvormen | to deform | 2 | 13% |
| CTRL | TIS | NSn | moord | murder | 2 | 13% |
| CTRL | TIS | NSn | ogen | eyes | 2 | 13% |
| CTRL | NIS | NSn | ontslag | resignation or dismissal | 2 | 13% |
| CTRL | NIS | NSn | oom | uncle | 2 | 13% |
| CTRL | TIS | NSn | opsluiten | to lock up | 2 | 13% |
| CTRL | NIS | NSn | orgie | orgy | 2 | 13% |
| CTRL | NIS | NSn | pasen | Easter | 2 | 13% |
| CTRL | TIS | NSn | piemel | willy | 2 | 13% |
| CTRL | TIS | NSn | regel | rule | 2 | 13% |
| CTRL | TIS | NSn | schoft | bastard | 2 | 13% |
| CTRL | TIS | NSn | stank | stench | 2 | 13% |
| CTRL | NIS | NSn | steil | steep | 2 | 13% |
| CTRL | TIS | NSn | stoel | chair | 2 | 13% |
| CTRL | TIS | NSn | stoelpoot | chair leg | 2 | 13% |
| CTRL | NIS | NSn | trappen | to kick | 2 | 13% |
| CTRL | TIS | NSn | treden | to step | 2 | 13% |
| CTRL | TIS | NSn | vergroten | enlarge | 2 | 13% |
| CTRL | TIS | NSn | verlammen | to paralyze | 2 | 13% |
| CTRL | NIS | NSn | vuur | fire | 2 | 13% |
| CTRL | NIS | St | achterlaten | leave behind | 1 | 7% |
| CTRL | TIS | St | afgrijzen | horror | 1 | 7% |
| CTRL | NIS | St | afgunst | envy | 1 | 7% |
| CTRL | TIS | St | afgunst | envy | 1 | 7% |
| CTRL | NIS | St | afkeer | aversion | 1 | 7% |
| CTRL | TIS | St | afschuw | revulsion | 1 | 7% |
| CTRL | NIS | St | agressie | aggression | 1 | 7% |
| CTRL | NIS | St | armoede | poverty | 1 | 7% |
| CTRL | TIS | St | baby | baby | 1 | 7% |
| CTRL | NIS | St | bedrieger | deceiver | 1 | 7% |
| CTRL | NIS | St | belazerd | fooled | 1 | 7% |
| CTRL | TIS | St | bestek | cutlery | 1 | 7% |
| CTRL | TIS | St | buurman | neighbor | 1 | 7% |
| CTRL | NIS | St | dakgoot | gutter | 1 | 7% |
| CTRL | TIS | St | deurknop | door knob | 1 | 7% |
| CTRL | TIS | St | document | document | 1 | 7% |
| CTRL | TIS | St | dokter | (general practice) doctor | 1 | 7% |
| CTRL | TIS | St | domkop | idiot | 1 | 7% |
| CTRL | TIS | St | doodslag | manslaughter | 1 | 7% |
| CTRL | NIS | St | dubbel | double | 1 | 7% |
| CTRL | NIS | St | ellende | misery | 1 | 7% |
| CTRL | TIS | St | ergeren | to annoy | 1 | 7% |
| CTRL | TIS | St | ergernis | annoyance | 1 | 7% |
| CTRL | TIS | St | folteren | to torture | 1 | 7% |
| CTRL | NIS | St | hagedis | lizard | 1 | 7% |
| CTRL | NIS | St | hijgen | to pant | 1 | 7% |
| CTRL | NIS | St | huisdier | pet | 1 | 7% |
| CTRL | TIS | St | huisdier | pet | 1 | 7% |
| CTRL | NIS | St | ijzer | iron | 1 | 7% |
| CTRL | NIS | St | instorten | to collapse | 1 | 7% |
| CTRL | TIS | St | juni | June | 1 | 7% |
| CTRL | NIS | St | kelder | basement | 1 | 7% |
| CTRL | TIS | St | kelder | basement | 1 | 7% |
| CTRL | NIS | St | kerk | church | 1 | 7% |
| CTRL | TIS | St | kerk | church | 1 | 7% |
| CTRL | NIS | St | knijpen | to pinch | 1 | 7% |
| CTRL | TIS | St | kogel | bullet | 1 | 7% |
| CTRL | TIS | St | kotsen | to vomit | 1 | 7% |
| CTRL | TIS | St | kou | cold | 1 | 7% |
| CTRL | TIS | St | kreng | bitch | 1 | 7% |
| CTRL | TIS | St | kuthoer | pussy whore | 1 | 7% |
| CTRL | NIS | St | lafaard | coward | 1 | 7% |
| CTRL | TIS | St | man | man | 1 | 7% |
| CTRL | NIS | St | masker | mask | 1 | 7% |
| CTRL | NIS | St | meeloper | opportunist | 1 | 7% |
| CTRL | TIS | St | meeloper | opportunist | 1 | 7% |
| CTRL | NIS | St | mes | knife | 1 | 7% |
| CTRL | NIS | St | metaal | metal | 1 | 7% |
| CTRL | NIS | St | moedeloos | despondent | 1 | 7% |
| CTRL | TIS | St | moeder | mother | 1 | 7% |
| CTRL | TIS | St | mond | mouth | 1 | 7% |
| CTRL | NIS | St | ogen | eyes | 1 | 7% |
| CTRL | TIS | St | ogen | eyes | 1 | 7% |
| CTRL | NIS | St | ongeluk | accident | 1 | 7% |
| CTRL | NIS | St | ongeval | accident | 1 | 7% |
| CTRL | NIS | St | ontrouw | unfaithful | 1 | 7% |
| CTRL | TIS | St | oorlog | war | 1 | 7% |
| CTRL | NIS | St | oplichten | to scam | 1 | 7% |
| CTRL | NIS | St | opsluiten | to lock up | 1 | 7% |
| CTRL | NIS | St | pasen | Easter | 1 | 7% |
| CTRL | TIS | St | plafond | ceiling | 1 | 7% |
| CTRL | NIS | St | razernij | fury | 1 | 7% |
| CTRL | NIS | St | schande | shame | 1 | 7% |
| CTRL | TIS | St | slaaf | slave | 1 | 7% |
| CTRL | TIS | St | slecht | bad | 1 | 7% |
| CTRL | TIS | St | slijmen | suck up to | 1 | 7% |
| CTRL | TIS | St | spoelen | to flush or to rinse | 1 | 7% |
| CTRL | NIS | St | stank | stench | 1 | 7% |
| CTRL | TIS | St | stank | stench | 1 | 7% |
| CTRL | NIS | St | steekwond | stab wound | 1 | 7% |
| CTRL | TIS | St | steekwond | stab wound | 1 | 7% |
| CTRL | NIS | St | steil | steep | 1 | 7% |
| CTRL | NIS | St | stiekem | secretly | 1 | 7% |
| CTRL | NIS | St | stinken | to stink | 1 | 7% |
| CTRL | NIS | St | stomerij | drycleaner | 1 | 7% |
| CTRL | TIS | St | touw | rope | 1 | 7% |
| CTRL | NIS | St | treiteren | to harass | 1 | 7% |
| CTRL | NIS | St | uitgescholden | scolded | 1 | 7% |
| CTRL | TIS | St | uitjouwen | to boo | 1 | 7% |
| CTRL | NIS | St | vader | father | 1 | 7% |
| CTRL | NIS | St | verbranden | to burn | 1 | 7% |
| CTRL | NIS | St | verdrinken | to drown | 1 | 7% |
| CTRL | NIS | St | verkrachting | rape | 1 | 7% |
| CTRL | TIS | St | verleidster | temptress | 1 | 7% |
| CTRL | NIS | St | vies | dirty | 1 | 7% |
| CTRL | NIS | St | vreemde | stranger | 1 | 7% |
| CTRL | TIS | St | zetel | seat | 1 | 7% |
| CTRL | TIS | St | zeuren | to nag | 1 | 7% |
| CTRL | NIS | St | zuigen | to suck | 1 | 7% |
| CTRL | TIS | St | zuigen | to suck | 1 | 7% |
| CTRL | NIS | NSt | achterlaten | leave behind | 1 | 7% |
| CTRL | TIS | NSt | achterlaten | leave behind | 1 | 7% |
| CTRL | NIS | NSt | afblaffen | to bark at | 1 | 7% |
| CTRL | TIS | NSt | afgrijzen | horror | 1 | 7% |
| CTRL | TIS | NSt | afkraken | to decry | 1 | 7% |
| CTRL | NIS | NSt | afschuw | revulsion | 1 | 7% |
| CTRL | TIS | NSt | afwijzing | rejection | 1 | 7% |
| CTRL | NIS | NSt | alleen | alone | 1 | 7% |
| CTRL | NIS | NSt | angst | fear | 1 | 7% |
| CTRL | TIS | NSt | bang | afraid | 1 | 7% |
| CTRL | TIS | NSt | bedrog | deceit | 1 | 7% |
| CTRL | TIS | NSt | begraven | to bury | 1 | 7% |
| CTRL | TIS | NSt | belazerd | fooled | 1 | 7% |
| CTRL | TIS | NSt | besmetten | to contaminate | 1 | 7% |
| CTRL | NIS | NSt | boos | angry | 1 | 7% |
| CTRL | NIS | NSt | bordeel | brothel | 1 | 7% |
| CTRL | NIS | NSt | branden | to burn | 1 | 7% |
| CTRL | NIS | NSt | conflict | conflict | 1 | 7% |
| CTRL | NIS | NSt | depressie | depression | 1 | 7% |
| CTRL | NIS | NSt | diefstal | theft | 1 | 7% |
| CTRL | NIS | NSt | domkop | idiot | 1 | 7% |
| CTRL | NIS | NSt | dreigen | to threaten | 1 | 7% |
| CTRL | TIS | NSt | dreigen | to threaten | 1 | 7% |
| CTRL | TIS | NSt | dwang | coercion or force | 1 | 7% |
| CTRL | NIS | NSt | ergeren | to annoy | 1 | 7% |
| CTRL | TIS | NSt | ergeren | to annoy | 1 | 7% |
| CTRL | NIS | NSt | ergernis | annoyance | 1 | 7% |
| CTRL | TIS | NSt | ergernis | annoyance | 1 | 7% |
| CTRL | TIS | NSt | falen | to fail | 1 | 7% |
| CTRL | TIS | NSt | geweld | violence | 1 | 7% |
| CTRL | TIS | NSt | hijgen | to pant | 1 | 7% |
| CTRL | TIS | NSt | instorten | to collapse | 1 | 7% |
| CTRL | NIS | NSt | knijpen | to pinch | 1 | 7% |
| CTRL | TIS | NSt | knijpen | to pinch | 1 | 7% |
| CTRL | TIS | NSt | kou | cold | 1 | 7% |
| CTRL | TIS | NSt | krabben | to scratch | 1 | 7% |
| CTRL | NIS | NSt | mes | knife | 1 | 7% |
| CTRL | TIS | NSt | middel | middle | 1 | 7% |
| CTRL | TIS | NSt | mislukt | failed | 1 | 7% |
| CTRL | NIS | NSt | misvormen | to deform | 1 | 7% |
| CTRL | TIS | NSt | moedeloos | despondent | 1 | 7% |
| CTRL | NIS | NSt | noodkreet | cry for help | 1 | 7% |
| CTRL | TIS | NSt | noodkreet | cry for help | 1 | 7% |
| CTRL | TIS | NSt | ongewenst | unwanted | 1 | 7% |
| CTRL | NIS | NSt | onmacht | powerlessness | 1 | 7% |
| CTRL | NIS | NSt | ontslag | resignation or dismissal | 1 | 7% |
| CTRL | NIS | NSt | orgie | orgy | 1 | 7% |
| CTRL | TIS | NSt | pijn | pain | 1 | 7% |
| CTRL | TIS | NSt | ruzie | fight or quarrel | 1 | 7% |
| CTRL | TIS | NSt | schaden | to damage | 1 | 7% |
| CTRL | NIS | NSt | schok | shock | 1 | 7% |
| CTRL | NIS | NSt | stank | stench | 1 | 7% |
| CTRL | NIS | NSt | steil | steep | 1 | 7% |
| CTRL | NIS | NSt | tegenslag | setback | 1 | 7% |
| CTRL | TIS | NSt | tegenslag | setback | 1 | 7% |
| CTRL | TIS | NSt | telegram | telegram | 1 | 7% |
| CTRL | NIS | NSt | trappen | to kick | 1 | 7% |
| CTRL | NIS | NSt | uitgescholden | scolded | 1 | 7% |
| CTRL | NIS | NSt | uitlachen | to laugh at | 1 | 7% |
| CTRL | NIS | NSt | vastbinden | to tie | 1 | 7% |
| CTRL | NIS | NSt | verbranden | to burn | 1 | 7% |
| CTRL | TIS | NSt | verbranden | to burn | 1 | 7% |
| CTRL | NIS | NSt | verdord | withered | 1 | 7% |
| CTRL | NIS | NSt | verleidster | temptress | 1 | 7% |
| CTRL | NIS | NSt | vernielen | to vandalize | 1 | 7% |
| CTRL | TIS | NSt | verraad | betrayal | 1 | 7% |
| CTRL | TIS | NSt | vierkant | square | 1 | 7% |
| CTRL | TIS | NSt | wanhopen | to despair | 1 | 7% |
| CTRL | NIS | NSt | wraak | revenge | 1 | 7% |
| CTRL | TIS | NSt | zetel | seat | 1 | 7% |
| CTRL | NIS | NSt | zondebok | scapegoat | 1 | 7% |
| CTRL | NIS | NSn | aanslag | attack | 1 | 7% |
| CTRL | TIS | NSn | aanslag | attack | 1 | 7% |
| CTRL | TIS | NSn | afblaffen | to bark at | 1 | 7% |
| CTRL | NIS | NSn | afhakken | to chop off | 1 | 7% |
| CTRL | NIS | NSn | afkeer | aversion | 1 | 7% |
| CTRL | NIS | NSn | afschuw | revulsion | 1 | 7% |
| CTRL | TIS | NSn | afschuw | revulsion | 1 | 7% |
| CTRL | NIS | NSn | armoede | poverty | 1 | 7% |
| CTRL | TIS | NSn | armoede | poverty | 1 | 7% |
| CTRL | NIS | NSn | bad | bath | 1 | 7% |
| CTRL | NIS | NSn | bang | afraid | 1 | 7% |
| CTRL | NIS | NSn | bed | bed | 1 | 7% |
| CTRL | NIS | NSn | bedrog | deceit | 1 | 7% |
| CTRL | TIS | NSn | begraven | to bury | 1 | 7% |
| CTRL | TIS | NSn | beklemmen | to oppress | 1 | 7% |
| CTRL | NIS | NSn | bordeel | brothel | 1 | 7% |
| CTRL | TIS | NSn | branden | to burn | 1 | 7% |
| CTRL | NIS | NSn | buurman | neighbor | 1 | 7% |
| CTRL | TIS | NSn | chanteren | to blackmail | 1 | 7% |
| CTRL | NIS | NSn | conflict | conflict | 1 | 7% |
| CTRL | TIS | NSn | crimineel | criminal | 1 | 7% |
| CTRL | NIS | NSn | depressie | depression | 1 | 7% |
| CTRL | TIS | NSn | diefstal | theft | 1 | 7% |
| CTRL | NIS | NSn | document | document | 1 | 7% |
| CTRL | TIS | NSn | document | document | 1 | 7% |
| CTRL | NIS | NSn | dokter | (general practice) doctor | 1 | 7% |
| CTRL | TIS | NSn | dokter | (general practice) doctor | 1 | 7% |
| CTRL | TIS | NSn | doodsteek | deathblow | 1 | 7% |
| CTRL | TIS | NSn | doorslikken | to swallow | 1 | 7% |
| CTRL | NIS | NSn | droevig | sad | 1 | 7% |
| CTRL | TIS | NSn | dubbel | double | 1 | 7% |
| CTRL | TIS | NSn | dwang | coercion or force | 1 | 7% |
| CTRL | NIS | NSn | ergernis | annoyance | 1 | 7% |
| CTRL | TIS | NSn | ergernis | annoyance | 1 | 7% |
| CTRL | TIS | NSn | etiket | label | 1 | 7% |
| CTRL | NIS | NSn | etter | pus | 1 | 7% |
| CTRL | NIS | NSn | folteren | to torture | 1 | 7% |
| CTRL | TIS | NSn | gebouw | building | 1 | 7% |
| CTRL | TIS | NSn | gezwel | tumor | 1 | 7% |
| CTRL | TIS | NSn | gijzeling | kidnapping | 1 | 7% |
| CTRL | TIS | NSn | hysterie | hysteria | 1 | 7% |
| CTRL | TIS | NSn | inbraak | burglary | 1 | 7% |
| CTRL | NIS | NSn | incest | incest | 1 | 7% |
| CTRL | TIS | NSn | incest | incest | 1 | 7% |
| CTRL | NIS | NSn | juni | June | 1 | 7% |
| CTRL | NIS | NSn | knijpen | to pinch | 1 | 7% |
| CTRL | TIS | NSn | kogel | bullet | 1 | 7% |
| CTRL | NIS | NSn | kou | cold | 1 | 7% |
| CTRL | NIS | NSn | krabben | to scratch | 1 | 7% |
| CTRL | TIS | NSn | krenken | to hurt | 1 | 7% |
| CTRL | TIS | NSn | kreunen | to moan | 1 | 7% |
| CTRL | NIS | NSn | lafaard | coward | 1 | 7% |
| CTRL | TIS | NSn | lafaard | coward | 1 | 7% |
| CTRL | TIS | NSn | legpuzzel | jigsaw puzzle | 1 | 7% |
| CTRL | TIS | NSn | lepra | leprosy | 1 | 7% |
| CTRL | NIS | NSn | magazijn | warehouse | 1 | 7% |
| CTRL | NIS | NSn | masker | mask | 1 | 7% |
| CTRL | NIS | NSn | meeloper | opportunist | 1 | 7% |
| CTRL | NIS | NSn | miskraam | miscarriage | 1 | 7% |
| CTRL | NIS | NSn | mislukt | failed | 1 | 7% |
| CTRL | TIS | NSn | mislukt | failed | 1 | 7% |
| CTRL | NIS | NSn | mismaakt | deformed | 1 | 7% |
| CTRL | TIS | NSn | mismaakt | deformed | 1 | 7% |
| CTRL | TIS | NSn | moeder | mother | 1 | 7% |
| CTRL | NIS | NSn | mond | mouth | 1 | 7% |
| CTRL | NIS | NSn | noodkreet | cry for help | 1 | 7% |
| CTRL | TIS | NSn | noodkreet | cry for help | 1 | 7% |
| CTRL | NIS | NSn | ogen | eyes | 1 | 7% |
| CTRL | NIS | NSn | ongeval | accident | 1 | 7% |
| CTRL | TIS | NSn | ongeval | accident | 1 | 7% |
| CTRL | NIS | NSn | onmacht | powerlessness | 1 | 7% |
| CTRL | NIS | NSn | opsluiten | to lock up | 1 | 7% |
| CTRL | NIS | NSn | pedofiel | paedophile | 1 | 7% |
| CTRL | TIS | NSn | pedofiel | paedophile | 1 | 7% |
| CTRL | NIS | NSn | regel | rule | 1 | 7% |
| CTRL | TIS | NSn | roofmoord | robbery with murder | 1 | 7% |
| CTRL | TIS | NSn | sadist | sadist | 1 | 7% |
| CTRL | TIS | NSn | schaden | to damage | 1 | 7% |
| CTRL | NIS | NSn | schande | shame | 1 | 7% |
| CTRL | TIS | NSn | schande | shame | 1 | 7% |
| CTRL | NIS | NSn | schok | shock | 1 | 7% |
| CTRL | TIS | NSn | schoppen | to kick | 1 | 7% |
| CTRL | NIS | NSn | schuldig | guilty | 1 | 7% |
| CTRL | NIS | NSn | seks | sex | 1 | 7% |
| CTRL | TIS | NSn | slecht | bad | 1 | 7% |
| CTRL | NIS | NSn | slet | slut | 1 | 7% |
| CTRL | NIS | NSn | stank | stench | 1 | 7% |
| CTRL | TIS | NSn | steekwond | stab wound | 1 | 7% |
| CTRL | TIS | NSn | sterven | to die | 1 | 7% |
| CTRL | NIS | NSn | stiekem | secretly | 1 | 7% |
| CTRL | TIS | NSn | stiekem | secretly | 1 | 7% |
| CTRL | TIS | NSn | stikken | to suffocate | 1 | 7% |
| CTRL | TIS | NSn | stinken | to stink | 1 | 7% |
| CTRL | TIS | NSn | trappen | to kick | 1 | 7% |
| CTRL | NIS | NSn | trauma | trauma | 1 | 7% |
| CTRL | NIS | NSn | vader | father | 1 | 7% |
| CTRL | NIS | NSn | vastbinden | to tie | 1 | 7% |
| CTRL | TIS | NSn | vastbinden | to tie | 1 | 7% |
| CTRL | NIS | NSn | verdord | withered | 1 | 7% |
| CTRL | NIS | NSn | vergroten | enlarge | 1 | 7% |
| CTRL | NIS | NSn | verraad | betrayal | 1 | 7% |
| CTRL | NIS | NSn | woest | enraged | 1 | 7% |
| CTRL | NIS | NSn | zeer | ache | 1 | 7% |

TrialType = word presented in the trial (in Dutch), eText = English translation of the word, n = number of participants who rated this word highly, prop = percentage of participants who rated highly this word out of total individuals in the Group, St = self-relevant trauma-related, NSt = non-self-relevant trauma-related, NSn = Non-self-relevant neutral, NIS = neutral identity state, TIS = trauma-related identity state, DID-G = individuals with a diagnosis of dissociative identity disorder (DID), that is genuine DID, DID-S = DID-simulating controls, CTRL = a paired control group of healthy participants (controls for the NIS) and individuals with a diagnosis of PTSD (controls for the TIS)

^n1 = 14

^n2 = 14

^n3 = 15

**Appendix B: Supplementary findings on activation of individual brain regions.**

**Results**

***Main effect of consciousness***

In the diagnosed DID (DID-G) participant group, decreased brain activation was observed in the left middle temporal gyrus, cingulate gyrus and bilateral fusiform gyrus. Regarding subcortical areas, increased activation was observed in the right lateral-dorsal part of the caudate, only between the DID-G and paired control (CTRL) groups. Decreased activation was observed in the right putamen and right thalamus, as well as in the left tail of the caudate nucleus, when comparing between the DID-G and simulating DID (DID-S) groups (see Table 2).

***Self-relevance effect [self-relevant (St) versus non-self-relevant (NSt) trauma-related words]***

**Within-group comparisons**

Increased brain activation was observed in the right angular gyrus and the right dorsal part of the caudate nucleus in the DID-G group when in the trauma-avoidant identity state, and in the right angular gyrus, left middle temporal gyrus and bilateral cingulate gyrus when in the trauma-related identity state (see Table 3). In the overt processing block, increased brain activation was found in the right cingulate gyrus and the right dorsal part of caudate nucleus. Decreased activation was found in the left lingual gyrus, in the overt processing block, and in the left calcarine sulcus, in the covert processing block.

**Between-group comparisons**

Comparisons between the DID-G and CTRL groups showed increased brain activation was found for the left middle cingulate gyrus in the trauma-related identity state. Additionally, increased activation during ratings of self-relevant trauma-related (St) words was found in the left fusiform gyrus and the left dorsal part of caudate nucleus, only in the covert processing block. Decreased activation of the left calcarine sulcus was also found for the covert processing SCP block (see Table 3).

Neural data comparisons between the DID-G and DID-S groups showed increased activation in the right cingulate, right angular and right lingual gyri, when in the trauma-avoidant identity state, whereas decreased activation was observed in the left cingulate gyrus and right putamen when in the trauma-related identity state. The right cingulate and right angular gyri where also activated during the overt processing block, in addition to the subcortical regions of the right dorsal part of caudate nucleus and right thalamus. Decreased activation was observed in the left fusiform gyrus in the overt processing block, and in the left brain stem in the covert processing block (see Table 3).

***Emotional intensity effect [trauma-related (NSt) versus neutral (NSn) non-self-relevant words]***

**Within-group comparisons**

Increased activation was observed in the middle cingulate gyrus and left angular gyrus, only in the trauma-related identity state (see Table 4). For subcortical areas, increased activation was found in the bilateral thalamus and left ventral diencephalon. Increase in activation was also observed in the bilateral angular and bilateral cingulate gyri, in the covert processing block. The right tail of the caudate nucleus showed decreased activation in the covert processing block (see Table 4).

**Between-group comparisons**

Neural data comparisons between the DID-G and CTRL groups showed increased activation in the left angular gyrus, left lingual gyrus, right cingulate sulcus, and in the subcortical regions of the bilateral thalamus, right putamen, left ventral diencephalon and left cerebellar vermal lobules I-V, when in the trauma-related identity state. The bilateral cingulate gyrus was activated during covert processing (see Table 4).

Comparisons between the DID-G and DID-S groups showed increased activation in the right and bilateral middle cingulate gyrus, and in the subcortical regions of the left thalamus, right hippocampus and bilateral lateral ventrical, mainly in the trauma-related identity state. In the overt processing block, increased activity was found in the left tail of the caudate nucleus, whereas in the covert processing block, increase in activation was observed in the bilateral cingulate gyrus and the right thalamus (see Table 4).

**Discussion**

Increased activation was observed in the bilateral angular gyrus during both overt and covert self-relevance and emotional intensity processing, in both the trauma-avoidant and the trauma-related identity states of individuals diagnosed with DID. This finding supports prior research suggesting this region’s involvement in the cognitive processing of self-relevant trauma-related stimuli (4,5). Roydeva and Reinders (6) have also emphasised the role of the angular gyrus as a diagnostic neurofunctional biomarker of pathological dissocation, in line with the neurobiological model for severe pathological dissociation (5). Morphological alterations of the angular region has also been previously observed (7).

In addition, increased brain activation during both overt and covert processing, and in both identity states of individuals with DID, was also observed in the cingulate gyrus, in line with previous findings (4,7–10). The cingulate cortex has been previously found to be activated during retrieval of emotional autobiographical knowledge (11), while it has also been shown to be involved in memory and emotion (12), as well as in inter-identity avoidance of trauma-related knowledge (3). Combined with the increased frontal activation, this outcome further supports the hypoarousal of individuals with DID to trauma-related and emotional stimuli during overt processing (5). Of note, this region has also been previously proposed by Roydeva and Reinders (6) as a biomarker of dissociation.

Moreover, the temporal gyrus was also found to be activated during both overt and covert processing, as well as in both dissociative identity states of individuals with DID, in line with earlier research (4,5,9). Abnormal activation of the temporal regions has been previously implicated in dissociative amnesia (13) and avoidance of trauma-related knowledge (3), in dissociative disorders and DID (8,14), as well as in pathological dissociation in general (15). The temporal areas have also been found to interact with the frontal and parietal gyri during episodic memory processes (16), indicating these regions’ central role in the processing of self-relevant trauma-related knowledge in individuals with DID. A previous study also showed temporal activation changes during the switch between dissociative identity states of individuals with DID (17). Temporal regions have also been implicated in the Triple Network Model of Pathological Dissociation proposed by Lebois and colleagues (18). Further, Schlumpf and colleagues (19) found increased activity in the temporal gyrus during covert presentation of angry and neutral faces, especially in the trauma-related identity state of participants with DID.

Regarding regions overlapping both the temporal and occipital gyri, the fusiform gyrus was mainly activated during covert self-relevance processing, independent of dissociative identity state, whereas the lingual gyrus was mostly activated when the individuals with DID were in both the trauma-avoidant and trauma-related identity states, independent of overt or covert exposure. These findings are in line with earlier research (4,5,7,9,10), and paired with the increased activation of the occipital gyrus and the calcarine sulcus during covert exposure of trauma-related words, especially in the trauma-related identity state, they suggest an involvement of these areas in DID. Furthermore, the lingual gyrus (20) has been implicated in the manifestation of the dissociative subtype of PTSD (post-traumatic stress disorder). Although the occipital cortex is not as often reported in the literature of DID and dissociation, it has been previously found to be activated in individuals with DID (21), especially after exposure to individualized trauma-related scripts (5), as well as in dissociative PTSD (22). These outcomes correspond with earlier findings implying an association of the fusiform, lingual and occipital regions with perceptual memory processes (23) and perceptual awareness (4). These areas form the posterior association areas (4).

Furthermore, increased activation of the insula was observed during covert exposure of trauma-related words in both the trauma-avoidant and trauma-related identity states. In line with our findings, the insula has been previously found to be activated both in the trauma-avoidant (15) and in the trauma-related identity state of individuals with DID following exposure to individualized trauma-related scripts (4,5,15). The insular region is involved in emotional regulation (7), as well as in the individual’s emotional response to distressing stimuli (9) and has been previously reported to be abnormally activated in dissociative disorders (8), and in dissociative PTSD (20,24).

Interestingly, regarding activation of subcortical areas, our findings extrapolate earlier studies (3–5,25) indicating that, among others, the caudate nucleus is involved in trauma-related processing in DID. Our outcomes showed that the dorsal part of the caudate was activated during both overt and covert self-relevance processing, especially in the trauma-avoidant identity state, whereas the caudate’s tail was mainly activated during overt processing of trauma-related words. The caudate nucleus is involved in movement processes, as well as in anxiety (9), and, as part of the dorsal striatum, it has been associated with trait dissociation and switching between dissociative identity states (5), while it has also been recommended as a neurofunctional biomarker of dissociation (15). Our outcomes further showed increased activation in the ventral diencephalon (9) in individuals with DID when in the trauma-related identity state, an area which has also been implicated in the process of identity state switching in individuals with DID (5). Moreover, the thalamus was found to be activated during both overt and covert processing, especially in the trauma-related identity state, in line with previous findings (3,4,9). The thalamus has been previously proposed as a potential biomarker of dissociation (15). Lastly, increased activation was found in the putamen (9,10), the cerebellar vermal lobules I-V, and the lateral ventrical when the individuals with DID were in the trauma-related identity state. These findings were independent of overt or covert exposure to the stimuli. Of note, a study by Dimitrova and colleagues (3) found decreased activation of the putamen, contrasting our findings.

**REFERENCES**

1. Dimitrova LI, Vissia EM, Geugies H, Hofstetter H, Chalavi S, Reinders AATS. No Self Without Salience: Affective and Self-relevance Ratings of 552 Emotionally Valenced and Neutral Dutch Words. J Psycholinguist Res [Internet]. 2022;51(1):17–32. Available from: https://doi.org/10.1007/s10936-021-09784-1

2. Strouza AI, Lawrence AJ, Vissia EM, Kakouris A, Akan A, Nijenhuis ERS, et al. Identity State-Dependent Self-Relevance and Emotional Intensity Ratings of Words in Dissociative Identity Disorder: A Controlled Longitudinal Study. Brain Behav. 2023;13(10).

3. Dimitrova LI, Lawrence AJ, Vissia EM, Chalavi S, Kakouris AF, Veltman DJ, et al. Inter-identity amnesia in dissociative identity disorder resolved: A behavioural and neurobiological study. J Psychiatr Res. 2024 Jun 1;174:220–9.

4. Reinders AATS, Willemsen ATM, Vos HPJ, den Boer JA, Nijenhuis ERS. Fact or factitious? A psychobiological study of authentic and simulated dissociative identity states. Laks J, editor. PLoS One [Internet]. 2012 Jun;7(6):e39279. Available from: http://dx.plos.org/10.1371/journal.pone.0039279

5. Reinders AATS, Willemsen ATM, den Boer JA, Vos HPJ, Veltman DJ, Loewenstein RJ. Opposite brain emotion-regulation patterns in identity states of dissociative identity disorder: A PET study and neurobiological model. Psychiatry Res [Internet]. 2014 Sep 30;223(3):236–43. Available from: http://www.ncbi.nlm.nih.gov/pubmed/24976633

6. Roydeva MI, Reinders AATS. Biomarkers of Pathological Dissociation: A Systematic Review. Neurosci Biobehav Rev [Internet]. 2021;123(November 2020):120–202. Available from: https://doi.org/10.1016/j.neubiorev.2020.11.019

7. Reinders AATS, Chalavi S, Schlumpf YR, Vissia EM, Nijenhuis ERS, Jäncke L, et al. Neurodevelopmental origins of abnormal cortical morphology in dissociative identity disorder. Acta Psychiatr Scand [Internet]. 2018 Feb [cited 2018 Jul 9];137(2):157–70. Available from: http://www.ncbi.nlm.nih.gov/pubmed/29282709

8. Modesti MN, Rapisarda L, Capriotti G, Del Casale A. Functional Neuroimaging in Dissociative Disorders: A Systematic Review. J Pers Med. 2022;12(9).

9. Reinders AATS, Nijenhuis ERS, Quak J, Korf J, Haaksma J, Paans AMJ, et al. Psychobiological characteristics of dissociative identity disorder: a symptom provocation study. Biol Psychiatry. 2006;60(7):730–40.

10. Reinders AATS, Marquand AF, Schlumpf YR, Chalavi S, Vissia EM, Nijenhuis ERS, et al. Aiding the diagnosis of dissociative identity disorder: pattern recognition study of brain biomarkers. Br J Psychiatry [Internet]. 2019 Sep 7 [cited 2019 Oct 25];215(3):536–44. Available from: http://www.ncbi.nlm.nih.gov/pubmed/30523772

11. Bado P, Engel A, de Oliveira-Souza R, Bramati IE, Paiva FF, Basilio R, et al. Functional dissociation of ventral frontal and dorsomedial default mode network components during resting state and emotional autobiographical recall. Hum Brain Mapp. 2014;35(7):3302–13.

12. Rolls ET. The cingulate cortex and limbic systems for emotion, action, and memory. Brain Struct Funct [Internet]. 2019 Dec 26;224(9):3001–18. Available from: http://link.springer.com/10.1007/s00429-019-01945-2

13. Taïb S, Yrondi A, Lemesle B, Péran P, Pariente J. What are the neural correlates of dissociative amnesia? A systematic review of the functional neuroimaging literature. Vol. 14, Frontiers in Psychiatry. 2023.

14. Lapointe AR, Crayton JW, DeVito R, Fichtner CG, Konopka LM. Similar or disparate brain patterns? The intra-personal EEG variability of three women with multiple personality disorder. Clin EEG Neurosci [Internet]. 2006 Jul;37(3):235–42. Available from: http://journals.sagepub.com/doi/10.1177/155005940603700314

15. Roydeva MI, Reinders AATS. Biomarkers of Pathological Dissociation: A Systematic Review. Neurosci Biobehav Rev [Internet]. 2021;123(1):120–202. Available from: https://doi.org/10.1016/j.neubiorev.2020.11.019

16. Weber DL, Clark CR, McFarlane AC, Moores KA, Morris P, Egan GF. Abnormal frontal and parietal activity during working memory updating in post-traumatic stress disorder. Psychiatry Res - Neuroimaging. 2005;140(1):27–44.

17. Tsai G. Functional magnetic resonance imaging of personality switches in a woman with dissociative identity disorder. Harv Rev Psychiatry [Internet]. 1999;7(2):119–22. Available from: http://www.hrp.oupjournals.org/cgi/doi/10.1093/hrp/7.2.119

18. Lebois LAM, Ross DA, Kaufman ML. I Am Not I: The Neuroscience of Dissociative Identity Disorder. Biol Psychiatry [Internet]. 2022 Feb 1 [cited 2022 May 12];91(3):e11–3. Available from: http://www.ncbi.nlm.nih.gov/pubmed/34961597

19. Schlumpf YR, Nijenhuis ERS, Chalavi S, Weder E V., Zimmermann E, Luechinger R, et al. Dissociative part-dependent biopsychosocial reactions to backward masked angry and neutral faces: An fMRI study of dissociative identity disorder. NeuroImage Clin. 2013;3(July):54–64.

20. Harricharan S, Nicholson AA, Thome J, Densmore M, McKinnon MC, Théberge J, et al. PTSD and its dissociative subtype through the lens of the insula: Anterior and posterior insula resting‐state functional connectivity and its predictive validity using machine learning. Psychophysiology [Internet]. 2020 Jan 10 [cited 2020 Feb 6];57(1):e13472. Available from: http://www.ncbi.nlm.nih.gov/pubmed/31502268

21. Sar V, Koyuncu A, Ozturk E, Yargic LI, Kundakci T, Yazici A, et al. Dissociative disorders in the psychiatric emergency ward. Gen Hosp Psychiatry. 2007;29(1):45–50.

22. Mertens YL, Manthey A, Sierk A, Walter H, Daniels JK. Neural correlates of acute post-traumatic dissociation: a functional neuroimaging script-driven imagery study. BJPsych Open [Internet]. 2022 Jul 10 [cited 2023 Feb 24];8(4):e109. Available from: https://www.cambridge.org/core/product/identifier/S2056472422000655/type/journal_article

23. Gong L, Wang J, Yang X, Feng L, Li X, Gu C, et al. Dissociation between conceptual and perceptual implicit memory: Evidence from patients with frontal and occipital lobe lesions. Front Hum Neurosci. 2016;9(JAN2016):1–7.

24. Blades R, Becerra S, Jordan S, Eusebio B, Heatwole M, Iovine J, et al. The Role of the Insula in Classical and Dissociative PTSD: A Double Case Study. Neurocase [Internet]. 2022;28(2):140–8. Available from: https://doi.org/10.1080/13554794.2021.1978502

25. Reinders AATS, Willemsen ATM, Vissia EM, Vos HPJ, den Boer JA, Nijenhuis ERS. The psychobiology of authentic and simulated dissociative personality states: The full monty. J Nerv Ment Dis [Internet]. 2016 Jun;204(6):445–57. Available from: http://content.wkhealth.com/linkback/openurl?sid=WKPTLP:landingpage&an=00005053-201606000-00006
