# Supplementary material for: Overt and covert processing of self-relevance information in dissociative identity disorder: controlled fMRI study
Source: BJPsych Open. 2025 Dec 26;12(1):e25. doi: 10.1192/bjo.2025.10914 (PMC12835690; doi:10.1192/bjo.2025.10914)
Supplement: Strouza et al. supplementary material 2 — Strouza et al. supplementary material [file S2056472425109149sup002.docx]

**Table 4. Emotional intensity effect (NSt versus NSn): increased and decreased brain activation**

| **L/R** | **Brain region** | **BA** |  | **Within group** | | | | |  | **Between group** | | | | | | | | | | |
| --- | --- | --- | --- | --- | --- | --- | --- | --- | --- | --- | --- | --- | --- | --- | --- | --- | --- | --- | --- | --- |
|  |  |  |  | **DID-G** | | | | |  | **DID-G versus CTRL** | | | | |  | **DID-G versus DID-S** | | | | |
|  |  |  |  | *X* | *Y* | *Z* | *T^*p* | *kE^c* |  | *X* | *Y* | *Z* | *T^*p* | *kE^c* |  | *X* | *Y* | *Z* | *T^*p* | *kE^c* |
| **Emotional intensity effect (NSt versus NSn)** | | | | | | | | | | | | | | | | | | | | |
|  |  |  |  |  |  |  |  |  |  |  |  |  |  |  |  |  |  |  |  |  |
| **Increased brain activation between processing levels** | | | | | | | | | | | | | | | | | | | | |
|  |  |  |  |  |  |  |  |  |  |  |  |  |  |  |  |  |  |  |  |  |
| ***TAIS*** | | | | | | | | | | | | | | | | | | | | |
|  |  |  |  |  |  |  |  |  |  |  |  |  |  |  |  |  |  |  |  |  |
| *Cortical areas* | | | | | | | | | | | | | | | | | | | | |
|  |  |  |  |  |  |  |  |  |  |  |  |  |  |  |  |  |  |  |  |  |
| L | Superior frontal gyrus | BA 8 |  |  |  |  |  |  |  | -4 | 26 | 44 | 4.47 | 28^c |  |  |  |  |  |  |
| L | Inferior temporal gyrus | BA 37 |  |  |  |  |  |  |  | -46 | -43 | -9 | 3.81 | 9 |  |  |  |  |  |  |
| *Subcortical areas* | | | | | | | | | | | | | | | | | | | | |
|  |  |  |  |  |  |  |  |  |  |  |  |  |  |  |  |  |  |  |  |  |
|  | N.S. |  |  |  |  |  |  |  |  |  |  |  |  |  |  |  |  |  |  |  |
|  |  |  |  |  |  |  |  |  |  |  |  |  |  |  |  |  |  |  |  |  |
| ***TRIS*** | | | | | | | | | | | | | | | | | | | | |
|  |  |  |  |  |  |  |  |  |  |  |  |  |  |  |  |  |  |  |  |  |
| *Cortical areas* | | | | | | | | | | | | | | | | | | | | |
|  |  |  |  |  |  |  |  |  |  |  |  |  |  |  |  |  |  |  |  |  |
| L | Superior frontal gyrus | BA 8 |  | -26 | 16 | 40 | 4.50^p | 18^1 |  |  |  |  |  |  |  | -26 | 33 | 44 | 3.70^p | 38^1^c |
| R | Superior frontal gyrus | BA 8 |  | 20 | 19 | 33 | 4.25^p | 8 |  |  |  |  |  |  |  |  |  |  |  |  |
|  |  | BA 6 |  | 24 | -5 | 54 | 3.98^p | 20^2 |  |  |  |  |  |  |  |  |  |  |  |  |
| R | Superior/Middle frontal gyrus | BA 6 |  | 34 | -1 | 58 | 4.24^p | 20^1 |  |  |  |  |  |  |  |  |  |  |  |  |
| L | Superior/Middle frontal gyrus | BA 8 |  | -29 | 23 | 44 | 4.05^p | 18^2 |  |  |  |  |  |  |  | -32 | 26 | 50 | 3.36^p | 38^2^c |
|  |  | BA 6 |  | -26 | 5 | 61 | 4.00^p | 15^2 |  |  |  |  |  |  |  | -36 | 2 | 50 | 4.22 | 60^1^c |
| L | Middle frontal gyrus | BA 6 |  |  |  |  |  |  |  |  |  |  |  |  |  | -32 | -5 | 64 | 3.95 | 14^1 |
|  |  | BA 10 |  | -26 | 47 | 22 | 3.65^p | 8^1 |  |  |  |  |  |  |  | -46 | 47 | 16 | 2.91^p | 28^2 |
|  |  | BA 10 |  | -29 | 40 | 26 | 3.63^p | 8^2 |  |  |  |  |  |  |  | -29 | 44 | 26 | 3.84 | 28^1 |
| L | Superior frontal gyrus/PreCentral gyrus | BA 6 |  | -15 | 2 | 68 | 4.47^p | 15^1 |  |  |  |  |  |  |  | -22 | -12 | 72 | 3.37^p | 14^2 |
| R | PreCentral gyrus | BA 1/5 |  |  |  |  |  |  |  | 2 | -36 | 58 | 4.10 | 28^1^c |  |  |  |  |  |  |
|  |  | BA 3 |  |  |  |  |  |  |  |  |  |  |  |  |  | 38 | -15 | 47 | 3.00^p | 10^2 |
|  |  | BA 4 |  | 16 | -33 | 64 | 3.86^p | 10 |  | -40 | -29 | 61 | 4.05 | 173^3^**c |  |  |  |  |  |  |
| L | PreCentral gyrus | BA 4 |  |  |  |  |  |  |  | -1 | -26 | 54 | 3.46^p | 28^2^c |  |  |  |  |  |  |
| R | PreCentral gyrus/Superior frontal gyrus | BA 6 |  | 10 | 12 | 47 | 6.12 | 32^c |  |  |  |  |  |  |  |  |  |  |  |  |
| R | Post/PreCentral gyrus | BA 1/4 |  | 2 | -36 | 58 | 3.99^p | 18^1 |  | 30 | -26 | 68 | 3.76^p | 8 |  | 48 | -19 | 47 | 3.08^p | 10^1 |
| L | Post/PreCentral gyrus |  |  | -1 | -26 | 54 | 3.89^p | 18^2 |  |  |  |  |  |  |  | -22 | -29 | 68 | 3.71^p | 9 |
| R | Post/PreCentral gyrus/Precuneus | BA 1/4 |  | 2 | -43 | 64 | 3.68^p | 18^3 |  |  |  |  |  |  |  |  |  |  |  |  |
| R | PreCentral gyrus/Superior parietal lobule | BA 4/5 |  |  |  |  |  |  |  |  |  |  |  |  |  | 2 | -36 | 54 | 3.88 | 75^2^c |
| R | Frontal operculum/Superior parietal lobule | BA 4/5 |  |  |  |  |  |  |  |  |  |  |  |  |  | 44 | -1 | 12 | 3.45^p | 10 |
| L | PreCentral gyrus/Superior parietal lobule | BA 5 |  |  |  |  |  |  |  |  |  |  |  |  |  | -12 | -33 | 47 | 5.60 | 75^1^c |
| L | PostCentral gyrus | BA 1 |  | -26 | -33 | 64 | 9.21 | 345^1^**c |  | -46 | -39 | 61 | 4.21 | 173^2^**c |  | -46 | -39 | 61 | 3.50^p | 21^2 |
|  |  | BA 1 |  | -43 | -33 | 54 | 6.09 | 345^3^**c |  |  |  |  |  |  |  | -43 | -22 | 50 | 3.91 | 60^2^c |
| R | PostCentral gyrus | BA 1 |  |  |  |  |  |  |  | 48 | -33 | 54 | 3.54^p | 32^1^c |  |  |  |  |  |  |
|  |  | BA 1 |  |  |  |  |  |  |  | 48 | -29 | 44 | 3.36^p | 32^2^c |  |  |  |  |  |  |
| L | (Anterior) Insula | BA 13 |  | -32 | 19 | 5 | 4.32^p | 17^1 |  |  |  |  |  |  |  |  |  |  |  |  |
|  |  | BA 13 |  | -36 | 12 | -2 | 3.84^p | 17^2 |  |  |  |  |  |  |  |  |  |  |  |  |
| L | Superior parietal lobule | BA 7 |  | -12 | -71 | 64 | 3.94^p | 14^1 |  |  |  |  |  |  |  | -18 | -57 | 54 | 3.50^p | 12^1 |
|  |  | BA 7 |  | -18 | -67 | 61 | 3.92^p | 14^2 |  |  |  |  |  |  |  | -18 | -67 | 61 | 3.00^p | 12^2 |
| R | Middle cingulate gyrus | BA 32 |  |  |  |  |  |  |  |  |  |  |  |  |  | 6 | 2 | 40 | 3.04^p | 27^2 |
| L | Middle cingulate gyrus | BA 32 |  | -4 | 16 | 36 | 3.86^p | 23^1 |  |  |  |  |  |  |  | -8 | 12 | 33 | 3.99 | 26 |
|  |  | BA 33 |  | -1 | 5 | 30 | 3.55^p | 23^2 |  |  |  |  |  |  |  |  |  |  |  |  |
|  |  | BA 24 |  | -1 | -8 | 47 | 4.42^p | 27^c |  |  |  |  |  |  |  | -1 | -5 | 47 | 3.69^p | 27^1 |
| L | Supramarginal gyrus | BA 40 |  | -54 | -26 | 44 | 7.97 | 345^2^**c |  | -50 | -29 | 50 | 4.47 | 173^1^**c |  | -50 | -26 | 47 | 3.69^p | 60^3^c |
|  |  | BA 40 |  |  |  |  |  |  |  |  |  |  |  |  |  | -40 | -39 | 54 | 3.79 | 21^1 |
| L | Angular gyrus | BA 39 |  | -29 | -53 | 44 | 4.87 | 20 |  | -32 | -64 | 36 | 3.42^p | 10 |  |  |  |  |  |  |
| L | Lingual gyrus | BA 18 |  |  |  |  |  |  |  | -4 | -64 | 5 | 3.04^p | 38^3^c |  |  |  |  |  |  |
| R | Superior occipital gyrus | BA 18/19 |  | 16 | -88 | 22 | 4.02^p | 14 |  | -8 | -78 | 19 | 3.65^p | 38^2^c |  | 30 | -88 | 19 | 2.91^p | 12^2 |
| L | Middle/lateral occipital gyrus | BA 19 |  | -50 | -74 | 16 | 4.91 | 19^1 |  |  |  |  |  |  |  | 24 | -78 | 33 | 3.05^p | 8^1 |
|  |  | BA 19 |  | -40 | -78 | 16 | 3.89^p | 19^2 |  |  |  |  |  |  |  | -43 | -78 | 2 | 3.68^p | 12 |
| R | Occipital gyrus | BA 19 |  | 41 | -84 | 30 | 4.13^p | 8 |  |  |  |  |  |  |  | 41 | -88 | 22 | 3.34^p | 12^1 |
| R | Occipital gyrus/Cuneus | BA 19 |  |  |  |  |  |  |  |  |  |  |  |  |  | 16 | -74 | 30 | 3.05^p | 8^2 |
| R | Cuneus | BA 18 |  | 10 | -71 | 26 | 3.63^p | 8 |  | 10 | -78 | 30 | 3.63^p | 41^1^c |  |  |  |  |  |  |
|  |  | BA 18 |  |  |  |  |  |  |  | 10 | -84 | 22 | 3.41^p | 41^3^c |  |  |  |  |  |  |
| R | Superior temporal gyrus | BA 22 |  |  |  |  |  |  |  |  |  |  |  |  |  | 52 | -15 | -2 | 3.12^p | 15^2 |
| R | Tranverse temporal gyrus | BA 41 |  |  |  |  |  |  |  |  |  |  |  |  |  | 41 | -19 | 2 | 4.12 | 15^1 |
| R | Cingulate sulcus | BA 31 |  |  |  |  |  |  |  | 13 | -67 | 26 | 3.58^p | 41^2^c |  | 2 | -29 | 44 | 3.37^p | 75^3^c |
| L | Calcarine sulcus | BA 17 |  | -12 | -74 | 12 | 3.61^p | 11 |  | -12 | -78 | 8 | 3.72^p | 38^1^c |  |  |  |  |  |  |
| L | Parahippocampal gyrus/Frontal operculum | BA 30 |  |  |  |  |  |  |  |  |  |  |  |  |  | -4 | -33 | 5 | 3.49^p | 26^2 |
| R | Parahippocampal gyrus/Frontal operculum | BA 30 |  |  |  |  |  |  |  |  |  |  |  |  |  | 6 | -33 | 2 | 3.42^p | 26^3 |
| *Subcortical areas* | | | | | | | | | | | | | | | | | | | | |
|  |  |  |  |  |  |  |  |  |  |  |  |  |  |  |  |  |  |  |  |  |
| R | Thalamus |  |  | 2 | -22 | 8 | 9.48^**p | 200^1^**c |  | 6 | -19 | 12 | 3.95 | 63^2^c |  |  |  |  |  |  |
| L | Thalamus |  |  | -8 | -29 | 5 | 6.86 | 200^2^**c |  | -8 | -33 | 5 | 4.45 | 63^1^c |  | -15 | -33 | 5 | 4.24 | 26^1 |
|  |  |  |  |  |  |  |  |  |  | -8 | -19 | 16 | 3.31^p | 63^3^c |  |  |  |  |  |  |
| R | Putamen |  |  |  |  |  |  |  |  | 30 | 2 | -6 | 3.85 | 8 |  |  |  |  |  |  |
| L | Ventral Diencephalon |  |  | -1 | -22 | -9 | 6.25 | 200^3^**c |  | -4 | -22 | -12 | 4.12 | 10^c |  |  |  |  |  |  |
| L | Cerebellar Vermal Lobules I-V |  |  |  |  |  |  |  |  | -4 | -53 | -9 | 3.06^p | 8 |  |  |  |  |  |  |
| R | Hippocampus |  |  |  |  |  |  |  |  |  |  |  |  |  |  | 24 | -36 | 2 | 3.84 | 12 |
| L | Lateral Ventrical |  |  |  |  |  |  |  |  |  |  |  |  |  |  | -1 | 9 | 12 | 3.28^p | 8^1 |
| R | Lateral Ventrical |  |  |  |  |  |  |  |  |  |  |  |  |  |  | 6 | 5 | 5 | 2.85^p | 8^2 |
|  |  |  |  |  |  |  |  |  |  |  |  |  |  |  |  |  |  |  |  |  |
| **Increased brain activation between identity states** | | | | | | | | | | | | | | | | | | | | |
|  |  |  |  |  |  |  |  |  |  |  |  |  |  |  |  |  |  |  |  |  |
| ***Overt processing*** | | | | | | | | | | | | | | | | | | | | |
|  |  |  |  |  |  |  |  |  |  |  |  |  |  |  |  |  |  |  |  |  |
| *Cortical areas* | | | | | | | | | | | | | | | | | | | | |
|  |  |  |  |  |  |  |  |  |  |  |  |  |  |  |  |  |  |  |  |  |
| R | Precentral gyrus | BA 4 |  |  |  |  |  |  |  | 27 | -19 | 36 | 3.81 | 11 |  |  |  |  |  |  |
| *Subcortical areas* | | | | | | | | | | | | | | | | | | | | |
|  |  |  |  |  |  |  |  |  |  |  |  |  |  |  |  |  |  |  |  |  |
| L | Caudate nucleus (tail) |  |  |  |  |  |  |  |  |  |  |  |  |  |  | -29 | -46 | 16 | 3.67^p | 13 |
|  |  |  |  |  |  |  |  |  |  |  |  |  |  |  |  |  |  |  |  |  |
| ***Covert processing*** | | | | | | | | | | | | | | | | | | | | |
|  |  |  |  |  |  |  |  |  |  |  |  |  |  |  |  |  |  |  |  |  |
| *Cortical areas* | | | | | | | | | | | | | | | | | | | | |
|  |  |  |  |  |  |  |  |  |  |  |  |  |  |  |  |  |  |  |  |  |
| R | Superior frontal gyrus | BA 8/6 |  | 2 | 23 | 47 | 7.00 | 43^c |  |  |  |  |  |  |  | 2 | 19 | 50 | 3.38^p | 17 |
|  |  | BA 9 |  | 10 | 40 | 26 | 6.28 | 31^1^c |  | 10 | 40 | 26 | 4.16 | 11 |  |  |  |  |  |  |
| L | Superior frontal gyrus | BA 6 |  | -22 | 16 | 61 | 5.01 | 9 |  | -18 | 16 | 61 | 3.60^p | 8 |  |  |  |  |  |  |
| L | Middle/Superior frontal gyrus | BA 8 |  | -54 | 12 | 44 | 4.50^p | 90^3^**c |  |  |  |  |  |  |  | -22 | 30 | 40 | 4.23 | 39^1^c |
| L | Middle frontal gyrus | BA 10 |  | -26 | 47 | 26 | 5.29 | 37^1^c |  |  |  |  |  |  |  | -29 | 44 | 30 | 3.22^p | 39^2^c |
|  |  | BA 10 |  |  |  |  |  |  |  |  |  |  |  |  |  | -36 | 54 | 22 | 3.36^p | 8 |
|  |  | BA 46 |  | -36 | 37 | 19 | 5.26 | 37^2^c |  |  |  |  |  |  |  | -36 | 40 | 22 | 3.01^p | 39^3^c |
|  |  | BA 8 |  | -26 | 33 | 44 | 5.00 | 37^3^c |  |  |  |  |  |  |  |  |  |  |  |  |
|  |  | BA 46 |  | -54 | 33 | 16 | 4.59 | 26^2^c |  |  |  |  |  |  |  |  |  |  |  |  |
| L | Inferior frontal gyrus | BA 44 |  | -57 | 12 | 16 | 5.19 | 22^1^c |  |  |  |  |  |  |  | -57 | 9 | 16 | 3.69^p | 14^1 |
|  |  | BA 44 |  | -50 | 9 | 22 | 3.35^p | 22^2^c |  |  |  |  |  |  |  | -60 | 16 | 8 | 3.44^p | 14^2 |
|  |  | BA 45 |  | -57 | 26 | 5 | 4.73 | 26^1^c |  | -57 | 26 | 8 | 3.83 | 17^1 |  |  |  |  |  |  |
|  |  | BA 46 |  |  |  |  |  |  |  | -50 | 30 | 16 | 3.59^p | 17^2 |  |  |  |  |  |  |
|  |  | BA 46 |  |  |  |  |  |  |  | -46 | 40 | 12 | 3.33^p | 17^3 |  |  |  |  |  |  |
|  |  | BA 44 |  |  |  |  |  |  |  |  |  |  |  |  |  | -50 | 16 | 26 | 3.38^p | 13^1 |
|  |  | BA 44 |  |  |  |  |  |  |  |  |  |  |  |  |  | -46 | 5 | 22 | 3.23^p | 13^2 |
| R | PreCentral gyrus | BA 6 |  | 6 | -19 | 50 | 4.64 | 9 |  |  |  |  |  |  |  |  |  |  |  |  |
| L | PreCentral gyrus | BA 6/4 |  | -40 | -1 | 54 | 5.38 | 90^1^**c |  |  |  |  |  |  |  | -40 | -1 | 50 | 4.00 | 41^1^c |
|  |  | BA 6/4 |  | -40 | 2 | 33 | 4.54^p | 90^2^**c |  |  |  |  |  |  |  | -46 | 2 | 47 | 3.55^p | 41^2^c |
|  |  | BA 6 |  |  |  |  |  |  |  |  |  |  |  |  |  | -50 | 9 | 36 | 3.29^p | 21 |
| R | PostCentral gyrus | BA 1 |  | 34 | -33 | 61 | 4.45^p | 13^2 |  | 44 | -29 | 50 | 3.82 | 15 |  | 34 | -39 | 58 | 4.03 | 24^1 |
| L | (Anterior) Insula | BA 13 |  | -32 | 23 | 5 | 4.55^p | 12 |  |  |  |  |  |  |  | -40 | 12 | 5 | 4.24 | 29^c |
| L | Superior parietal lobule | BA 7 |  | -15 | -71 | 54 | 5.86 | 119^3^**c |  |  |  |  |  |  |  | -15 | -71 | 58 | 3.19^p | 18 |
|  |  | BA 1 |  | -32 | -60 | 36 | 4.18^p | 14^2 |  |  |  |  |  |  |  |  |  |  |  |  |
| R | Superior parietal lobule | BA 7 |  | 38 | -39 | 58 | 4.71 | 13^1 |  |  |  |  |  |  |  |  |  |  |  |  |
| L | Precuneus/Superior parietal lobule | BA 7 |  | -8 | -74 | 61 | 6.99 | 119^1^**c |  |  |  |  |  |  |  | -8 | -46 | 61 | 4.45 | 115^1^**c |
|  |  | BA 7 |  | -8 | -74 | 47 | 6.70 | 119^2^**c |  |  |  |  |  |  |  | -15 | -53 | 61 | 4.28 | 115^2^**c |
| R | Precuneus/Superior parietal lobule | BA 7 |  | 13 | -71 | 64 | 4.92 | 26^1^c |  |  |  |  |  |  |  | 16 | -67 | 61 | 4.96 | 54^1^c |
|  |  | BA 7 |  | 6 | -67 | 54 | 3.45^p | 26^2^c |  |  |  |  |  |  |  | 6 | -60 | 64 | 3.43^p | 54^2^c |
|  |  | BA 7 |  | 10 | -74 | 50 | 3.41^p | 26^3^c |  |  |  |  |  |  |  | 30 | -53 | 64 | 3.90 | 24^2 |
| L | Supramarginal gyrus/Inferior parietal lobule | BA 40 |  |  |  |  |  |  |  |  |  |  |  |  |  | -40 | -39 | 54 | 3.44^p | 20^1 |
|  |  | BA 40 |  |  |  |  |  |  |  |  |  |  |  |  |  | -46 | -43 | 61 | 3.40^p | 20^2 |
|  |  | BA 40 |  |  |  |  |  |  |  |  |  |  |  |  |  | -57 | -33 | 36 | 3.40^p | 8 |
| R | Angular gyrus | BA 39 |  | 52 | -50 | 44 | 6.16 | 21^1^c |  |  |  |  |  |  |  |  |  |  |  |  |
|  |  | BA 39 |  | 52 | -60 | 40 | 3.59^p | 21^2^c |  |  |  |  |  |  |  |  |  |  |  |  |
| L | Angular gyrus | BA 39 |  | -36 | -57 | 44 | 4.51^p | 14^1 |  |  |  |  |  |  |  |  |  |  |  |  |
| R | Lateral occipital gyrus | BA 18 |  | 41 | -91 | -2 | 5.94 | 12^1 |  |  |  |  |  |  |  |  |  |  |  |  |
|  |  | BA 18 |  | 34 | -98 | 2 | 3.74^p | 12^2 |  |  |  |  |  |  |  |  |  |  |  |  |
| L | Lateral occipital gyrus | BA 19 |  |  |  |  |  |  |  | -50 | -78 | 22 | 3.80 | 12 |  |  |  |  |  |  |
| L | Occipital gyrus | BA 17 |  | -12 | -105 | 2 | 3.85^p | 9 |  |  |  |  |  |  |  |  |  |  |  |  |
| L | Cingulate gyrus | BA 23 |  | -1 | -19 | 26 | 4.22^p | 21^1^c |  |  |  |  |  |  |  |  |  |  |  |  |
|  |  | BA 24 |  | -1 | -5 | 30 | 3.83^p | 21^2^c |  |  |  |  |  |  |  |  |  |  |  |  |
|  |  | BA 24/33 |  | -15 | 12 | 40 | 3.69^p | 9^2 |  |  |  |  |  |  |  |  |  |  |  |  |
|  |  | BA 24/32 |  | -4 | 9 | 30 | 4.03^p | 9^1 |  |  |  |  |  |  |  | -4 | 9 | 30 | 4.31 | 73^1^c |
|  |  | BA 23 |  | -1 | -33 | 30 | 3.81^p | 26^3^c |  | -4 | -15 | 26 | 3.11^p | 9^1 |  |  |  |  |  |  |
|  |  | BA 23 |  | -1 | -53 | 30 | 3.96^p | 26^2^c |  |  |  |  |  |  |  |  |  |  |  |  |
|  |  | BA 31 |  |  |  |  |  |  |  |  |  |  |  |  |  | -12 | -33 | 40 | 3.76^p | 115^3^**c |
| R | Cingulate gyrus | BA 23 |  | 6 | -15 | 33 | 3.75^p | 21^3^c |  |  |  |  |  |  |  |  |  |  |  |  |
|  |  | BA 32 |  | 13 | 23 | 26 | 3.77^p | 31^2^c |  |  |  |  |  |  |  | 10 | 5 | 40 | 3.40^p | 73^2^c |
|  |  | BA 23 |  | 2 | -43 | 30 | 4.95 | 26^1^c |  | 2 | -8 | 30 | 3.07^p | 9^2 |  | 6 | -15 | 30 | 4.40 | 25 |
|  |  | BA 32/33 |  |  |  |  |  |  |  |  |  |  |  |  |  | 2 | 30 | 19 | 3.31^p | 73^3^c |
| R | Calcarine sulcus | BA 17 |  | 20 | -71 | 12 | 5.06 | 9 |  |  |  |  |  |  |  |  |  |  |  |  |
| L | Calcarine sulcus | BA 17 |  | -18 | -71 | 8 | 3.58^p | 11^1 |  |  |  |  |  |  |  |  |  |  |  |  |
|  |  | BA 17 |  | -12 | -74 | 12 | 3.27^p | 11^2 |  |  |  |  |  |  |  |  |  |  |  |  |
| *Subcortical areas* | | | | | | | | | | | | | | | | | | | | |
|  |  |  |  |  |  |  |  |  |  |  |  |  |  |  |  |  |  |  |  |  |
| R | Thalamus |  |  |  |  |  |  |  |  |  |  |  |  |  |  | 16 | -15 | -6 | 3.91 | 20 |
|  |  |  |  |  |  |  |  |  |  |  |  |  |  |  |  |  |  |  |  |  |
|  |  |  |  |  |  |  |  |  |  |  |  |  |  |  |  |  |  |  |  |  |
| **Decreased brain activation between processing levels** | | | | | | | | | | | | | | | | | | | | |
|  |  |  |  |  |  |  |  |  |  |  |  |  |  |  |  |  |  |  |  |  |
| ***TAIS*** | | | | | | | | | | | | | | | | | | | | |
|  |  |  |  |  |  |  |  |  |  |  |  |  |  |  |  |  |  |  |  |  |
| *Cortical areas* | | | | | | | | | | | | | | | | | | | | |
|  |  |  |  |  |  |  |  |  |  |  |  |  |  |  |  |  |  |  |  |  |
| L | Precentral gyrus | BA 4 |  |  |  |  |  |  |  |  |  |  |  |  |  | -40 | -5 | 40 | 4.10 | 10 |
| R | PostCentral gyrus | BA 1 |  |  |  |  |  |  |  |  |  |  |  |  |  | 41 | -26 | 50 | 3.98 | 8 |
| L | Occipital gyrus | BA 18 |  | -12 | -95 | 5 | 4.54^p | 8 |  |  |  |  |  |  |  |  |  |  |  |  |
| R | Occipital gyrus/Calcarine sulcus | BA 18/17 |  | 20 | -102 | 5 | 6.20 | 12 |  |  |  |  |  |  |  |  |  |  |  |  |
| R | Middle temporal gyrus | BA 21 |  |  |  |  |  |  |  | 44 | -36 | 2 | 4.63 | 22 |  |  |  |  |  |  |
| *Subcortical areas* | | | | | | | | | | | | | | | | | | | | |
|  |  |  |  |  |  |  |  |  |  |  |  |  |  |  |  |  |  |  |  |  |
|  | N.S. |  |  |  |  |  |  |  |  |  |  |  |  |  |  |  |  |  |  |  |
|  |  |  |  |  |  |  |  |  |  |  |  |  |  |  |  |  |  |  |  |  |
| ***TRIS*** | | | | | | | | | | | | | | | | | | | | |
|  |  |  |  |  |  |  |  |  |  |  |  |  |  |  |  |  |  |  |  |  |
| *Cortical areas* | | | | | | | | | | | | | | | | | | | | |
|  |  |  |  |  |  |  |  |  |  |  |  |  |  |  |  |  |  |  |  |  |
|  | N.S. |  |  |  |  |  |  |  |  |  |  |  |  |  |  |  |  |  |  |  |
| *Subcortical areas* | | | | | | | | | | | | | | | | | | | | |
|  |  |  |  |  |  |  |  |  |  |  |  |  |  |  |  |  |  |  |  |  |
|  | N.S. |  |  |  |  |  |  |  |  |  |  |  |  |  |  |  |  |  |  |  |
|  |  |  |  |  |  |  |  |  |  |  |  |  |  |  |  |  |  |  |  |  |
| **Decreased brain activation between identity states** | | | | | | | | | | | | | | | | | | | | |
|  |  |  |  |  |  |  |  |  |  |  |  |  |  |  |  |  |  |  |  |  |
| ***Overt processing*** | | | | | | | | | | | | | | | | | | | | |
|  |  |  |  |  |  |  |  |  |  |  |  |  |  |  |  |  |  |  |  |  |
| *Cortical areas* | | | | | | | | | | | | | | | | | | | | |
|  |  |  |  |  |  |  |  |  |  |  |  |  |  |  |  |  |  |  |  |  |
| R | Superior frontal gyrus | BA 6/8 |  | -12 | 16 | 58 | 5.60 | 15 |  |  |  |  |  |  |  |  |  |  |  |  |
|  |  | BA 6 |  |  |  |  |  |  |  |  |  |  |  |  |  | 13 | -5 | 50 | 3.78^p | 10 |
| L | Middle frontal gyrus | BA 8 |  | -32 | 26 | 44 | 4.85 | 9 |  | -32 | 26 | 47 | 3.88 | 15 |  |  |  |  |  |  |
|  |  | BA 9 |  |  |  |  |  |  |  |  |  |  |  |  |  | -29 | 30 | 44 | 4.84 | 20 |
| R | PreCentral gyrus | BA 4 |  |  |  |  |  |  |  |  |  |  |  |  |  | 41 | -19 | 50 | 3.65^p | 22 |
| L | Pre/PostCentral gyrus | BA 1/4 |  |  |  |  |  |  |  |  |  |  |  |  |  | -18 | -33 | 61 | 3.59^p | 20^2 |
| L | Superior parietal lobule | BA 7 |  |  |  |  |  |  |  |  |  |  |  |  |  | -8 | -39 | 64 | 3.96 | 20^1 |
| R | Inferior parietal lobule/Supramarginal gyrus | BA 40 |  |  |  |  |  |  |  | 44 | -33 | 19 | 3.60^p | 11 |  |  |  |  |  |  |
| L | Parietal operculum | BA 40 |  |  |  |  |  |  |  |  |  |  |  |  |  | -29 | -29 | 19 | 4.52 | 14 |
| L | Lateral occipital gyrus/Cuneus | BA 18 |  |  |  |  |  |  |  |  |  |  |  |  |  | -1 | -98 | 16 | 3.84 | 11 |
| R | Cuneus/Calcarine sulcus | BA 18/17 |  | 13 | -105 | 2 | 4.10^p | 9 |  |  |  |  |  |  |  |  |  |  |  |  |
| R | Calcarine sulcus | BA 17 |  |  |  |  |  |  |  |  |  |  |  |  |  | 13 | -84 | 8 | 4.20 | 26 |
|  |  | BA 17 |  |  |  |  |  |  |  |  |  |  |  |  |  | 6 | -71 | 19 | 3.56^p | 13 |
| *Subcortical areas* | | | | | | | | | | | | | | | | | | | | |
|  |  |  |  |  |  |  |  |  |  |  |  |  |  |  |  |  |  |  |  |  |
|  | N.S. |  |  |  |  |  |  |  |  |  |  |  |  |  |  |  |  |  |  |  |
|  |  |  |  |  |  |  |  |  |  |  |  |  |  |  |  |  |  |  |  |  |
| ***Covert processing*** | | | | | | | | | | | | | | | | | | | | |
|  |  |  |  |  |  |  |  |  |  |  |  |  |  |  |  |  |  |  |  |  |
| *Cortical areas* | | | | | | | | | | | | | | | | | | | | |
|  |  |  |  |  |  |  |  |  |  |  |  |  |  |  |  |  |  |  |  |  |
|  | N.S |  |  |  |  |  |  |  |  |  |  |  |  |  |  |  |  |  |  |  |
| *Subcortical areas* | | | | | | | | | | | | | | | | | | | | |
|  |  |  |  |  |  |  |  |  |  |  |  |  |  |  |  |  |  |  |  |  |
| R | Caudate nucleus (tail) |  |  | -26 | -43 | 22 | 4.87 | 8 |  |  |  |  |  |  |  |  |  |  |  |  |

**Notes**: DID = dissociative identity disorder; DID-G = genuine/diagnosed DID; DID-S = DID simulating controls; CTRL = paired control group; ﻿(X, Y, Z) = MNI coordinates in mm; L/R = Left/Right side of the brain; BA = Broadmann area; NSn = non-self-relevant neutral stimuli; NSt = non-self-relevant trauma related stimuli; TAIS = trauma-aware identity state; TRIS = trauma-related identity state; N.S. = no significanceT = T-value statistic; kE = cluster size in voxels (one voxel is 26262 mm)

^1 = first peak voxel; ^2 = second peak voxel; ^3 = third peak voxel

^**c = 0.05 corrected for multiple comparisons at cluster level; ^c = clustersize obtained p = 0.005 uncorrected; ^**p = 0.05 corrected for multiple comparisons at peak level; ^*p = also at 0.001 uncorrected for multiple comparison at peak level; ^p = only at 0.005 uncorrected for multiple comparison at peak level
